# Single-nucleus RNA sequencing-based construction of a hippocampal neuron atlas in mice with epileptic cognitive impairment

**DOI:** 10.1016/j.isci.2024.111065

**Published:** 2024-09-28

**Authors:** Jia-Qi Ma, Lu Wang, Yue Zhang, Yong-Qian Bian, Xiao-Peng Qu, Li-Jia Song, Chao Wang, Li Gao, Qi-Xing Fang, De-Chang Zhao, Liang-Liang Shen, Bei Liu

**Affiliations:** 1 Department of Neurosurgery, Tangdu Hospital, Airforce Military Medical University, Xi’an, China; 2 Department of Plastic and Burn Surgery, Tangdu Hospital, Airforce Military Medical University, Xi’an, China; 3 Department of Pediatrics, Tangdu Hospital, Airforce Military Medical University, Xi’an, China; 4 Department of Biochemistry and Molecular Biology, Airforce Military Medical University, Xi’an, China; 5 College of Life Sciences, Northwest University, Xi’an, Shaanxi 710069, China

**Keywords:** Molecular biology, Neuroscience, Omics, Transcriptomics

## Abstract

The hippocampus plays a critical role in learning and memory, and mice with epileptic cognitive impairment exhibit hippocampal atrophy. However, there is still a lack of research on the hippocampal cell atlas related to these disorders. Here, we utilized snRNA-seq to characterize the transcriptomic changes in hippocampal neurons of drug-resistant epilepsy (DRE) cognitive-impaired mice. The intercellular heterogeneity of 20 subpopulations of neurons was analyzed, focusing on aspects such as cell communication, gene expressions, GO and KEGG enrichment analysis, and module gene set analysis. Based on the degree of relevance to synaptic biological functions, the subpopulations associated with cognitive impairment (ExN1, 3, 8 and InN1, 6) were preliminarily identified. We also identified some key biomarkers in DRE cognitive-impaired mice, such as Ptprz1 and Calb1. Finally, we integrate and validate our dataset using identified well-annotated marker genes in the hippocampal region, further supporting the functional annotation of neuronal subpopulations.

## Highlights

• The heterogeneity of hippocampal neurons in RE-CI mice was dissected
• The potential functions of neuron subpopulations were analyzed
• Various study sets validated the heterogeneity of neuron subpopulation

## Graphical abstract

**Figure:**
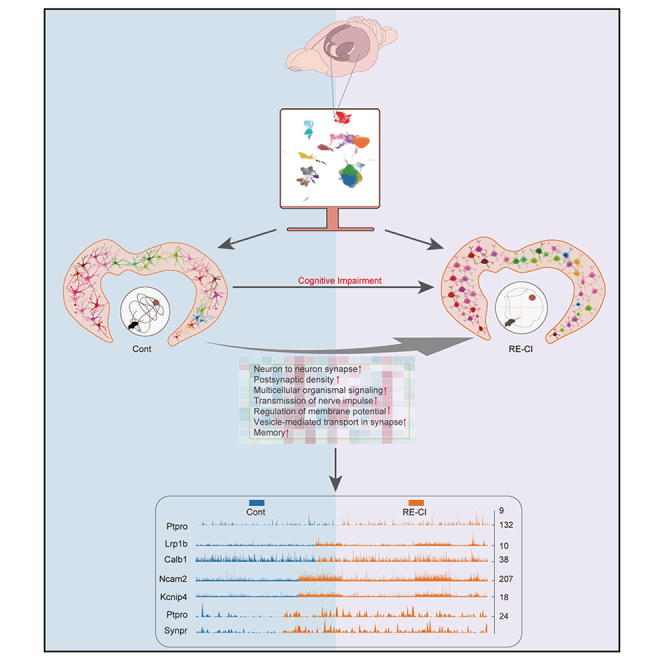

## Introduction

Epilepsy is a prevalent dynamic neurological disorder, and its association with cognitive comorbidities has been clinically established.^1^ Cognitive impairment serves as a crucial factor leading to reduced quality of life in individuals with epilepsy.^2^ Moreover, it represents a central symptom in various neurodegenerative diseases, including temporal lobe epilepsy (TLE),^3^ Alzheimer’s disease (AD),^4^ Parkinson’s disease (PD),^5^ and schizophrenia (SCZ).^6^ Despite this, the mechanism behind seizure-related cognitive dysfunction remains poorly understood. Epilepsy-induced cognitive deficits can manifest as temporary, persistent, or progressive conditions. Brief interruptions in cognitive coding processes may be linked to focal or systemic epileptic discharges. At the same time, neuronal plasticity, recombination, germination, and cellular metabolic damage associated with the onset of seizures serve as primary determinants of progressive cognitive decline.^7^ Current understanding suggests a potential correlation between epileptic cognitive impairment and hippocampal neuronal changes.^8^

The hippocampus is considered the center of cognition,^9^ and plays a crucial role in memory formation.^10^^,^^11^ Studies show that epileptogenic injury enhances neurogenesis in the hippocampus.^12^^,^^13^ Additionally, developmental irregularities in neonatal neurons are linked to cognitive dysfunction, cognitive impairment, and emotional processing.^14^ As the central hub of communication between neurons, synapses participate in enabling rapid transmission of information between neurons. Their synaptic plasticity and delicate structural framework are the essential basis of learning and memory. They also have an impact on cognitive changes.^15^^,^^16^ In particular, studies on learning and memory related to the postsynaptic dense area (PSD) and the hippocampus reaffirms this.^17^ Therefore, hippocampal neuronal types and synaptic information transmission may play an essential role in the occurrence of cognitive impairment.

Recently, differences in gene expression of major excitatory cell types in the hippocampus along the long axis of normal mice and cell type-specific variation in the anterior hippocampus (Ahc) and posterior hippocampus (Phc) in humans have been identified through bulk RNA sequencing.^18^^,^^19^ While there have also been relevant studies on cell types in quite a few neurodegenerative diseases,^20^^,^^21^^,^^22^^,^^23^ it is of great significance to analyze the changes in the neuronal transcriptome of drug-resistant epileptic cognitive impairment (RE-CI) mice from the perspective of single-nuclear RNA sequencing (snRNA-seq). Moreover, the impact of interconnections between different hippocampal neuronal subpopulations on cognitive impairment remains unclear and necessitates further investigation. The emergence of snRNA-seq technology now enables the molecular characterization of distinct cell types in the human brain.^24^ This innovative approach allows for the assessment of cell type-specific gene expression patterns about different brain disorders, including those involving memory impairment.^20^^,^^25^ Our study aimed to analyze the subtypes of neurons in the hippocampus of mice with DRE-related cognitive impairment, utilizing snRNA-seq technology. Additionally, we presented the comprehensive transcriptional profile of hippocampal neuron types in mice with DRE-related cognitive impairment. By elucidating the specific functions of various cell types, our findings provide valuable insights for future research on cognitive impairment related to DRE.

## Results

### Evaluation of behavioral alterations related to drug-resistant epilepsy mice

**Figure 1:**
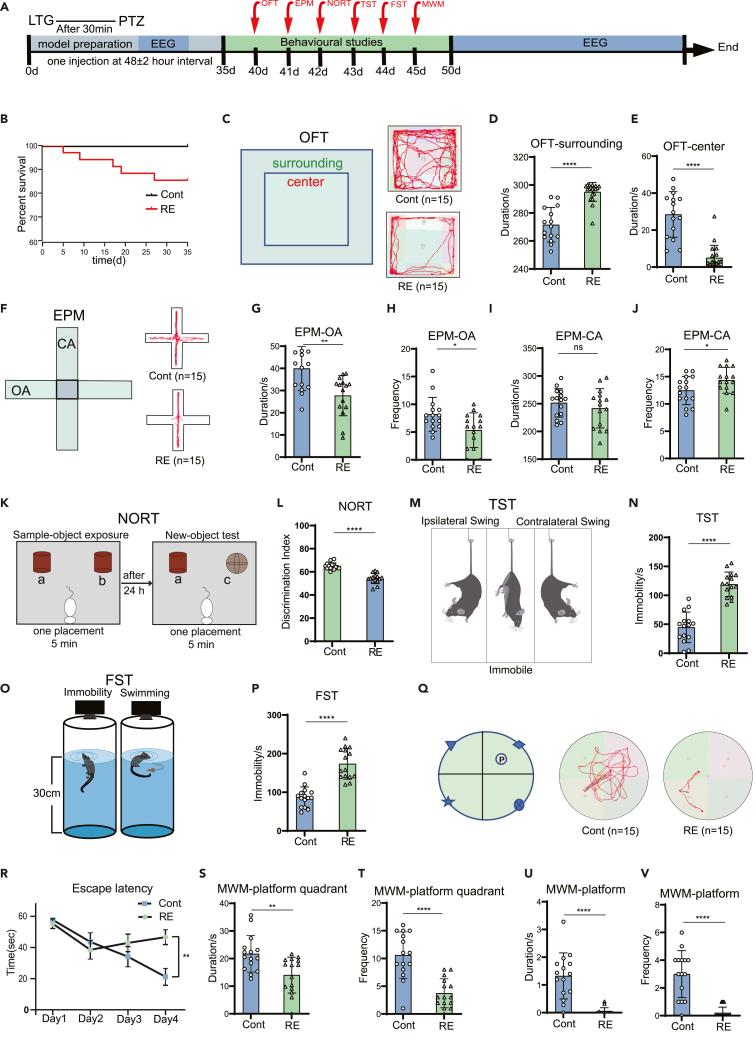
Lamotrigine-resistant epileptic mice developed anxiety, depressive behavior, and cognitive impairment (A) Mouse modeling time and behavioral study schedule. EEG monitoring was performed during the modeling period and after the behavioral experiment. (B) Survival curves of lamotrigine-resistant epileptic mice (*n* = 35) and control mice (*n* = 35) during modeling. (C–E) Open field experiment. (C) Open field experiment diagram and representative behavior track diagram of the two groups of mice. The chart shows the duration of the surrounding area (D) and the central area (E) in the open field experiment of the mice (*n* = 15). (F–J) Elevated Cross Maze experiment. (F) Schematic diagram of the elevated cross maze experiment and the representative behavioral trajectory diagram of the two groups of mice. The chart shows the duration (G) and frequency (H) of the open arm and the duration (I) and frequency (J) of the closed arm in the elevated cross maze experiment of the mice (*n* = 15). (K) Schematic diagram of the novel object recognition test. (L) The chart shows the discrimination index of the two groups of mice in the novel object recognition test (*n* = 15). (M) Schematic diagram of tail suspension test. (N) The chart shows the immobility time of the two groups of mice in the tail suspension test (*n* = 15). (O) Schematic diagram of the forced swimming test. (P) The graph shows the immobility time of the two groups of mice in the forced swimming test (*n* = 15). (Q–V) Morris Water maze test. (Q) Schematic diagram of the Morris water maze experiment and the representative behavioral trajectory diagram of the two groups of mice. (R) Training phase: The escape latency time of the two groups of mice in the first four days of the training phase (*n* = 15). (S–V) Test phase: The chart shows the duration (S) and frequency (T) of the two groups of mice in the platform quadrant (*n* = 15). The chart shows the duration (U) and frequency (V) of the two groups of mice in the platform (*n* = 15). The data are expressed as mean ± SD, ∗*p* < 0.05, ∗∗*p* < 0.01, ∗∗∗*p* < 0.001, ∗∗∗∗*p* < 0.0001.

The prevalence of psychiatric comorbidities is high among patients with epilepsy, particularly those with refractory focal epilepsy.^26^ Anxiety and depression frequently coexist in individuals with epilepsy.^27^ In our study, we successfully established a DRE mouse model by the intraperitoneal injection of lamotrigine and pentylenetetrazol for 35 consecutive days (Figure 1A). Five mice died during modeling due to persistent seizures (Figure 1B). Behavioral testing commenced on the 5th day after successful modeling, wherein we evaluated anxiety and depression-like behaviors in lamotrigine-resistant epileptic mice using the open field test (OFT), elevated plus maze (EPM), tail suspension test (TST), and forced swim test (FST), respectively. In OFT (Figure 1C), DRE mice exhibited a significant increase in duration in the surrounding region (*p* < 0.0001, Figure 1D), and a notable decrease in duration in the central area (*p* < 0.0001, Figure 1E). During the EPM test (Figure 1F), DRE mice exhibited a notable decrease in duration (*p* < 0.01, Figure 1G) and frequency (*p* < 0.05, Figure 1H) in the open arm (OA). There was no difference in the duration of the closed arm (Figure 1I), but the frequency increased significantly (*p* < 0.01, Figure 1J). These findings suggest the presence of anxiety-like behavior in DRE mice. Furthermore, in TST and FST, DRE mice exhibited a significantly longer immobility time compared to the control group (*p* < 0.0001, Figures 1M–1P), indicating DRE mice may exhibit depression-like behavior.

Impaired learning and memory function is a common comorbidity associated with epilepsy.^28^ To evaluate the deficits related to the hippocampus, we employed the novel object recognition test (NORT) and the Morris Water Maze test (MWM). In NORT (Figure 1K), the discrimination index of the RE group was significantly lower than the Cont group (*p* < 0.0001, Figure 1L). In MWM (Figure 1Q), during the training phase, the control mice exhibited a gradual decrease in latency to find the submerged platform in the water maze as the training time increased. In contrast, mice with DRE exhibited a gradual increase in the time taken to locate the platform, with a notable delay observed on the fourth day of training (*p* < 0.01, Figure 1R). On the fifth day, a probe trial was conducted with the platform removed. The duration (*p* < 0.01, Figure 1S) and frequency (*p* < 0.0001, Figure 1T) of DRE mice in the platform quadrant were significantly reduced. In addition, the duration (*p* < 0.0001, Figure 1U) and frequency (*p* < 0.0001, Figure 1V) of DRE mice in the platform region were also significantly reduced. This suggests that cognitive function is impaired after lamotrigine resistance.

### Observation of changes in hippocampal tissue of drug-resistant epileptic cognitive impairment mice through electroencephalogram, LSCI, magnetic resonance imaging, and TEM

**Figure 2:**
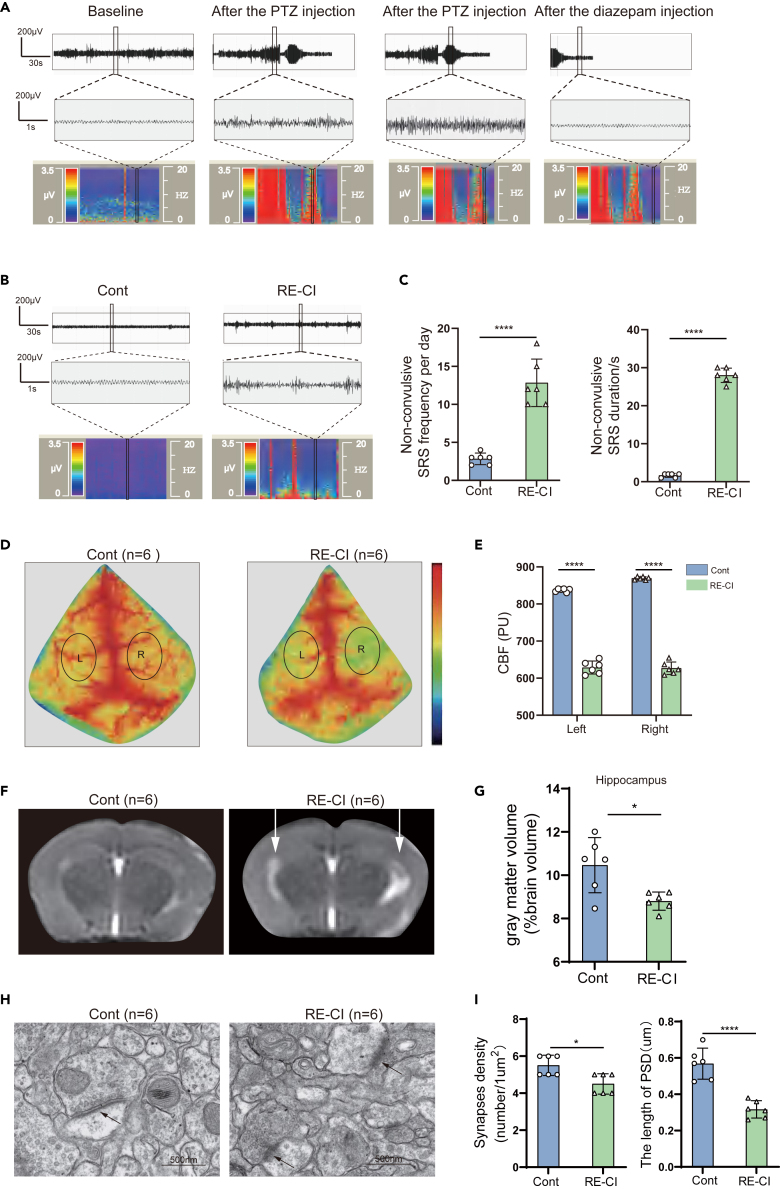
Evaluation of the characteristic alterations in the brain of RE-CI mice (A) Typical EEG recordings of seizures in mice during model preparation (From left to right, baseline EEG, onset of seizures after PTZ injection, persistent seizures, and EEG after Diazepam injection; The bottom is the representative power spectrum). (B) A representative electroencephalogram of non-convulsive seizure activity in the RE-CI mice, with a representative power spectrum at the bottom. (C) Frequency and duration of non-convulsive SRS between control and RE-CI mice (*n* = 6). (D) Representative LSCI imaging of bilateral cerebral cortex blood flow monitoring in the control group and RE-CI mice. (L: left, R: right). (E) Blood flow in the upper hippocampal cortex of the RE-CI mice on both sides. cerebral blood flow (CBF); related to the product of the average movement speed and concentration of red blood cells in the tissue sample, measured in PU. (F) T2-weighted magnetic resonance images of control and RE-CI mice. The RE-CI mice hippocampus shows high magnetic resonance imaging signal strength (arrow). (G) The proportion of gray matter in the total brain volume of the hippocampus in the control and RE-CI mice. (H) Control representative images of synapses with RE-CI mice. The black arrows represent synapses. Scale bar, 500nm. (I) Number of synapses and PSD length (*n* = 3) compared with RE-CI mice. (C–H) Data are expressed as mean ± SD, *n* = 6, ∗∗∗∗*p* < 0.0001 (using unpaired bilateral t-test).

To further investigate cognitive impairment in epilepsy, We used the novel object recognition test and the Morris water maze test to screen drug-resistant epilepsy cognitive impairment (RE-CI) mice We further utilized Racine’s scale to assess the seizure grade of mice and employed EEG monitoring to determine seizures in mice. After injecting pentylenetetrazol into mice, high-frequency and high amplitude spike like epileptic waves were observed through EEG monitoring (Figure 2A). Previous studies have classified SRS into convulsive and non-convulsive types.^29^ By analyzing the EEG recordings, we confirmed the presence of non-convulsive SRS in RE-CI mice (Figure 2B). Expectedly, the frequency (*p* < 0.0001) and duration (*p* < 0.0001) of non-convulsive SRS were significantly higher in RE-CI mice (Figure 2C). Intriguingly, when monitoring cerebral blood flow, we observed that the bilateral cerebral blood flow above the hippocampus was significantly reduced in RE-CI mice (*p* < 0.0001, Figures 2D and 2E), suggesting a potential association with structural changes in the hippocampus. To assess hippocampal structural damage, we conducted 3D magnetic resonance imaging (MRI) scans on mice from the RE-CI and control group (Figure 2F). Compared to the control group, The total volume of the hippocampus in RE-CI mice significantly decreased (*p* < 0.05, Figure 2G). Previous research demonstrated that the length of postsynaptic density (PSD) is related to memory formation and retrieval.^30^ Using transmission electron scanning, we observed a reduction in the number of synapses (*p* < 0.05) and postsynaptic density (*p* < 0.0001) in RE-CI mice (Figures 2H and 2I).

### Integration analysis of bilateral hippocampal tissue of drug-resistant epileptic cognitive impairment mice by single-nuclear RNA sequencing

**Figure 3:**
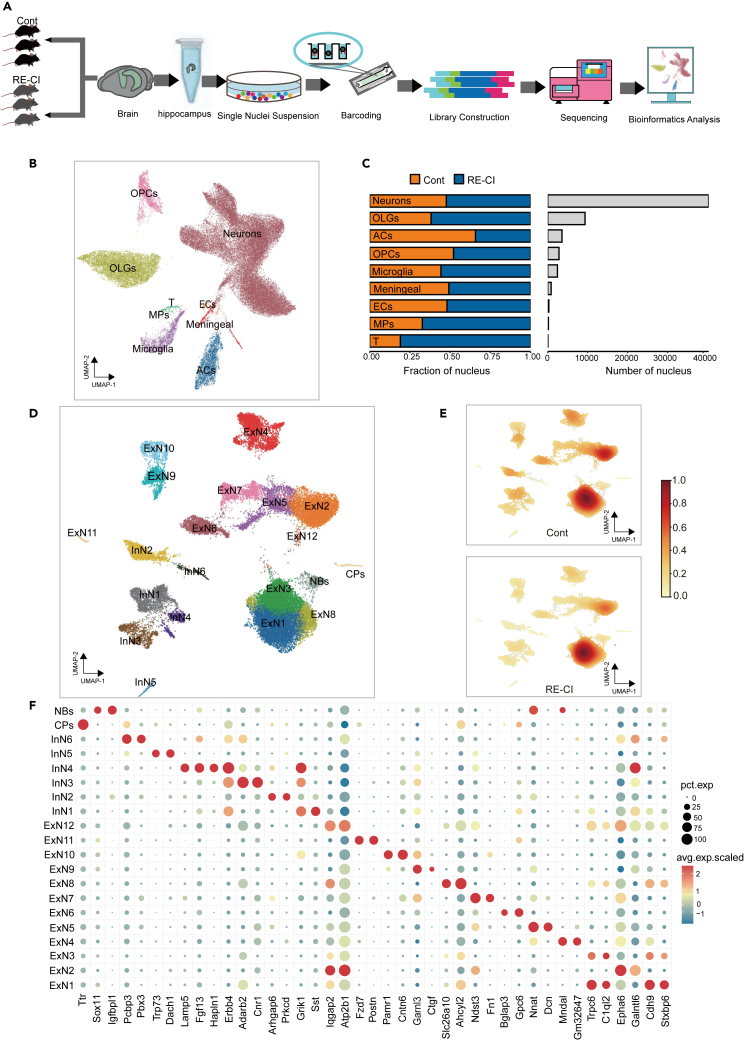
snRNA-Seq identifies major neuronal cell types in the hippocampus of RE-CI mice (A) Flow chart and bioinformatics analysis of snRNA-seq in the mouse hippocampus, Cont, *n* = 3, RE-CI, *n* = 3 mouse. (B) Two-dimensional uniform manifold approximation and projection (UMAP) diagrams of unsupervised clustering results for the major cell types in the hippocampus, including neurons, astrocytes, microglia, T cells, oligodendrocytes, oligodendrocyte precursor cells (OPCs), endothelial cells (ECs), mononuclear phagocytes (MPs), meninges. (C) Bar chart of inter-group distribution and nucleus proportion of each cluster cell. (D) Unsupervised clustering of neuronal cells showed 20 subtypes of neuronal cells, ExN1-12, InN1-6, CPs, and NBs (see Figure S3 for detailed clustering information). (E) nucleus density diagram of neuronal cell subtype 20. (F) Bubble map showing average expression of marker genes for 20 neuronal cell types.

To better understand the underlying mechanisms and cellular composition during the RE-CI process, snRNA-seq was performed on bilateral hippocampal tissues from three RE-CI and 3 control mice, respectively (Figures 3A and S1), and 59,726 nuclei from these mice were analyzed, with 31,509 nuclei in the RE-CI group and 28,217 nuclei in the control group (Table S1). We identified nine major cell populations, including neurons, oligodendrocytes (OLGs), oligodendrocyte precursor cells (OPCs), astrocytes (ACs), endothelial cells (ECs), microglia, macrophages (MPs), T cells, and Meningeal (Figure 3B; Table S2). After visualization using UMAP, the distinct clusters were identified for each cell population (Figure S2A; Table S3). By comparing the number of cells, hippocampal tissues from RE-CI mice contained 21,120 enriched neurons, while those from normal mice contained 19,187 enriched neurons (Table S1). We found that neurons were the most abundant cell type in the hippocampus, with a higher percentage of neurons in the RE-CI group (Figure 3C). A total of 279 differential transcripts were screened for differential analysis between neuronal groups (Table S4).To further characterize the subtypes of neurons, we classified them into excitatory neurons, inhibitory neurons, neuroblasts, and chorioplexus cells based on identified marker genes (Figure S2B). In addition, we validated our data using the hippocampal dentate gyrus (DG) and Cornu ammonis (CA) marker genes from Cembrowski et al., 2018; Cembrowski et al., 2016. (Table S5). We found that the known marker genes (*PFKP*, *TYRO3*) in mouse hippocampal CA2 and CA3 regions were expressed in InN1, InN2, InN3, InN4, InN6, and ExN2, ExN4, ExN5, ExN7, ExN9, ExN10, and ExN 12. The marker gene (*SATB2*) in the hippocampal CA1 region is expressed in ExN6. In addition, the marker gene (*MAML2*) of granulosa cells in the DG region is expressed in ExN1, ExN8, and ExN 12 (Figure 8C; Table S5). Using UMAP clustering, we identified 20 neuronal transcriptomically significant clusters (Figures 3D and 3F; Table S3). The clusters were then annotated based on specific genes expressed in each unsupervised cluster. Quantitative analysis of the density of 20 hippocampal neuronal subpopulations between RE-CI and control mice showed that the number of nuclei in the RE-CI group was up-regulated in ExN1, ExN2, ExN3, ExN8, and InN6. In contrast, the number of nuclei in InN2 was significantly down-regulated (Figures 3E, S2C, and S2D; Table S1). Moreover, *Arc*, an apoptosis inhibitor, was found to inhibit apoptosis,^31^ while its corresponding molecule *Bax* promoted mitochondrial breakdown.^32^ We found that Arc was highly expressed in ExN2, ExN4, ExN6, ExN7, ExN9, ExN12, and InN2. (Figure S3A). *NR4A1*, an immediate-early gene, can regulate the expression of *FOS* and promote cell activation.^33^ Thus we found that ExN10 was deactivated in the RE-CI group, ExN12 was activated in the RE-CI group, and ExN7 showed enrichment between these two groups (Figure S3B). These distinct states suggest that the subpopulations of neurons have different roles after RE-CI. ExN10 potentially has a protective mechanism against cognitive impairment, while ExN12 may have an opposing role. These preliminary findings indicate that different subpopulations of neurons potentially play distinct roles in the RE-CI group.

### Molecular association of neuronal subpopulations with cognitive impairment

**Figure 4:**
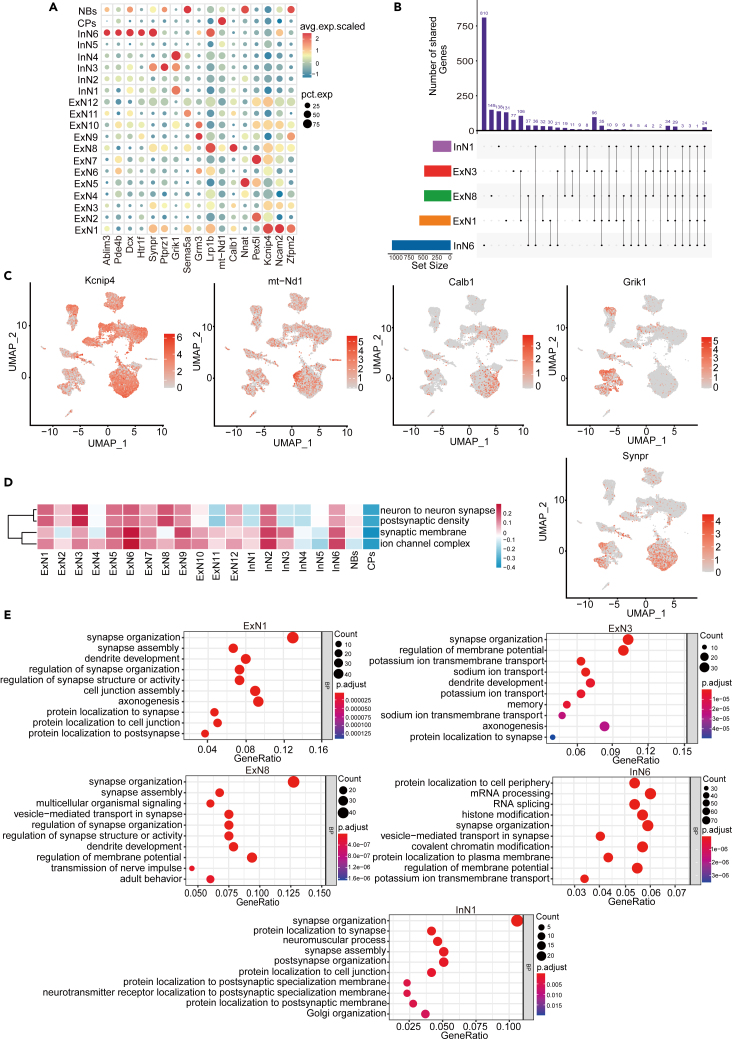
20 subpopulations of neurons are involved in signal transduction related to memory impairment and synaptic functions (A) Bubble maps showing the expression levels of different cluster-specific gene markers. (B) UpSetR plots highlight upregulated DEGs of ExN1, 3, 8, and InN1, 6, including DEGs that are specific to clusters or shared between clusters. The top column shows the number of DEGs per cluster. Lines between clusters highlight shared DEGs, and dots represent unique DEGs. (C) The expression of selected marker genes in significant cell types. Kcnip4: ExN1, ExN3, ExN7, ExN8, and ExN12; mt-Nd1: CPs; Calb1: ExN8; Grik1: InN1, InN3 and InN4; Synpr: ExN1, InN3 and InN6. (D) GSVA enrichment analysis of ExN1, ExN3, ExN5, ExN6, ExN8, and ExN9, as well as InN1, InN2, and InN6 neuron subpopulations, including four pathways, demonstrating enrichment among the primary biological pathways of memory impairment in the 20 subpopulations. (E) GO BP functional enrichment diagrams of ExN1, ExN3, and ExN8 and InN1 and InN6 subpopulations, with horizontal coordinates as GeneRatio and vertical coordinates as GO Term, circle color representing the adjusted *p*-values, and circle size representing the number of differential genes.

We then attempt to annotate the functions of each of the 20 subpopulations of neurons and elucidate the biological processes that each subpopulation may be involved in. Considering the enrichment of multicellular organismal signaling and transmission of nerve impulse pathways to a relatively high gene ratio, we performed GSEA analysis with gene sets of these two pathways. The results showed that the curve was enriched at the top (NES>0), indicating that both pathways were significantly enriched in the RE-CI group, and signaling and nerve transmission may be related to cognitive impairment in the RE-CI group (Figures S4A–S4C). We further found some specific genes related to cognitive impairment in these maker genes in the subdivided cell subpopulations (Figure 4A; Table S6). For example, several similarly expressed genes were identified in ExN1 and ExN3, including *Kcnip4*, *Ncam2,* and *Zfpm2*. *Kcnip4* is correlated with Alzheimers disease (AD) and plays a key role in synaptic growth, synaptic plasticity, neuron proliferation, and neuronal differentiation.^34^^,^^35^ *Kcnip4* in GO terms belongs to a potassium channel coding gene in potassium channel-related membrane potential of different neurons.^36^ The single nucleotide polymorphism (SNP) of *Ncam2*, nerve cell adhesion molecule 2, is highly expressed in synapses and correlated with Aβ levels in human cerebrospinal fluid during cognitive impairment in AD.^37^ *Zfpm2* has been reported as a protective factor for AD,^38^ indicating the inhibitory effect on cognitive impairment. Thus, ExN1 and ExN3 probably participate in the information transmission of synaptic resting potential to excitatory potential transition, and the changes in their expression levels are significantly correlated with cognitive impairment (Figure 4A). *Calb1* has been reported to affect the long-term enhanced activity and learning function of the hippocampus and plays a positive role in promoting neurodegenerative diseases.^39^^,^^40^ *Lrp1b* is expressed in neurons of the central nervous system, and its high expression is related to memory impairment.^41^ The high expression of *Calb1* and *Lrp1b* in ExN8 of RE-CI mice may be involved in the learning and memory function of the hippocampus (Figure 4A). *Grik1* accelerates cognitive impairment in mice,^42^ consistent with our sequencing results that *Grik1* was highly expressed in InN1 and InN4, which were associated with the generation of cognitive impairment phenotype (Figure 4A). InN6 highly expressed *Ablim3*, *Pde4b*, *Dcx* and *Htr1f* (Figure 4A). *Ablim3* down-regulation can improve the accuracy of remote memory.^43^ *Pde4b* can reverse the learning and memory defects in hippocampal synaptic plasticity caused by TBI.^44^ *Dcx* is highly correlated with the generation of immature neurons.^45^ The induction of *Dcx* and *Bdnf* has been demonstrated as a good approach for the treatment of memory impairment.^46^ *Htr1f* is mainly expressed in excitatory and inhibitory neurons and is involved in the signal transduction process between neurons.^47^ Thus, InN6 seems to be more involved in the transmission of learning and memory (Figure 4A). The high expression of *Pex5I* in other neuronal subpopulations such as ExN2 and ExN7 has been confirmed the reverse effect on learning and LTP.^48^^,^^49^ The high expression of Nnat in ExN5 and NBs is found in the early stages of neurogenesis and potentially plays an important role in neurogenesis.^50^ *mt-Nd1* is significantly down-regulated in AD-related cognitive impairment, so our results further verified that *mt-Nd1* highly expressed subpopulations are involved in the occurrence of cognitive impairment (Figure 4A).^51^ *Ptprz1* transgenic showed impaired working memory.^52^ Results suggest that *Grm3*, expressed in ExN9 and ExN10, plays a specific role in long-term depression and neuroprotection, and potentially be involved in cognitive impairment.^53^ *Sema5a*, which was highly expressed in ExN11 and NBs, is involved in axon guidance. Dysregulation of *Sema5a* might be the basis of cognitive deficits in FUS transgenic mice^54^ (Figure 4A). Further, we analyzed the location of molecular subpopulations related to cognitive impairment based on protein expression levels. Consistent with Figure 4A, we identified expression of *Kcnip4* in ExN1, ExN2, ExN3, ExN6, ExN7, ExN8 and ExN10, expression of mt-Nd1 and *Calb1* in ExN8, expression of *Grik1* in InN1, InN4, and ExN10, and expression of *Synp*r in ExN1, ExN8, InN1, InN3, and InN6 (Figure 4C). Due to the subtle function of choroid plexus cells and neuroblasts, the following analysis will exclude these two cell subpopulations.

Thus, we made a brief functional understanding of the subpopulations according to the specific molecules of cognitive impairment. Then we would discuss the specific functions of each subpopulation more specifically through functional analysis.

GO analysis of cell component enrichment showed that excitatory neurons and individual inhibitory neurons participated in synaptic-related pathways (Figure 4D). Then, Biological process analysis schat almost all of these subpopulations had a standard function on the composition of synaptic tissue.

GO terms associated with synaptic composition and synaptic components were enriched in ExN1 (Figure S6A). Many genes were enriched in these pathways (Figure S6B). These genes may be unique to the pathway. Still, some are shared by inter-pathway connections, and the most shared genes are associated with synaptic tissue, cell connections, and synaptic assembly, suggesting the function of ExN1 may be involved in synaptic plasticity (Figure S6C). We found that the genes shared by ExN1 and highly expressed among groups include *Dgkb* (Figures S6D and S6E). Studies have shown that *Dgkb* KO mice have cognitive impairment, irritable behavior, and susceptibility to epilepsy,^55^ suggesting EXN1 affects memory transmission.

ExN2 was also involved in membrane potential regulation and transmembrane transport of calcium ions, as well as in the composition of ion channel complexes (Figure S7A). In pathways related to synaptic organization and membrane potential, we observed the highest number of genes that regulate and enrich these functions (Figure S7B). For identifying functional modules, we found that specific pathways exhibited a higher enrichment effect for shared genes, such as *Epha7* and *Ryr3* (Figure S7D). *Epha7* is known to be involved in the developmental functions of the nervous system.^56^ Studies have demonstrated that the *Ryr3* subtype is crucial in synaptic plasticity and hippocampal-dependent memory processes.^57^ We discovered that *Epha7* and *Ryr3* were implicated in multiple synapse-related pathways (Figure S7E). By integrating the pathways with significant differences after RE-CI, we compared the number of upregulated differential genes and shared genes between RE-CI and the control group. RE-CI group exhibited substantial differences and a greater number of shared genes in pathways associated with synaptic activity or structural regulation (Figure S7C), suggesting ExN2 may primarily participate in functions related to cognitive impairment by modulating synaptic plasticity.

ExN3 showed enrichment in terms such as synaptic membrane, postsynaptic specialization, regulation of membrane potential and memory (Figures 4E and S8A), and a significant number of genes enriched in the membrane potential regulation pathway (Figure S8B). Moreover, many genes were shared between these pathways and others, including potassium ion transmembrane transport (Figure S8C). However, we did not identify molecules directly linked to pathways that exhibited notable differences (Figure S8E). The heatmap displayed significant expressions of *Bdnf*, *Dgkb*, and *Penk* (Figure S8D). *Bdnf* is a brain-derived neurotrophic factor. Studies indicated that *Bdnf* undergoes abnormal upregulation in models of epilepsy.^58^ Increasing *Bdnf* expression facilitates synaptic reconstruction.^58^ According to the Uniprot database, *Dgkb* exhibits specific expression in the brain and regulates neuron-specific morphological changes, including neurite branching and neurite spine formation. *Penk* upregulation inhibits GABA in the striatum, thereby enhancing striatal output.^59^ Thus, ExN3 may predominantly regulate changes in neuron morphology and membrane potential, consequently influencing memory-related functions (Figure 4E).

ExN8 was directly enriched in vesicle−mediated transport in synapses and involved in neurotransmitter release more directly (Figures 4E and S13A). Similarly, we observed many genes enriched in vesicle−mediated transport in synapses (Figure S13B). For example, *Nrxn3* plays a crucial role in synaptic function related to normal aging and cognitive decline in AD, as indicated by functional network analysis^60^ (Figure S13D). The synaptic-related pathways were interconnected and clustered with a high degree of coincidence, which has been verified. *Nrxn3* is an intermediate hub of vesicle-mediated synapses and synaptic organization with significant differences (Figure S13E). Furthermore, in the context of the Gene Ontology pathway, we directly observed gene sharing in neurodegenerative diseases related to cognitive impairment, such as Huntington's disease, Alzheimer's disease (AD), and Parkinson's disease (Figure S13C). These findings suggest that ExN8 potentially plays an essential regulatory role in cognitive impairment.

InN1 participated in protein localization to the synapse, enabling neurotransmitters to find receptors more quickly after release, thus initiating a series of biological responses in downstream pathways more accurately and rapidly (Figures 4E and S18A). We also identified molecules with significant differences in protein localization to synapse pathway (Figure S18D). Studies have shown that *Dlgap1*, a postsynaptic density protein gene, can affect the executive function of attention-deficit hyperactivity disorder.^61^ *Gpc6* can polarize astrocytes into a neuroprotective phenotype, ultimately leading to neural regeneration.^62^ Similarly, we found abundant genes related to protein localization to synapse, which also regulate other pathways and exhibit shared genes (Figures S18B and S18C). *Dlgap1* and *Gpc6* connect various pathways, co-regulating the function of InN1 (Figure S18E).

The functions of InN2 and InN6 were relatively similar and comprehensive, involving pathways such as vesicle−mediated transport in the synapse, regulation of membrane potential, and protein localization to the synapse, with a higher number of gene enrichments (Figures 4E, S19A, and S23A). According to the Uniprot database, the differential expression of *Ptprd* in InN2 can induce presynaptic and postsynaptic differentiation of neurons through cross-synaptic mediated interactions with *IL1RAP* and *IL1RAPL1* (Figures S19D and S19E). Its gene enrichment was primarily based on axonogenesis and molecular connections with other pathways (Figures S19B and S19C). InN6 exhibited the highest concentration of differential genes and significant pathways related to protein localization to the cell periphery (Figures S23A and S23B). The same upregulated genes regulated additional pathways, such as protein localization to the plasma membrane and vesicle−mediated transport in synapse. The mutual regulation of shared genes between pathways formed the basis for fully exerting the function of this cell subpopulation (Figures S23C and S23E). Reduction of *Nectin3*, a gene associated with synaptic cell adhesion molecules, induces defects in social and spatial memory and dendrites in mice^63^ (Figure S23D). These molecules were also involved in the association of various interconnected network channels (Figure S23E).

The remaining subpopulations, including ExN4, ExN5, ExN6, ExN7, ExN9, and ExN10, appeared to primarily contribute to the formation of synaptic tissue (Figures S9–S12, S14, and S15). Notably, ExN6 was enriched in the GO term postsynaptic density, which aligns with the changes observed in Figure 2. It is possible that ExN6 was responsible for the observed phenotypic changes (Figure S11). ExN7 may have a more vital involvement in axon generation (Figure S12), while ExN9 may play a role in signaling between synaptic tissues (Figure S14). ExN11 was more closely associated with developmental functions such as dendrite development (Figure S16). ExN12 and InN4 did not seem to enrich any relevant pathways and might not play substantial roles (Figures S17 and S21). InN3 was also involved in the regulation of cell morphogenesis (Figure S20), and InN5 participated in clathrin-dependent endocytosis, which was crucial for signal transmission (Figure S22). Interestingly, ExN12 and InN4 did not show significant differences in path enrichment (Figures S17 and S21). Additionally, our analysis of KEGG results revealed the involvement of specific subpopulations, such as ExN1 and InN6, in the Long−term potentiation pathway (Figures S5A and S5E). ExN3 and ExN8 were enriched in glutamatergic synapses (Figures S5B and S5C), while InN1 participated in long-term depression (Figure S5D). In summary, the neuron subpopulations that were potentially important in cognitive impairment were ExN1, ExN3, ExN8, InN1, and InN6. Some subpopulations may have overlapping functions, as the number of differential genes shared between ExN1 and ExN3 was almost equivalent to the number of specific genes expressed by ExN1, indicating similar molecular expression (Figure 4A). Functional analysis, combined with the number of unique or shared genes, suggested that ExN1, ExN3, ExN8, InN1, and InN6 shared more genes, indicating their potential participation in common pathway regulation. The abundance of unique genes in InN6 suggested its irreplaceability (Figure 4B). It is possible that not only these several subpopulations but also the remaining subpopulations share similar functions. These findings highlight the need for the functional categorization of neuronal subpopulations.

### Module analysis of neurons by secondary clustering

**Figure 5:**
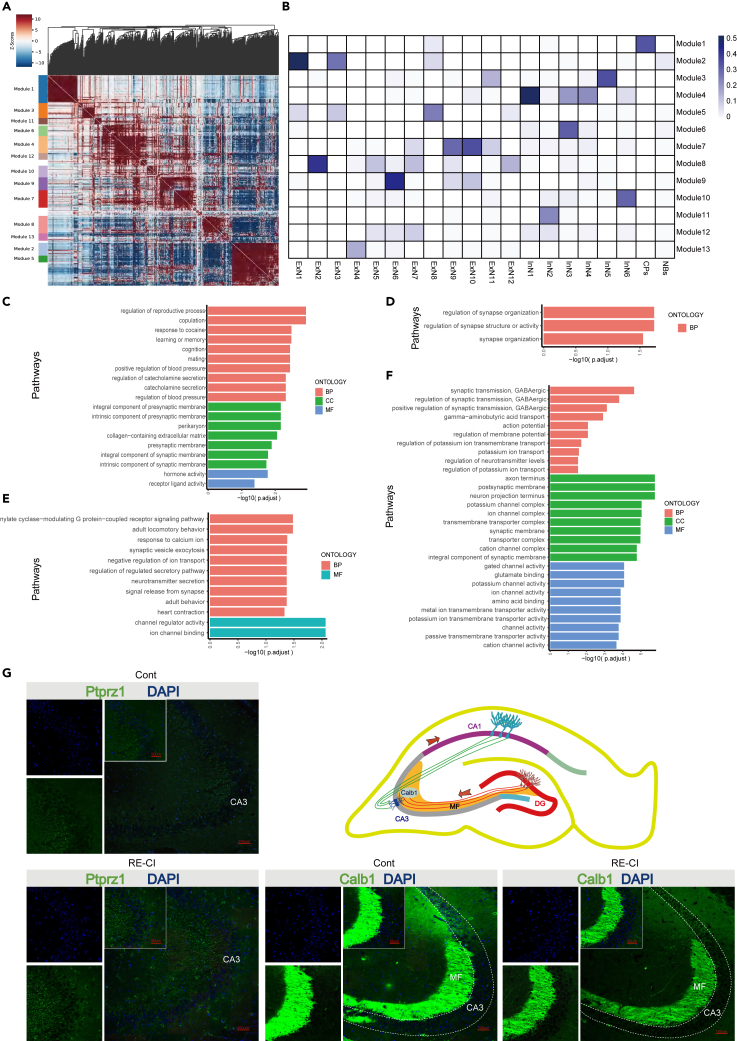
Module analyses the mechanisms by which specific subpopulations regulate functions related to memory impairment (A) Gene correlation heatmap, with the notes on the left representing gene collections. Neurons are divided into modules according to the same category of functions, and each Module is a gene set. The figure shows the corresponding results between the Module and neuron subpopulation. M1: CPs; M2: ExN1, ExN3; M3: InN5; M4: InN1, InN3 and InN4; M5: ExN1, ExN3 and ExN8; M6: InN3; M7: ExN9, ExN10; M8: ExN2; M9: ExN6; M10: InN6; M11: InN2; M12: ExN7; M13: ExN4. (B) Similarity analysis between Module and subpopulation. The darker the color, the more similar the subpopulation and Module gene set are. (C–F) Bar graph showing the gene ontology (GO) pathways rich in Modules 4, 5, 6, and 10. (C) M6 GO analysis. (D) M5 GO analysis. (E) M10 GO analysis. (F) M4 GO analysis. (G–I) Representative immunofluorescence images of *PTPRZ1*, *NCAM2*, and *Calb1* and co-staining of DAPI in the hippocampal region of mice. The schematic diagram shows that the change of *Calb1* in the moss fiber region from DG to CA3 plays a vital role in the RE-CI process. Scale: 100μm; Scale: 50μm (magnification).

To annotate the function of subpopulations, we employed another clustering method. Module analysis for the functional annotation of neuron subpopulations. Neuron subpopulations were classified based on similar functions determined by differentially expressed genes among neuron cells. These subpopulations were divided into 13 modules according to gene sets (Figure 5A). This approach offered the advantage of being more functionally oriented than mechanical grouping. The dimensionality reduction map of gene set scores established a link between subpopulations and Modules (Figure S24A), requiring mapping analysis between Modules and subpopulations. Similarity analysis between Modules and subpopulations was performed to identify which subpopulations might be enriched in the same gene cluster (Figure 5B). The results indicated that the previously mentioned ExN1 and ExN3 mapped to module (M) 2, further confirming the abundance of shared differential genes depicted in Figure 4B. Additionally, ExN1, ExN3, and ExN8 mapped to M5, while InN1, InN3, and InN4 mapped to M4. InN6 was mapped to M10 (Figure 5B). Following the mapping of subpopulations to Modules, functional analysis of modules was conducted to elucidate the function of specific classes of neurons.

The findings revealed that M6 was correlated with learning or memory and cognition (Figures S30A and S30B), with a relatively high number of enriched genes in these pathways (Figure S30E). Moreover, M6 was retrospectively mapped to InN3 (Figure 5B), and molecules enriched in the path, such as *Cnr1*, were found to be associated with the age of Huntington’s disease onset. Insulin-like growth factor 1 (*Igf1*) is a significant regulator of the aging process from rodents to humans, and a deficiency in *Igf1* has also been linked to cognitive dissonance.^64^ Vip stimulates the proliferation and differentiation of brain neurons during neurogenesis^65^ (Figure S30C). Furthermore, significant differences were observed in the learning and memory pathway, with enriched hub molecules involved in regulating other pathways (Figures S30D and S30F). The unique and shared genes of each pathway remained consistent, indicating that these molecules interacted among pathways (Figures S30F and S30G). These findings suggest that InN3 may be involved in learning and memory-related functions by participating in neurogenesis (Figure 5C).

The neuron subpopulations ExN1, ExN3, and ExN8 identified in M5 appear to play a significant role in the regulation of synapse organization and regulation of synapse structure or activity (Figures 5D, S29A, and S29B). A total of three pathways were enriched, including the regulation of synapse organization, regulation of synapse structure or activity, and synapse organization, involving a total of three molecules, each of which is involved in all three pathways (Figures S29D and S29G). These pathways showed high expression of *Epha7*, which has been reported to be crucial for synaptic function. Studies have demonstrated that in cultures lacking *EphA7*, the maturation of pyramidal neurons is delayed, while enhanced synaptic function has been observed when *EphA7* is present^66^ (Figures S29C and S29F). Furthermore, *EphA7* appears to simultaneously regulate these three pathways (Figures S29C and S29E).

The pathways associated with M10 include response to calcium ion, synaptic vesicle exocytosis, neurotransmitter secretion, negative regulation of ion transport, and signal release from the synapse (Figures 5B, S34A, and S34B). These pathways exhibited more enriched genes (Figure S34E), indicating their significance. We observed distinct differences in the ion channel binding pathways enriched by M10, with several differentially enriched genes interacting with other pathways (Figures S34D and S34F). Additionally, the genes enriched in this pathway were also involved in different pathways (Figure S34C). Notably, we identified the expression of fibroblast growth factor 12 (*Fgf12*) within this pathway, which may serve as an essential regulator of neuronal network activity and has been associated with developmental and epileptic encephalopathy (DEE).^67^ *Fgf12* was found to regulate other pathways as well (Figures S34E and S34G), emphasizing the role of the InN6 subpopulation, which is consistent with previous findings. GO analysis also indicated that the complex functions associated with InN6 may play a significant role in cognitive impairment (Figure 5E), highlighting the importance of this subpopulation.

M4 primarily played a role in the synaptic regulation of synaptic transmission, GABAergic, as well as in the transmembrane transporter complex, indicating the involvement of InN1, InN3, and InN4 subpopulations (Figure 5F), and significant enrichment in the GABAergic transmission pathway (Figures S28A and S28B). We observed many shared and differentially enriched genes in these pathways (Figures S28E and S28G). Upon analyzing these genes, we noted high expression of *Grik1*, which was found to be enriched in multiple pathways. Increased levels of *Grik1* mRNA and protein, encoding the ionotropic glutamate receptor kainate-1 (*Grik1*), have been specifically associated with schizophrenia (Figure S28C). The interconnectedness among the pathways (Figure S28D) is closely linked to *Grik1*, which plays a role in almost every path to fulfill specific functions (Figures S28C and S28F). These findings suggest that *Grik1* is a critical factor in the functionality of the M4 population. Furthermore, KEGG enrichment analysis of both M4 and M10 revealed the presence of the cAMP signaling pathway, an important signaling molecule for long-term potentiation (LTP) and memory formation^68^ (Figures S5F and S5G). This indicates the involvement of cognitive impairment in the subpopulations associated with M4 and M10.

We also found that M1 was predominantly involved in the composition and function of complexes, such as active ion transmembrane transporter activity and active transmembrane transporter activity (Figures S25A, S25B, and S25E). These pathways exhibited high enrichment levels, and shared gene expression was observed between them (Figures S25C and S25G). The shared genes participate in the mutual regulation of pathways (Figure S25D), with one example being the expression of *Slc6a20a*, which encodes the *GluR4* subunit of the AMPA receptor present in excitatory glutamatergic synapses and is crucial for learning and memory^69^ (Figure S25C). This molecule also plays a vital role in regulating multiple pathways (Figure S25F).

The functional implications of other modules were relatively straightforward. For example, M2 is involved in the excitatory postsynaptic potential and regulation of postsynaptic membrane potential (Figure S26). M3 suggests that InN5 may be associated with neuronal migration, as indicated by the expression of genes such as *Lhx1*, *Ndnf*, and *Cxcr4* (Figures 5B and S27). M7 indicates that ExN9, ExN10, and ExN11 contribute to the composition of the presynaptic membrane (Figures 5B and S31). M8 suggests that ExN2 may be involved in the long−term synaptic depression and negative regulation of cytosolic calcium ion concentration (Figures 5B and S32). M9 and M12 suggest that ExN6 was involved in synapse organization or the composition of ion channel complexes (Figures 5B, S33, and S35). M13 suggests that ExN4 may be affected by transport vesicle and postsynaptic density (Figures 5B and S36).

We conducted the inter-group analysis to identify molecules with significant differences in the hippocampus in the RE-CI group. We speculated that the differential expression of these molecules might impact cognitive impairment. To verify this, we performed immunofluorescence staining on brain sections from the RE-CI group and the control group. Surprisingly, we observed strong fluorescence of *Calb1* in the mossy fibers (MF) of the medial region of CA3 in the hippocampus (Figure 5G). MF plays a crucial role in connecting granule cells of the dentate gyrus and CA3 for information transmission (Figure 5G). This synaptic connection affects the encoding of contextual memory.^70^ The cAMP signaling pathway is a cyclic adenosine monophosphate (cAMP)-dependent signaling pathway, which has been shown to enhance neurotransmitter release and promote brain function.^71^ Studies have demonstrated that long-term potentiation (LTP) between MF is mediated by enhanced vesicle fusion,^72^ suggesting that MF has a biological basis for its role in memory generation and transmission. Our results indicate that *Calb1* is highly enriched in ExN8 after RE-CI, suggesting that ExN8 may be partly involved in the composition of specific MF structures involved in transmitting memory signals from the dentate gyrus to CA3. Additionally, GO enrichment analysis of ExN8 (Figure 4E) revealed information related to vesicular transport and synaptic organization. This finding highlights the unique role of ExN8 in cognitive impairment. *Ptprz1* has been primarily studied in tumors, particularly gliomas, where it inhibits tumor signaling pathways. However, there is limited research on its role in neurological diseases. Data showed that transgenic mice with *Ptprz1* exhibit symptoms of neurological disorders, including memory decline,^52^ indicating that *Ptprz1* may be a potential factor in neurological diseases. Our data support and validate these findings. After RE-CI, the expression of *Ptprz1* showed an increasing trend (Figure 5G). *Ptprz1* is mainly concentrated in the InN3 subpopulation, characterized by high expression of memory-related molecules such as *Synpr*, *Ptprz1*, and *Grik1* (Figure 4A). Immunofluorescence staining confirmed the presence of *Ptprz1* in slices (Figure 5G), and InN3 plays a significant role in vesicle-mediated synaptic transport and protein localization (Figure S20A). Our results also revealed increased expression of *Ncam2* in the RE-CI group (Figure S37), which was highly enriched in the ExN1 subpopulation (Table S7). *Ncam2* is involved in cytoskeletal organization and synaptic plasticity, playing a role in the development of axons and dendrites.^73^ Synaptic plasticity is the foundation of long-term potentiation and is associated with processes underlying cognitive impairment, suggesting a role for ExN1 in cognitive impairment. The expression of *Ptpro*, which participates in forming excitatory synapses as a synaptic adhesion molecule, increased in the InN6 subpopulation after RE-CI (Figure S37). *Ptpro* is a synaptic cell adhesion molecule and a promoter of synaptic formation,^74^ and there is evidence of its involvement in memory and learning.^75^ GO enrichment analysis of InN6 also revealed terms related to synaptic organization (Figure S23A), and the InN6 subpopulation plays a role in cognitive impairment associated with DRE. The fluorescence intensity of *Kcnip4* in the ExN1 subpopulation increased after treatment (Figure S37). As a member of the potassium channel family, downregulation of *Kcnip4* has been reported to impair neuronal differentiation and function.^76^ Reviewing the analysis of apoptosis-related molecules, *Bax*, we observed that ExN1 inhibits apoptosis, which is consistent with the findings here. The increase in *Kcnip4* expression may enhance certain functions of ExN1, which warrants further investigation. These histopathological validations confirm our snRNA-seq results (Figure 4A). In summary, our results provide reasonable support for the authenticity of the snRNA-seq data and demonstrate a strong enrichment of molecules related to cognitive impairment in the samples, suggesting a correlation between DRE and cognitive impairment in certain aspects.

### Pseudo-temporal analysis of the mouse hippocampal neuron subpopulation

**Figure 6:**
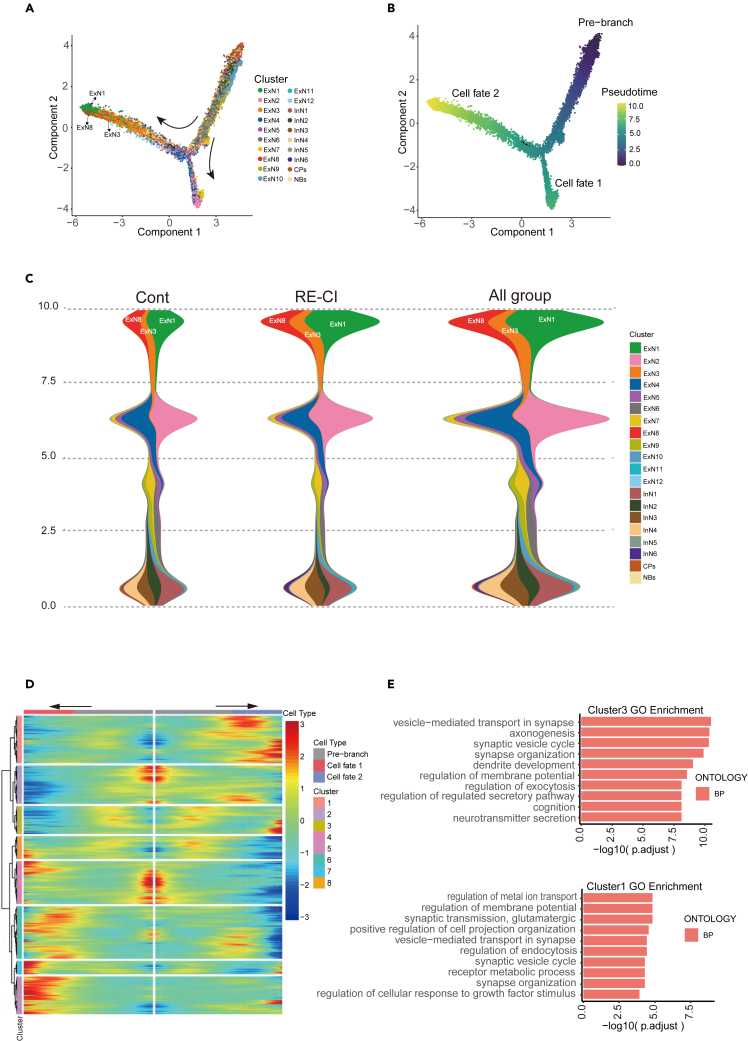
Pseudo-temporal analysis of the mouse hippocampal neuron subpopulation (A) Coloring maps by cell type through pseudo-time series analysis. Different colors indicate different cell types. Arrows indicate the direction of the differentiation trajectory. (B) A diagram of all cells in quasi-sequential order. The order of time indicates the order of the false time of differentiation. (C) Density map of neuronal subpopulations over time of differentiation. The vertical coordinate from bottom to top is the pseudo-time series, the horizontal coordinate is the proportion of cell types at different time points, and various colors represent different cell types. (D) The heatmap shows the pseudo-temporal expression profiles of the first N DEGs in the neuronal subpopulation, which are divided into 8 clusters. (E) GO enrichment of Cluster1 and Cluste3 with pseudo-temporal variation.

Next, we conducted pseudo-time analysis to investigate the distribution of different neuronal subpopulations in the progression of epileptonegesis, and we found that ExN1, 3, and 8 were predominantly distributed in the Cell fate clade 2 (Figures 6A and 6B). Based on the progression of epileptonegesis analysis of individual neuronal subpopulations, we found that ExN1, ExN3, and ExN8 were located at one end of the progression of epileptonegesis, and the changes in ExN1, ExN3, and ExN8 were most significant compared to the control group (Figure 6C). According to the analysis of differentiation path direction, NBs and CPs were identified as potential stem cells of neuronal subtypes, mainly located at the beginning of differentiation (Figure 6C). The distribution of 20 neuronal subpopulations in the control and RE-CI groups along the inverse temporal trajectory was analyzed (Figure S38), revealing that the trajectory of specific subpopulations changed after RE-CI, including ExN1, 3, 8, 10, 11, InN1, 6, and CPs (Figure 6C).

We also performed clustering analysis to identify stage-specific gene expression patterns along pseudotime, resulting in eight distinct clusters of expression profiles (Figure 6D) and their corresponding gene sets (Table S8). GO enrichment analysis was conducted for each cluster (Figure S39). We discovered that clusters 1 and 3, representing ExN1, 3, and 8 subpopulations, were enriched in terms such as "synaptic transmission, glutamatergic," "synapse organization," "cognition," and "axonogenesis" pathways (Figure 6E). These findings further support the selection of memory-related neuronal subpopulations (ExN1, 3, 8) as reasonable candidates.

### Analysis of intercellular communication and regulatory networks in hippocampal neurons of drug-resistant epileptic cognitive impairment mice

**Figure 7:**
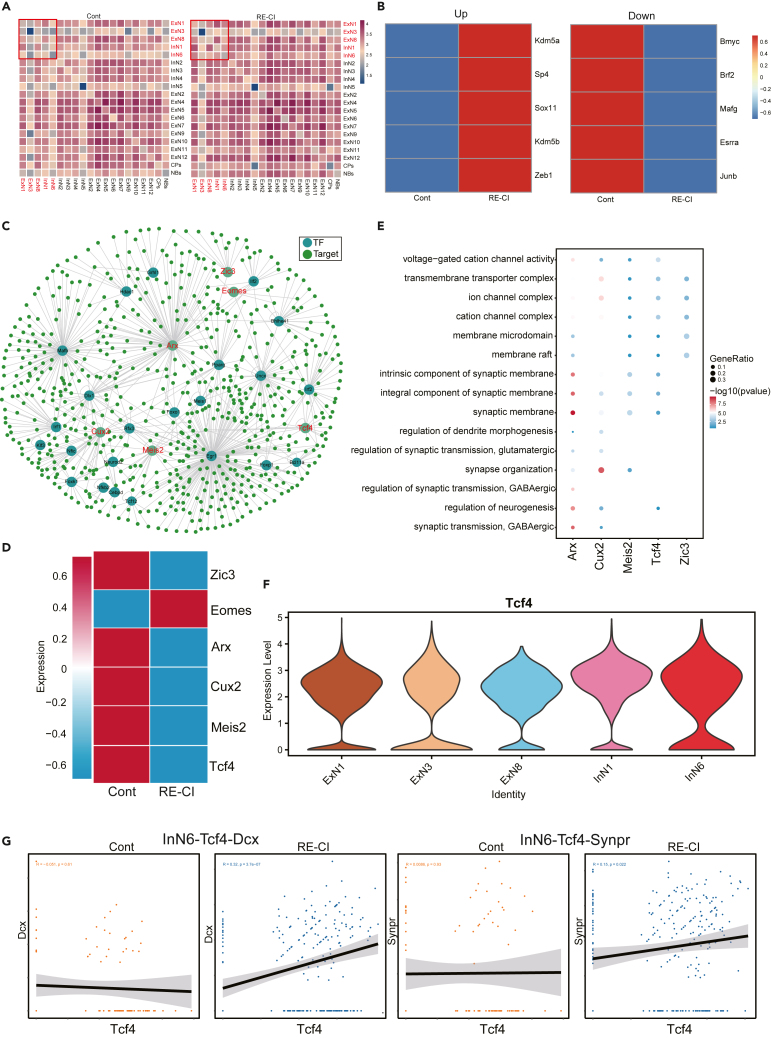
Analysis of intercellular communication and regulatory networks in mouse hippocampal neurons (A) Heatmap showing the total number of ligand interactions between Control and RE-CI hippocampal neuron subpopulations obtained using CellPhoneDB. (B) Heat maps showing the Top5 transcription factors up-regulated and down-regulated after RE-CI. (C) Transcription factor regulatory network map with significant differences between RE-CI and Cont. The six genes in red are memory-related regulators (TF). (D) Heatmap showed the expression of 6 transcription factors (TF) associated with memory in Control and RE-CI. (E) GO enrichment of the target genes of the 5 TFs in hippocampal neurons. (F) Violin diagram showing the expression of Tcf4 in ExN1, 3, 8 and InN1, 6 subpopulations. (G) Correlation analysis between transcription factor Tcf4 and genes related to memory impairment in InN6 subpopulation.

To gain further insights into the interactions among hippocampal neurons in RE-CI mice, we conducted a cell-to-cell communication network analysis using CellphoneDB. Surprisingly, our study revealed that the overall cell communication patterns between RE-CI and control mice were similar (Figure S40A). However, the heatmap demonstrated an increased number of interactions between hippocampal neurons in the RE-CI mice (Figure 7A), suggesting the connections between hippocampal neuron subpopulations increase after cognitive impairment in drug-resistant epileptic mice.

Furthermore, we performed a regulatory network analysis of hippocampal neurons in the control and RE-CI mice, identifying 311 transcription factors (Table S9). By analyzing the top 5 transcription factors from the subpopulation of neurons in RE-CI, we observed an increase in the expression of transcription factor *Sox11*, which regulated the gene *Dcx* associated with cognitive impairment in the InN1 subpopulation, as well as the gene *Lrp1b* related to cognitive impairment in the ExN1 and ExN3 subpopulations (Figure 7B). Additionally, the expression of transcription factor *Sp4* was found to increase, which regulated the gene *Ablim3* associated with cognitive impairment in the InN1 subpopulation. Further, the expression of transcription factor *Esrra* was decreased, which regulated the gene *Kcnip4* associated with cognitive impairment in the ExN1 and ExN3 subpopulations. These findings suggest that the ExN1, ExN3, and InN1 subpopulations are associated with cognitive impairment.

Next, we examined the top 75 transcription factors of control and RE-CI mouse neuronal subpopulations (Figure S40B). we identified 29 transcription factors with expression differences (Figure 7C), out of which six were related to memory. These include *Tcf4*, *Eomes*, *Arx*, *Cux2*, *Zic3*, and *Meis2*. Furthermore, we observed that compared to control mice, RE-CI mice exhibited increased expression of Eomes in hippocampal neuron subpopulations, while the remaining five transcription factors showed decreased expression (Figure 7D). Pathway enrichment analysis of *Arx*, *Cux2*, *Zic3*, *Tcf4*, and *Meis2* revealed their involvement in synaptic-related pathways such as synaptic membrane, postsynaptic membrane, and synaptic transmission (Figure 7E). This suggests that RE-CI may be associated with information transmission between hippocampal synapses. To further investigate the association between cognitive impairment and the ExN1, ExN3, ExN8, InN1, and InN6 subpopulations, we analyzed the specific expression of these six memory-related transcripts in these subpopulations. Surprisingly, *Tcf4* exhibited high expression in the ExN1, ExN3, ExN8, and InN1, ExN6 subpopulations (Figure 7F). Further analysis was conducted to determine whether *Tcf4* regulates the upregulated genes associated with cognitive impairment in these subpopulations (Figure S41). Among them, transcription factor *Tcf4* showed the most significant correlation with *Dcx* and *Synpr* genes in the InN6 subpopulation (Figure 7G), suggesting that InN6 plays a crucial role in regulating cognitive impairment from another perspective.

### Identification of hippocampal neuron subpopulations in mice

**Figure 8:**
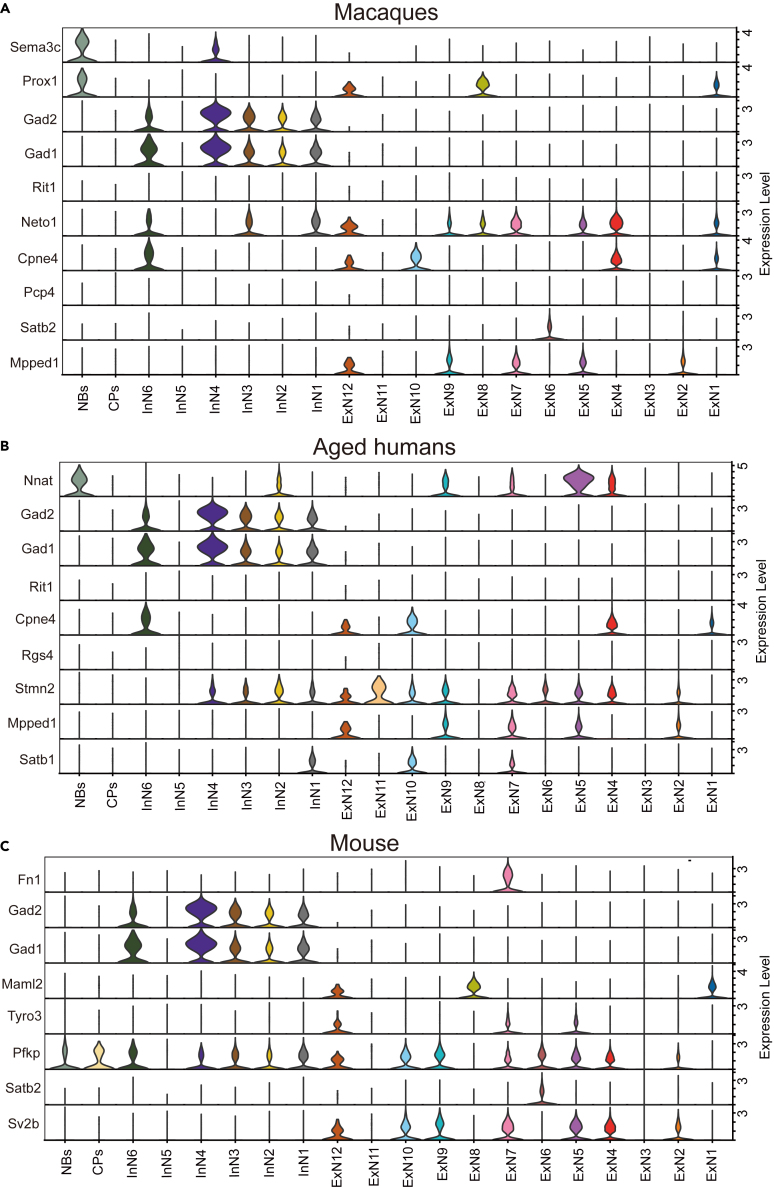
Validation of the hippocampal neuron cell population marker The fiddlegram showed the expression of marker genes from hippocampal CA1, CA2, CA3, Granule cell, ImmN, and GABAergic described in Wei Wang et al. (2022) in macaques (A) and elderly (B) in our dataset. (C) The fiddle-diagram showed the expression of marker genes from mouse hippocampal CA1, CA2, CA3, Granule cell, GABAergic, and Sub (described in Cembrowski et al., 2018; Cembrowski et al., 2016) in our dataset.

We further improved the neuronal resolution to identify 20 neuronal subpopulations (Figure 3D) and conducted GO pathway enrichment analysis to identify five subpopulations potentially associated with cognitive impairment preliminarily. To further validate our recognition of neuron subpopulations, we verified the marker genes well annotated in the hippocampus using our dataset. Marker genes specific to the DG and CA regions of the hippocampus in macaques and elderly individuals from Wei Wang et al., as well as in mice from Cembrowski et al., were examined in our data (Table S5). We observed the expression of known marker genes for the CA2 and CA3 regions in macaques, elderly individuals, and mice within the InN6 subpopulation (Figures 8A–8C).

On the behavioral side, the CA3 region plays a vital role in the rapid formation of episodic memory, which involves connecting spatial and sensory information.^77^ There is also evidence indicating the significance of the CA2 region in various memory processes.^78^ Our analysis results align with these findings, and our GO analysis indicates that InN6 is associated with synapse-related pathways, potentially linked to memory.

In conclusion, we propose that changes in synaptic plasticity of hippocampal neurons in drug-resistant epileptic cognitive impairment mice may be attributed to the up-regulation of synapse-related pathways in the ExN1, 3, 8, and InN1, 6 subpopulations. These pathways include neuron-to-neuron synapse, postsynaptic density, multicellular organismal signaling, transmission of nerve impulse, regulation of membrane potential, vesicle-mediated transport in synapse, and memory. Furthermore, genes associated with cognitive impairment in the ExN1, 3, 8, and InN1, 6 subpopulations, such as *Ptpro*, *Lrp1b*, *Calb1*, *Ncam2*, *Kcnip4*, and *Synpr*, were found to be up-regulated in the RE-CI group (Graphical abstract and Tables S6 and S7).

## Discussion

In this study, we conducted behavioral experiments such as EPM, OFT, TST, FST, NORT, and MWM to examine anxiety, depression, and decreased cognitive abilities in epileptic mice. Spatial navigation and spatial memory, which are primarily associated with the hippocampus, were found to be affected.^79^ MRI scans of drug-resistant epileptic mice with cognitive impairment revealed hippocampal atrophy. Additional research demonstrated that rats with chronic temporal lobe epilepsy induced by pilocarpine exhibited damage to spatial memory and the hippocampal structure.^80^ Transmission electron microscopy analysis of hippocampal neurons in RE-CI mice showed a decrease in synapses and postsynaptic density, which aligns with the findings on the dense region related to learning and memory (PSD) in the hippocampus.^17^ Therefore, we speculate that hippocampal synaptic plasticity is closely linked to drug-resistant epileptic cognitive impairment.

To investigate the heterogeneity of cell composition, communication, molecular expression, and function in the hippocampal region of RE-CI mice, we employed the snRNA-seq technique. This study represents the first attempt to explore the role of neurons in epileptic cognitive impairment using snRNA-seq. Through unsupervised clustering, we identified nine categories of hippocampal cells and twenty subcategories of neuronal cells (ExN1-12, InN1-6, CPs, and NBs). To determine the function of each subpopulation, we divided the study into three categories: screening molecules associated with cognitive impairment, conducting GO KEGG enrichment analysis of synaptic pathways, and performing module gene cluster analysis. Based on our preliminary results, we focused on five subpopulations (ExN1, ExN3, ExN8, InN1, InN6) to analyze the impact of this heterogeneity on cognitive impairment. Figure 5B indicates that ExN1 and ExN3 share similar functions and are highly enriched in Module 5. The differential expression of *Kcnip4* and *Ncam2* molecules deserves our consideration of their functions. However, it is worth noting that *Kcnip4* can also induce excitotoxic neuronal death through the massive release of glutamate in ischemic stroke. Overexpression of *Kcnip4* alone can play a neuroprotective role against excitotoxic neuronal death,^81^ and *Ncam2* is known to be involved in neurogenesis and regulation of synaptic plasticity.^82^ In the study of other neurodegenerative diseases, such as Alzheimer’s disease, a decline in *Ncam2* has been identified as one of the causes of synaptic loss.^83^ Fluorescence results also showed increased expression of *Kcnip4* and *Ncam2* in the RE-CI group. Therefore, our findings suggest a strong correlation between ExN1 and ExN3 and cognitive impairment.

ExN8 exhibits high expression of *Lrp1b* (low-density lipoprotein receptor-associated protein 1b), which is primarily studied in tumors. However, in neurology, reports are suggesting that *Lrp1b* can interact with apoptotic neurons to regulate microglia in phagocytosing neurons.^84^ It can also restrict the occurrence of cognitive impairment by influencing the reduction of neuron numbers, leading to partial functional loss. In summary, ExN8 seems to play a role in promoting cognitive impairment.

*Calb1* (calbindin-1) is highly enriched in MF (mossy fibers) and can impact memory function by modulating synaptic plasticity.^39^ The finding of a high concentration of *Calb1* in MF warrants further investigation as it represents a potential future research direction. Notably, the decrease in *Calb1* fluorescence intensity is a novel finding observed in Figure 5G. In the RE-CI group, *Calb1* expression decreased in other neuronal subpopulations except ExN8. MF is a central hub connecting DG-CA3 signal transmission and is essential for memory transmission,^85^ indicating the importance of neuronal subpopulations other than ExN8 in the memory pathway DG-CA3-CA1. Targeting *Calb1* to modulate pathway transmission may result in cognitive impairment. However, previous research has shown that only ExN8 expression significantly increased in the RE-CI group, indicating that other neuronal subpopulations may possess protective stress mechanisms. At the same time, ExN8 contributes to the mechanism of promoting cognitive impairment.

Synaptic enhancement plays a vital role in memory formation, and *Grik1* (glutamate receptor) in InN1, InN3, and InN4 is vital in the biological process of synaptic enhancement. Differential expression of *Grik1* has been studied in conditions such as developmental delay,^86^ ADHD,^87^ and schizophrenia.^88^ The glutamate ion receptor, kainate 1 (*Grik1*), encoded by the *Grik1* gene, is highly expressed in brain regions responsible for learning and memory, and its expression is reduced in patients with ADHD.^87^ Our results suggest that a decrease in *Grik1* may contribute to cognitive impairment. However, the increase observed in the InN1, InN3, and InN4 subpopulations suggests that these subpopulations may possess protective mechanisms within individuals themselves. The implications of these molecules in other diseases have an intuitive impact on studying cognitive impairment in DRE.

It is important to note that our identification of the functional properties of the 20 subpopulations is only the first step based on differentially expressed gene (DEG) analysis. GO and KEGG databases also indicate that these subpopulations are functionally enriched in synaptic composition or regulation, supporting the necessity of synaptic plasticity for learning and memory.^89^ We categorized functional neuronal subpopulations based on gene clusters, each forming a module. Per our previous understanding of functional attributes, we found that some neuronal subpopulations exhibit similar functions. Therefore, we plan to conduct further validation based on modules (Figures S24A and 5A). Each of the 13 modules corresponds to a category of neuronal subpopulations (Figure 5B). We continue to focus on the functional enrichment of modules belonging to ExN1, ExN3, ExN8, InN1, and InN6 and find that ExN1, ExN3, and ExN8 are predominantly clustered in Module 5. InN1 and InN6 are not concentrated in the same module. The functional activity of ExN1, ExN3, and ExN8 can be represented by Module 5. GO analysis of Module 5 reveals its involvement in synaptic tissue composition or regulation and synaptic activity regulation, further confirming the functional activity of ExN1, ExN3, and ExN8. Similarly, Module 4 corresponding to InN1, and Module 10 corresponding to InN6 were also validated, yielding significant results. Both Module 4 and Module 10 were enriched in the up-regulation of the cAMP signaling pathway (Figures S5F and S5G). cAMP is a mature second messenger for long-term potentiation (LTP) and memory formation/consolidation.^68^ The changes in synaptic strength generated by LTP are widely considered the foundation of memory storage.^90^ Combined with subpopulation analysis using KEGG, ExN1, InN1, and InN6 were found to be enriched in terms related to LTP and LTD (Figures S5A, S5D, and S5E), suggesting that these three subpopulations may influence the development of cognitive impairment by altering synaptic modification through the LTP pathway. However, biological analysis alone is insufficient. We also verified immunofluorescence verification on pathological sections, including *Ptprz1*, *Calb1*, *Ptpro*, *Kcnip4*, *Ncam2*, and other molecules (Figure S37; Table S7). These molecules are closely related to memory production or maintenance in different neurological diseases. Some molecules participate in synaptic tissue composition, regulate synaptic plasticity, and act as essential mediators of signal transmission. Others affect memory production in various neurological diseases, as validated in pathological sections.

Through further analysis of the regulatory network among the 20 neuronal subpopulations, we observed enhanced interaction between the subpopulations in the RE-CI group, along with the altered regulation of transcription factors. Remarkably, the expression of transcription factors *Tcf4*, *Arx*, and *Cux2* was significantly decreased in mice with epileptic cognitive impairment, and GO analysis revealed their enrichment in synapse-related pathways, such as an integral component of synaptic membrane, synaptic membrane, and synapse organization (Figure 7E). Previous studies have demonstrated the critical role of *Tcf4* in neurodevelopment and cognition.^91^ *Arx* primarily affects network excitability through genes associated with synaptic and extracellular matrix pathways.^92^ *Cux2* is specifically expressed in neural tissue,^93^ and *Cux2* knockout mice exhibit reduced synaptic function and deficits in working memory.^94^ Furthermore, our analysis of the correlation between *Tcf4* and *Dcx/Synpr* genes in the InN6 subpopulation provides strong evidence supporting the association between the In6 subpopulation and cognitive impairment.

Wei Wang et al. analyzed hippocampal neurogenesis throughout the life cycle of macaque monkeys.^95^ They utilized GO analysis to study the biological processes and functions of each cell type. They discovered that immature neurons (ImmN) participate in "cell migration" and "axon extension," while mature granular cells (GC) are involved in "neurotransmitter secretion" and "synaptic assembly." This finding inspired us to further analysis. Through reverse temporal analysis and GO functional enrichment of mouse hippocampal neuron subpopulations, we found that clusters 1 and 3, corresponding to ExN1, 3, and 8 subpopulations, were also enriched in neurotransmitter secretion. Additionally, we identified well-annotated marker genes in the hippocampus for validation in our dataset, which aids in the functional annotation of neuronal subpopulations.

In summary, we have established a single-cell transcriptional roadmap of the hippocampus in RE-CI mice. Our molecular expression analysis, GO and KEGG database analysis, and module gene set analysis demonstrated the correlation between ExN1, 3, 8 and InN1, 6 subpopulations. We verified that ExN1, 3, InN1, and 6 have a protective effect on individual cognitive impairment, while ExN8 promotes cognitive impairment.

### Limitations of the study

We recognize that this study still has several limitations. Firstly, our data only pertains to mice with RE-CI, and there is a lack of snRNA-seq reference data for humans with RE-CI. Secondly, the amount of data analyzed in our study is extensive, and we solely focused on researching cognitive impairment in neurons, leaving ample room for mining information regarding other cell types, such as astrocytes and microglia. We hope our snRNA-seq data can provide novel ideas and insights for future research on epileptic cognitive impairment.

## Resource availability

### Lead contact

Further information and requests for resources and reagents should be directed to and will be fulfilled by the lead contact, Bei Liu (liubei206@163.com).

### Materials availability

This study did not generate new unique reagents.

### Data and code availability

• SnRNA-seq data associated with this study have been deposited in the Gene Expression Omnibus (GEO) (https://www.ncbi.nlm.nih.gov/geo/) and will be publicly available at the time of publication with accession number GSE241349.
• This article does not report the original code.
Any additional information required to reanalyze the data reported in this article is available from the lead contact upon request.

## Acknowledgments

We would like to thank Xingeyuan Company for its help in snRNA-seq data analysis, the Laboratory Animal Center of the 10.13039/501100007547Fourth Military Medical University for its support of C57 mice, the 10.13039/501100007547Fourth Military Medical University for its support of electron microscopy and other experimental equipment, and the Imaging Diagnostic Center of Xi'an Yinghe Hospital for its support of nuclear magnetic imaging. We would like to thank the members of the whole research group for all the suggestions and help provided for this article and thank the neurosurgery department of Tangdu Hospital of the Fourth Military Medical University for providing experimental instruments and platforms such as behavior and staining.

## Author contributions

BL and LS designed research. YB, LS, CW, LG, DZ, XQ, and performed research. JM, LW, BL, and LS performed the bioinformatics analysis. QF provided materials. JM, LW, BL, and LS wrote the article.

## Declaration of interests

The authors declare no competing interests.

## STAR★Methods

### Key resources table

**Table:** 

REAGENT or RESOURCE	SOURCE	IDENTIFIER
**Antibodies**		

Anti-PTPRZ1 antibody	Proteintech	Cat# 55125-1-AP; RRID: AB_10858795
Anti-Calb1 antibody	Proteintech	Cat# 14479-1-AP; RRID: AB_2228318
Anti-Kcnip4 antibody	Proteintech	Cat# 60133-1-Ig; RRID: AB_2130271
Anti-Ncam2 antibody	Proteintech	Cat# 13850-1-AP; RRID: AB_2237029
Anti-Ptpro antibody	Proteintech	Cat# 67000-1-Ig; RRID: AB_2882317
Donkey Anti-mouse IgG H&L (Alexa Fluor™ 488)	Abcam	Cat# ab150105; RRID: AB_2732856
Goat Anti-Rabbit IgG H&L (Alexa Fluor™ 594)	Abcam	Cat# ab150080; RRID: AB_2650602

**Chemicals**		

Lamotrigine	GSK	GlaxoSmithkline
Pentylenetetrazole	Aladdin	P103065-5g

**Deposited data**		

Raw snRNA-seq data	This paper	Gene Expression Omnibus (GEO): GSE241349

### Experimental model and study participant details

#### Animals and ethical statement

The experimental animals used in this study were male C57BL/6J mice with a background from the Laboratory Animal Center of the Fourth Military Medical University. All mice were screened and confirmed to be free of specific pathogens, and the test report was attached for reference. The mice were housed in polycarbonate cages, with approximately five mice per cage, and provided with *ad libitum* access to sterile water and food. The animal room was maintained at a room temperature of 22 ± 1°C, humidity of 50%–60%, and followed a 12-h light-dark cycle (lights on at 8:00 a.m.). This study has been approved by the Ethics Committee of the Fourth Military Medical University (Approval Number: IACUC20220555).

### Method details

#### Animals

The mice were purchased at eight weeks of age and allowed to acclimate to the environment in three animal rooms at Tangdu Hospital for one week. The lamotrigine-pentylenetetrazole kindled model was started at nine weeks.

#### Lamotrigine-pentylenetetrazole kindled epilepsy model

In the kindled group, a subtherapeutic dose of lamotrigine (5 mg/kg) was injected 30 min before each pentylenetetrazole injection (40 mg/kg). A subconvulsive dose of pentylenetetrazole was injected every 48 ± 2 h for 35 consecutive days. Following each pen tetrazine injection, the mice were placed in clear plexiglass cages, and any convulsive seizures were recorded. Persistent seizures were terminated immediately by administering Diazepam (10 mg/kg).^96^ The severity of seizures was classified using the modified Racine scale. Successful modeling was achieved when each mouse exhibited grade IV to grade V seizures after three consecutive injections. The control group mice were intraperitoneally injected with an equal volume of normal saline. The modified Racine scale classification is as follows: Grade I: Oral and facial movements; Grade II: Chewing, nodding, and shaking wet dog; Grade III: Forelimb clonus; Grade IV: Lifting and double forelimb clonus; Grade V: Loss of balance after clonus, fall.

#### Open field test (OFT)

The OFT began on the 5th day after the success of the model. The open field device is a box that is open on the top. The parameters are 432mm(D) x 432mm(W) x 305mm(H). We selected 30 mice for the OFT, with 15 mice in the RE group and 15 mice in the control group.The test began with mice placed alone in the center of an open field device and allowed to explore the field for 5 min freely. The central area is a square, 108mm from the wall. A camera tracked the movements of each mouse, and the dwell time and crossing times in and around the surrounding area were calculated by software (EthoVision XT Version 8.0 (Noldus, the Netherlands)).^97^^,^^98^^,^^99^

#### Elevated plus maze (EPM)

The EPM began on the 6th day after the success of the model. The plexiglass device is composed of two opposite "open" arms (50mm(D) × 250mm(W)), two opposite "closed" arms (50mm(D) × 250mm(W)) and a central area (50mm(D) × 50mm(W)). We selected 30 mice for the EPM with 15 mice in the RE group and 15 mice in the control group. The instrument was raised 50cm above the ground. At the beginning of the experiment, the mice were placed alone in the central area, facing the closed arm, and allowed to explore the maze for 5 min freely. A camera tracked the movements of each mouse, and the residence time and crossing times on the open and closed arms were calculated by software (EthoVision XT Version 8.0 (Noldus, the Netherlands)).^97^^,^^98^^,^^99^

#### Novel object recognition test (NORT)

The NORT began on the 7th day after the success of the model, and the NORT and the OFT were conducted in the same arena. We selected 30 mice for the NORT, with 15 mice in the RE group and 15 mice in the control group. On the first day, the animals were placed in the arena with their backs to two identical objects (familiar objects) and allowed to freely explore the environment and objects for 5 min. After an interval of 24 h, the animals were placed in the arena with a novel object and a familiar object (test day), and the animals were allowed to explore these objects for 5 min. The objects used in the test were all wooden objects of different shapes and colors. Memory for familiar objects was measured as the percentage of time spent studying the new object to the total exploration time (i.e., discrimination index). The exclusion criteria were as follows: if the mice did not spend time studying the two objects during training, or did not spend time studying any of the objects during the test, the test data was excluded from the analysis because it could not be confirmed that they spent enough time exploring to learn/discriminate. All behavioral tests were performed and analyzed in a blind manner.^100^

#### Tail suspension test (TST)

The TST began on the 8th day after the success of the model. We selected 30 mice for the TST, with 15 mice in the RE group and 15 mice in the control group. Each mouse’s tail was suspended with tape from a hanging rod 60cm off the ground, and the tape was placed less than 1cm from the tip of the tail. A stopwatch was used to measure the immobility time of each mouse for 6 min, and the last 4 min were analyzed. If the mouse does not move, it is considered immobile.^99^^,^^101^

#### Forced swim test (FST)

The FST is based on previously published reports. The FST began on the 9th day after the success of the model. We selected 30 mice for the FST, with 15 mice in the RE group and 15 mice in the control group. In short, each mouse was swimming freely in a glass cylinder (10 cm × 25 cm) filled with water. The mice were allowed to swim for 6 min, and the resting time of each mouse during the last 4 min was recorded. Then, the data are calculated and analyzed. If the mouse stopped swimming, remained motionless, or displayed a state of desperation and stopped escaping from the cylinder filled with water, it was considered stationary.^99^^,^^101^

#### Morris water maze (MWM)

The MWM began on the 10th day after the success of the model. The water maze consists of a circular tank (120cm in diameter and 50cm in height) with a black inner wall, a digital camera, and a computer with a tracking system. We selected 30 mice for the MWM, with 15 mice in the RE group and 15 mice in the control group. The circular tank is 30cm off the ground on a secure platform and filled with water. An escape platform is located in the center of the northeast quadrant, about 1.5cm above the water. During the training phase, the mice were given 60 s per trial to find the platform. If the mice could not locate the platform within the 60s, it was necessary to induce them to reach the platform artificially, let them rest on the platform for 15s, and then conduct the next test. Each mouse was trained four times a day for four days. After four days of training, the mice were tested for 60s after removing the platform on day 5. the behavior of each mouse during the training and testing phases was recorded by a digital camera, and the calculated data 2-4 was analyzed by software (EthoVision XT Version 8.0 (Noldus, the Netherlands)).^98^^,^^102^^,^^103^

#### Electroencephalogram (EEG) monitoring

EEG recordings were conducted 2 to 3 weeks after successful modeling or during lamotrigine-pentylenetetrazole-induced seizures. One week before the EEG recording, the mice were anesthetized using isoflurane, and their heads were secured in a stereotaxic apparatus. The skin was incised, exposing the positions of the fontanelle and posterior fontanelle. Three holes, approximately 0.1cm in diameter, were drilled on the skull surface, including one as a reference electrode and two as recording electrodes. The reference electrode was positioned on the right forehead (AP:+1.5mm, ML:+0.4mm), while the recording electrodes were placed above the hippocampus on both sides (AP:-1.8mm, ML:±2.3mm). Following implantation, all electrodes were insulated and secured to the skull surface using glass ion cement. Once the cement had solidified, the mice were removed from the stereotaxic apparatus and placed on an electric heating plate until they regained consciousness. EEG recording commenced seven days after recovery. The surviving mice with implanted electrodes were individually placed in recording cages and connected to an electroencephalograph via flexible wires. During the recording period, the mice had *ad libitum* access to food and water. Spontaneous seizures of drug-resistant epileptic MEMORY mice were observed by continuously recording EEG activity (sampled at 300Hz) for 24–72 h. Generalized seizures were defined as repetitive seizure-like spike activity (≥3Hz) lasting for more than 3 s and present in all electrodes.^29^ Convulsive seizures were characterized by high amplitude (> Double background activity) and tonic-clonic spiking activity lasting more than 3 s (>3Hz), which were confirmed by video monitoring. Non-convulsive seizures were identified using the same EEG criteria, but their behavioral seizure activity did not exceed level 2 of the modified Racine scale.

#### Laser speckle contrast imaging (LSCI)

We selected 12 mice for the laser speckle contrast imaging, with 6 mice in the RE-CI group and 6 mice in the control group. After anesthesia, the mice were randomly fixed on a thermostatic electric plate, and the blood flow of the cerebral cortex on both sides was monitored by LSCI in a prone position. The operator knows nothing about group assignments. Cut the skin at the beginning so that the skin and mucous membranes are sufficiently separated to keep the skull fully exposed. The LSCI system focuses on the mouse skull for a clear color image. Saline is injected into the skull to keep it moist. During the monitoring process, the mice were kept under anesthesia to obtain stable breathing and heart rate, and were continuously monitored for 8 to 10 s. Set the area of Interest (ROI), where the ROI value represents the flow in the selected location.^104^^,^^105^^,^^106^

#### Magnetic resonance imaging (MRI) and data analysis

We selected 12 mice for the MRI, with 6 mice in the RE-CI group and 6 mice in the control group. Mice were anesthetized, and their brains were imaged using a 3T animal small MRI Scanner (Bruker MRI GmbH, Germany). Hippocampal tissue was obtained using an MRI scanner with an RF surface and a phased array of mouse brain coils. The brain tissue was scanned and cut into equidistant coronal slices, each 1 mm thick. Finally, MRI images in DICOM format were exported. Hippocampal volumes were obtained from MRI images using ImageJ software. The hippocampus was manually outlined, sections of interest selected, and measurements from consecutive sections merged together by two Neurologists who were blinded to grouping. Volume measurements from each section were normalized to brain volume, yielding a ratio that was independent of the animal’s brain size. The hippocampal volume ratio of RE-CI and Cont mice was compared using T test, and the significance threshold was set at *p* < 0.05.

#### Transmission electron microscope (TEM)

We selected 12 mice for the transmission electron microscope, with 6 mice in the RE-CI group and 6 mice in the control group. The mice were deeply anesthetized and injected with 0.01% PFA through the heart. As previously mentioned, hippocampal tissue was immobilized in 4% glutaraldehyde at 4°C for 16 h. After being fixed in 1% osmium tetroxide for 60 min, the sample was dehydrated and encased in resin using a graded ethanol series. The embedded sample blocks were trimmed and cut using an ultramicrotome, after which the slices were placed on a 200-cell grid coated with PVA ester and imaged under a JEM 1400 electron microscope (HITACHI, Japan).

#### Immunofluorescence staining (IF)

We selected 12 mice for the immunofluorescence staining, with 6 mice in the RE-CI group and 6 mice in the control group. The mice were anesthetized with pre-cooled PBS solution for cardiac perfusion. After the liver was completely white, the mice were injected with pre-cooled 4% paraformaldehyde until the whole body was stiff. Immediately after perfusion, the brain was taken and stored in a centrifuge tube containing frozen 4% paraformaldehyde solution at 4°C overnight, and then gradient dehydrated with 20% and 30% sucrose solution. After dehydration, 25μm thick frozen slices were prepared using the frozen microtome.

The brain samples of RE-CI and Control mice were fixed with 4% paraformaldehyde. The brain slices (25 μm) were incubated overnight at 4°C with the following primary antibody: anti-Ptprz1 (1:250, Proteintech, 55125-1-AP), anti-Calb1 (1:250, Proteintech, 14479-1-AP), anti-Kcnip4 (1:250, Proteintech, 60133-1-Ig), anti-Ncam2 (1:250, Proteintech, 13850-1-AP), anti-Ptpro (1:250, Proteintech, 67000-1-Ig). Samples were then incubated with the secondary antibodies of Alexa Fluor 488/594 Donkey anti-Mouse/Rabbit IgG (1:400, Invitrogen) at room temperature for 4 h. The nucleus was restained with DAPI. Finally, images were obtained by laser confocal image acquisition microscope (Nikon, A1, Tokyo, Japan).

#### MWM and NORT screening RE-CI mice

After completing the NORT and MWM, we selected cognitively impaired mice from the RE group based on the time the mice spent exploring familiar objects and the number of times the mice crossed the platform on the fifth day. The less time the mice spent exploring familiar objects and the fewer times they crossed the platform, the greater the degree of cognitive impairment.

#### Nuclei isolation sorting from hippocampal tissue

Hippocampal tissues were harvested from mice and were washed in pre-cooled PBSE (PBS buffer containing 2 mM EGTA). Nuclei isolation was performed using GEXSCOPE Nucleus Separation Solution (Singleron Biotechnologies, Nanjing, China) refer to the manufacturer’s product manual. Isolated nuclei were resuspended in PBSE to 106 nuclei per 400μL, filtered through a 40μm cell strainer, and counted with Trypan blue. Nuclei enriched in PBSE were stained with DAPI (1:1,000) (TermoFisher Scientific, D1306). Nuclei were defined as DAPI-positive singlets.

#### Single nucleus RNA-sequencing library preparation

We constructed six single nucleus RNA sequencing libraries using hippocampal tissue from 3 normal mice and 3 RE-CI mice, and one sample was used to construct a single nucleus RNA sequencing library. The concentration of single nucleus suspension was adjusted to 3-4 × 105 nuclei/mL in PBS. Single nucleus suspension was then loaded onto a microfluidic chip (GEXSCOPE Single NucleusRNA-seq Kit, Singleron Biotechnologies), and snRNA-seq libraries were constructed according to the manufacturer’s instructions (Singleron Biotechnologies). The resulting snRNA-seq libraries were sequenced on an Illumina novaseq 6000 instrument with 150 bp paired end reads.

#### Primary analysis of raw read data (snRNA-seq)

Raw reads were processed to generate gene expression profiles using CeleScope v1.5.2 (Singleton Biotechnologies) with default parameters. Briefly, Barcodes and UMIs were extracted from R1 reads and corrected. Adapter sequences and poly A tails were trimmed from R2 reads and the trimmed R2 reads were aligned against the GRCm38 (mm10) transcriptome using STAR(v2.6.1b). Uniquely mapped reads were then assigned to genes with FeatureCounts(v2.0.1). Successfully Assigned Reads with the same cell barcode, UMI, and gene were grouped to generate the gene expression matrix for further analysis.

#### Quality control, dimension-reduction, and clustering (scanpy)

Scanpy v1.8.1 was used for quality control, dimensionality reduction, and clustering under Python 3.7. For each sample dataset, we filtered the expression matrix by the following criteria: 1) cells with a gene count less than 200 or with a top 2% gene count were excluded; 2) cells with a top 2% UMI count were excluded; 3) cells with mitochondrial content >10% were excluded; 4) genes expressed in less than five cells were excluded (Figure S42). After filtering, 61215 cells were retained for the downstream analyses, with on average 1293.07 genes and 2468.09 UMIs per cell. The raw count matrix was normalized by total counts per cell and logarithmically transformed into a normalized data matrix. The top 2000 variable genes were selected by setting flavor = ‘seurat’. Principal Component Analysis (PCA) was performed on the scaled variable gene matrix, and a top 20 principle components were used for clustering and dimensional reduction. Cells were separated into 23 clusters by using Louvain algorithm and setting resolution parameter at 1.5. These 23 clusters are the results of unsupervised clustering, and we subsequently merged the unsupervised results into nine major subpopulations, where the neurons were subdivided into 20 subpopulations with a resolution improvement of 1. Cell clusters were visualized by using Uniform Manifold Approximation and Projection (UMAP).^107^

#### Batch effect removal

Harmony: The batch effect between samples was removed by Harmony v1.0 using the top 20 principal components from PCA.^108^

#### Statistics and repeatability of single-nucleus RNA sequencing data

Cell distribution comparisons between two groups were performed using unpaired two-tailed Wilcoxon rank-sum tests. Comparisons of gene expression or gene signature between two groups of cells were performed using unpaired two-tailed Student’s t test. Comparisons of cell distribution of paired group1 and group2 as well as RE-CI and Cont were performed using paired two-tailed Wilcoxon rank-sum tests. All statistical analyses and presentation were performed using R. Statistical tests used in figures were shown in figure legends and statistical significance was set at *p* < 0.05. Exact value of n was revealed in the figures and figure legends and what n represents was demonstrated in the figure legends.

#### Differentially expressed genes (DEGs) analysis (scanty)

To identify differentially expressed genes (DEGs), we used the scanpy.tl.rank_genes_groups() function based on the Wilcoxon rank-sum test with default parameters, and selected the genes expressed in more than 10% of the cells in either of the compared groups of cells and with an average log(Fold Change) value greater than one as DEGs. Adjusted *p* value was calculated by benjamini-hochberg correction, and the value 0.05 was used as the criterion to evaluate the statistical significance.

#### Pathway enrichment analysis

To investigate the potential functions of DEGs, Gene Ontology (GO) and Kyoto Encyclopedia of Genes and Genomes (KEGG) analysis were used with the “clusterProfiler” R package v 3.16.1.^109^ Pathways with p_adj value less than 0.05 were considered as significantly enriched. Selected significant pathways were plotted as bar plots. GSEA was performed on neuron-to-neuron synapse and postsynaptic density between control and RE-CI in neuron cluster. For GSVA pathway enrichment analysis, the average gene expression of each cell type was used as input data.^110^ Gene Ontology gene sets including molecular function (MF), biological process (BP), and cellular component (CC) categories were used as reference.

#### Cell-type recognition with Cell-ID

Cell-ID is multivariate approach that extracts gene signatures for each cell and perform cell identity recognition using hypergeometric tests(HGT).^111^ Dimensionality reduction was performed on normalized gene expression matrix through multiple correspondence analysis, where cells and genes were projected in the same low dimensional space. Then a gene ranking was calculated for each cell to obtain most featured gene sets of that cell. HGT was performed on these gene sets against brain reference from SynEcoSys database, which contains all cell-type’s featured genes. Identity of each cell was determined as the cell-type has the minimal HGT *p* value. For cluster annotation, Frequency of each cell-type was calculated in each cluster, and cell-type with highest frequency was chosen as cluster’s identity. The cell type identification of each cluster was determined according to the expression of canonical markers from the reference database SynEcoSys (Singleton Biotechnology). SynEcoSys contains collections of canonical cell type markers for single-cell seq data, from CellMakerDB, PanglaoDB and recently published literatures. The canonical markers and their corresponding cell types were listed in Tables S1 and S3.

#### Subtyping of significant cell types

To obtain a high-resolution map of neurons, cells from the specific cluster were extracted and reclustered for more detailed analysis following the same procedures described above and by setting the clustering resolution as 1.

#### Filtering cell doublets

Cell doublets were estimated based on the expression pattern of canonical cell markers. Any clusters enriched with multiple cell type-specific markers were excluded for downstream analysis.

#### Cell-cell interaction analysis: CellPhoneDB

Cell-cell interaction (CCI) were predicted based on known ligand–receptor pairs between two cell types/subtypes by Cellphone DB (v2.1.0)^112^ version. The permutation number for calculating the null distribution of average ligand-receptor pair expression in randomized cell identities was set to 1000. Individual ligand or receptor expression was thresholded by a cutoff based on the average log gene expression distribution for all genes across each cell type. Predicted interaction pairs with *p* value < 0.05 and of average log expression >0.1 were considered as significant and visualized by heatmap_plot and dot_plot in CellphoneDB.

#### Cell differentiation potential evaluation: CytoTRACE

CytoTRACE v0.3.3 (ref) (a computational method that predicts the differentiation state of cells from single-cell RNA-sequencing data using gene counts and expression) was used to indicate the differentiation potential of cell subpopulations.^113^

#### Pseudotime trajectory analysis: monocle2

Cell differentiation trajectory of monocyte subtypes was reconstructed with the Monocle2 v 2.10.0.^114^ For constructing the trajectory, the top 2000 highly variable genes were selected by Seurat(v3.1.2) FindVairableFeatures(), and dimension-reduction was performed by DDRTree(). The trajectory was visualized by the plot_cell_trajectory() function in Monocle2.

#### Functional gene module analysis (hotspot)

Hotspot was used to identify functional gene modules for neurons, which illustrate heterogeneity within 13 subpopulations.^115^ Briefly, we used the ‘danb’ model and selected the top 500 genes with highest autocorrelation *Z* score for module identification. Modules were then identified using the create_modules function, with min_gene_threshold = 15 and fdr_threshold = 0.05. Module scores were calculated by using the calculate_module_scores function.

#### Jaccard similarity analysis

The Jaccard similarity coefficient was calculated for comparing transcriptional similarity between two cell types using their signature genes. We evaluated transcriptional similarity between 13^116^ hotspot modules of Neurons and signatures of 20 subclusters of Neurons by calculating Jaccard similarity coefficients using the top 50 marker genes.

#### UCell gene set scoring

Gene set scoring was performed using the R package UCell v 1.1.0.^117^ UCell scores are based on the Mann-Whitney U statistic by ranking query genes in order of their expression levels in individual cells. Because UCell is a rank-based scoring method, it is suitable for large datasets containing multiple samples and batches.

#### Transcription factor regulatory network analysis (pySCENIC)

Transcription factor network was constructed by pyscenic (v0.11.0) using scRNA expression matrix and transcription factors in AnimalTFDB. First, GRNBoost2 predicted a regulatory network based on the co-expression of regulators and targets. CisTarget was then applied to exclude indirect targets and to search transcription factor binding motifs. After that, AUCell was used for regular activity quantification for every cell. Cluster-specific TF regulons were identified according to Regulon Specificity Scores (RSS) and the activity of these TF regulons were visualized in heatmaps.^118^

### Quantification and statistical analysis

For snRNA-seq data, Wilcoxon rank-sum tests were used to detect differential genes. For all other data, all experiments were replicated at least 3 times. Statistical analysis and plotting was performed using GraphPad Prism 8.0 and ImageJ software, excluding single-nucleus RNA sequencing data. Data were tested for normal distribution using the Shapiro-Wilk test to determine the use of parametric or nonparametric tests. Two tailed *p* values are reported for t tests. The unmatched bilateral t-test was utilized to determine the statistical significance, and *p* < 0.05 was considered statistically significant.

## Supplemental information

Document S1. Figures S1–S42, Tables S1–S3, and S5–S7

Table S4. Total neuronal differential transcripts between the control group and RE-CI, related to Figure 3

Table S8. Gene sets of eight different expression profiles, related to Figure 6

Table S9. Regulatory genes and their targets identified in neurons, related to Figure 7
