# Supplementary material for: Single-nucleus RNA sequencing-based construction of a hippocampal neuron atlas in mice with epileptic cognitive impairment
Source: iScience. 2024 Sep 28;27(10):111065. doi: 10.1016/j.isci.2024.111065 (PMC11615225; doi:10.1016/j.isci.2024.111065)
Supplement: Document S1. Figures S1–S42, Tables S1–S3, and S5–S7 [file mmc1.pdf]

iScience, Volume *27*

Supplemental information

Single-nucleus RNA sequencing-based construction of a hippocampal neuron atlas in mice with epileptic cognitive impairment

Jia-Qi Ma, Lu Wang, Yue Zhang, Yong-Qian Bian, Xiao-Peng Qu, Li-Jia Song, Chao Wang, Li Gao, Qi-Xing Fang, De-Chang Zhao, Liang-Liang Shen, and Bei Liu

Figure S1

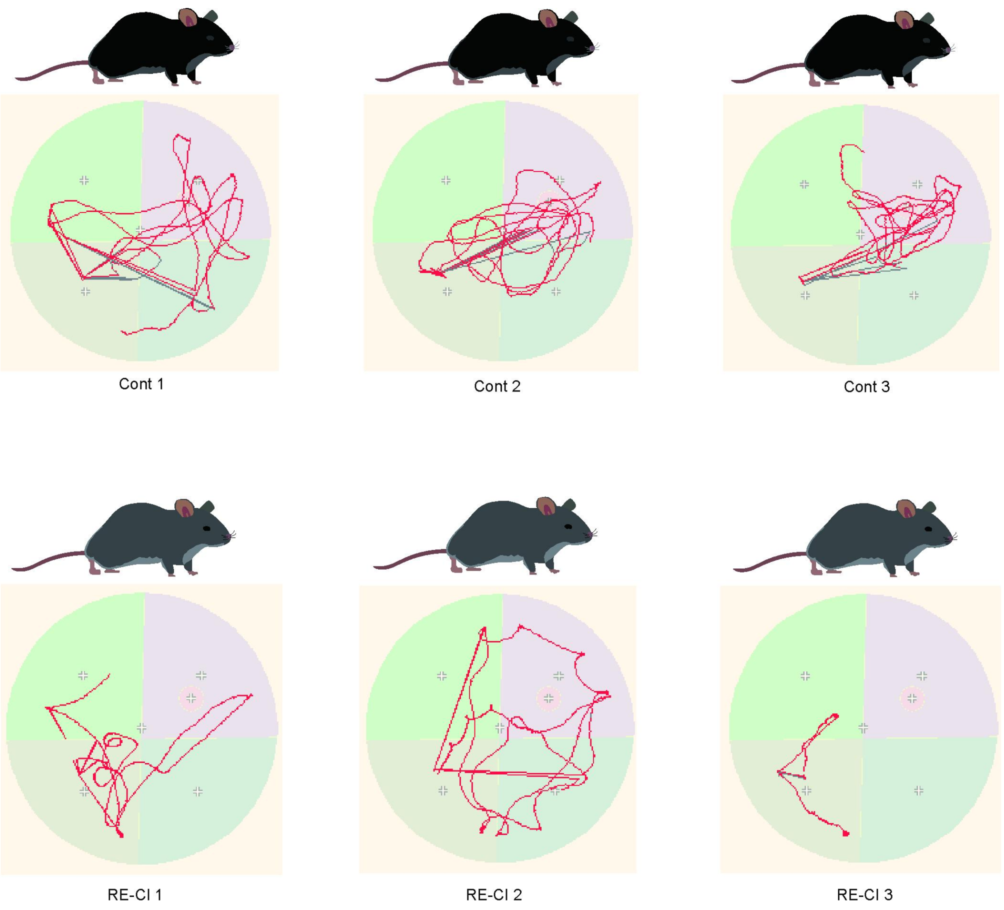

Figure S1. Representative trace of the Morris water maze of Control and RE-CI mice for sequencing (related to Figure 3)

Figure S2

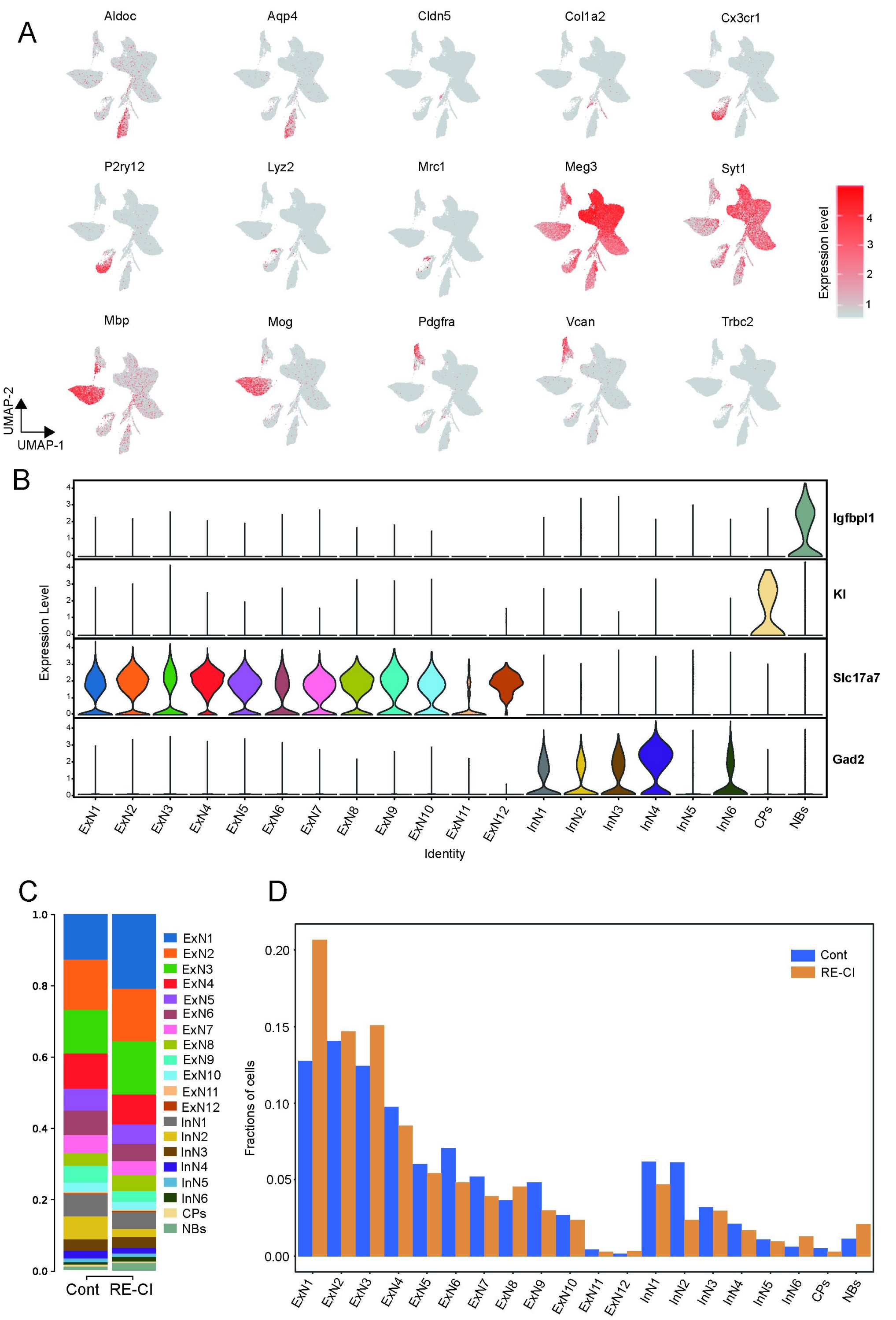

Figure S2. (related to Figure 3)
(A) The UMAP map showed the expression profiles of specific cell type marker genes for corresponding cell types in the hippocampus of mice.
(B) Violin plots of the expression values of neuromotor, choroid, excitatory, and inhibitory neuronal markers.
(C) Frequency distribution of 20 subgroups of neurons in Control and RE-CI mice.
(D) Analysis of the cell number ratio of each neuron subpopulation between the Control and RE-CI mice. The horizontal axis represents the neuron subpopulation, the vertical axis represents the average proportion of cell types to the total number of cells in the sample, and the color represents the sample group.

Figure S3

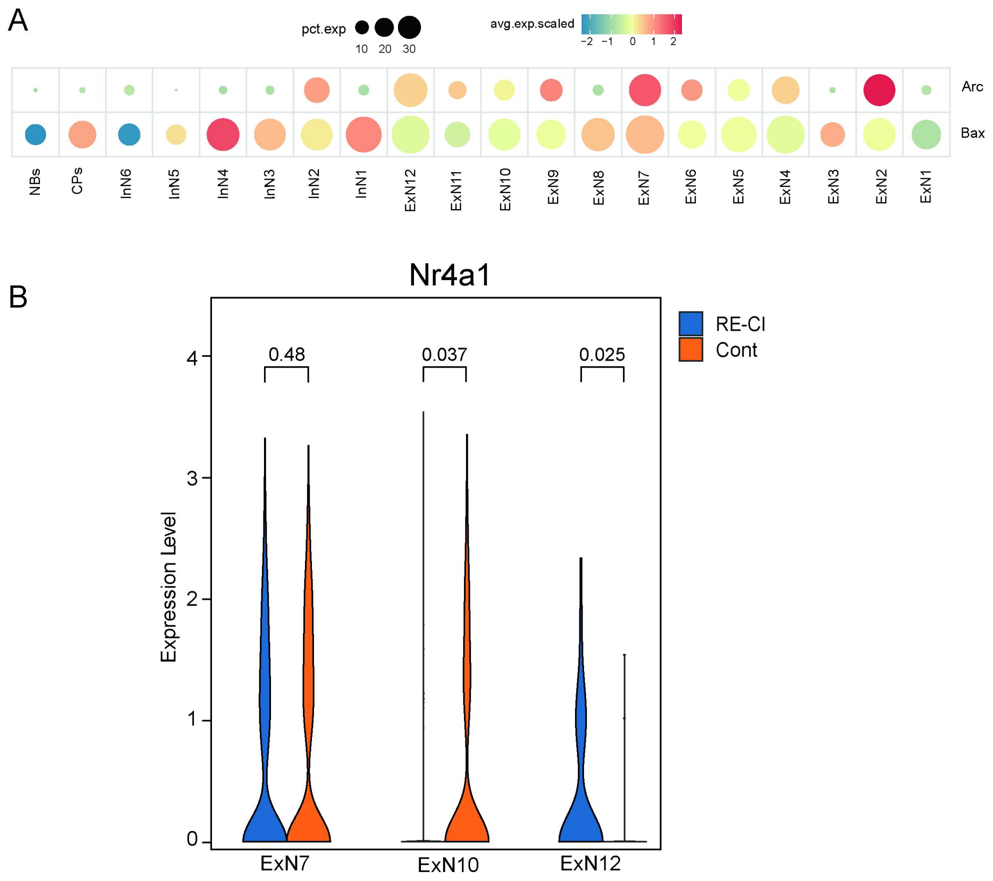

Figure S3. Potential factors of neuron subclass cell number alterations, which were associated with apoptosis and activation (related to Figure 3)

(A) Bubble map of apoptotic Maker (Arc, Bax) expression after comparison between neuron subgroups. The color depth of the bubble represents the P-value, and the size represents the number of differential genes.

(B) The violin diagram showed the expression of activating molecule Nr4a1 in neurons ExN7, ExN10, and ExN12 ($P < 0.05$).

Figure S4

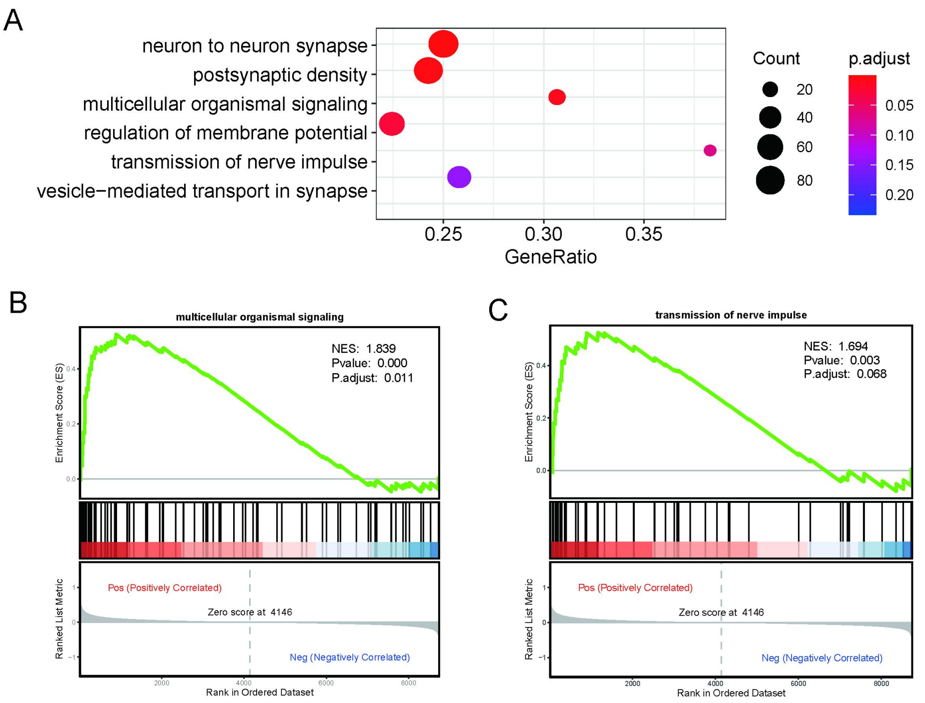

Figure S4. GSEA analysis (related to Figure 4)

(A) 6 GO pathways with significant differences after comparison between Control and RE-CI groups were selected for GSEA analysis. The color of the circles represented the P-value size, and the size of the circles represented the number of different genes.

(B-C) GSEA analysis of the multicellular organismal signaling and transmission of nerve impulse pathways. The top line represents the change in ES value, and the ES value is the accumulation of statistics of each gene in the gene set. The highest or lowest point represents the ES value of the gene set. In the middle are hits, which represent the sequence position of the genes in the gene set. Below is a display of rank values. GSEA can compare the enrichment of a list of selected gene sets (for example, all genes in a pathway) with the degree of difference of all genes in the two groups (RE-CI vs Control). If the curve is enriched at the top (NES>0, P≤ 0.05), then the gene set was significantly enriched in the RE-CI group, otherwise it was enriched in the Control group.

Figure S5

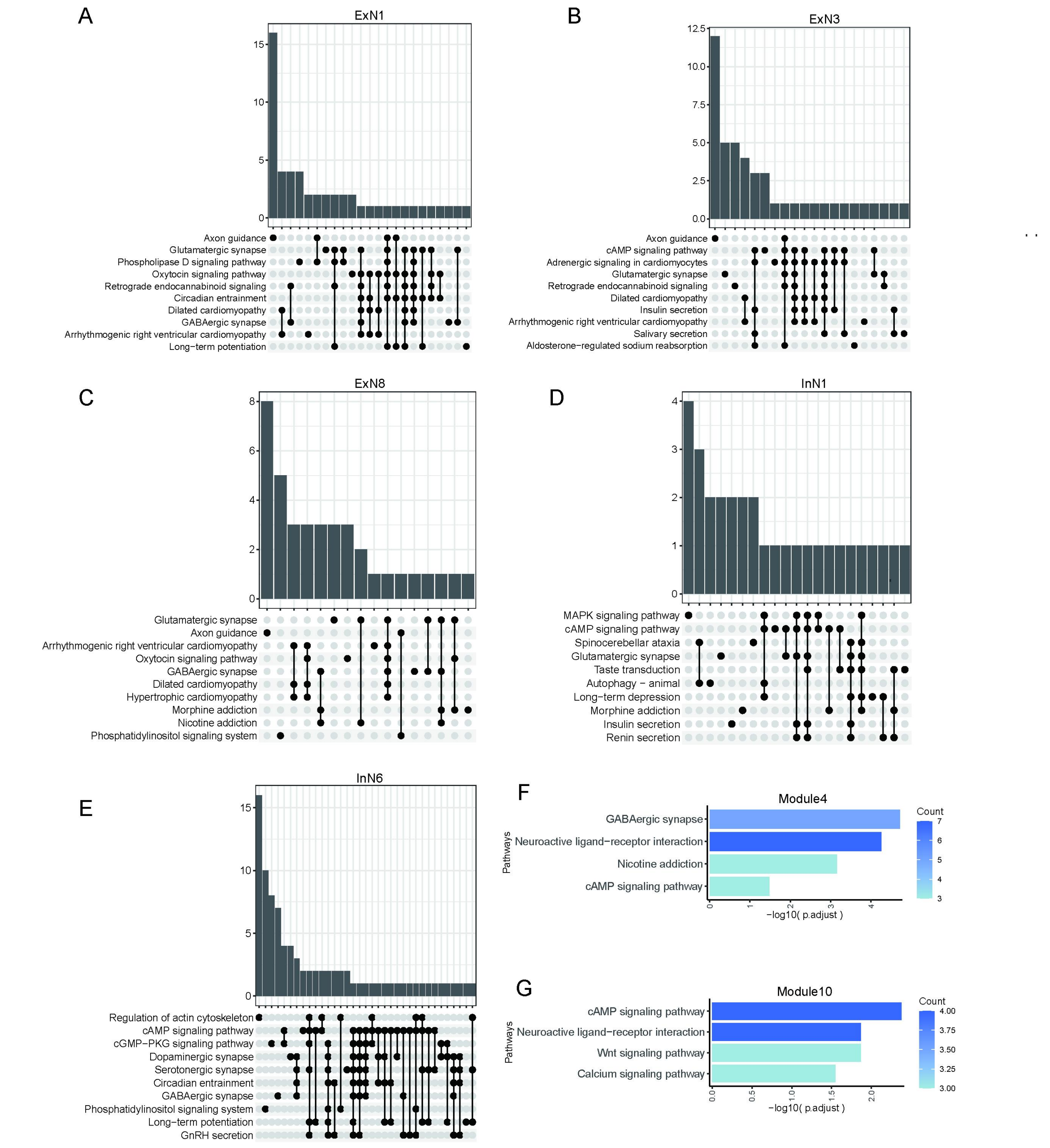

Figure S5. (related to Figure 3 and Figure 4)
(A-E) KEGG enrichment analysis of ExN1, ExN3, ExN8, InN1, and InN6.
The diagram shows genes shared or unique to each pathway. The black dots connecting the pathways represent genes shared by the pathways, and the barplot above represents the number of unique or shared genes.
(F-G) KEGG path enrichment analysis for Module 4 and Module 10. The horizontal coordinate is -log10(p.adjust) and the vertical coordinate is the relative pathway. The longer column stood for more significant enrichment data.

Figure S6

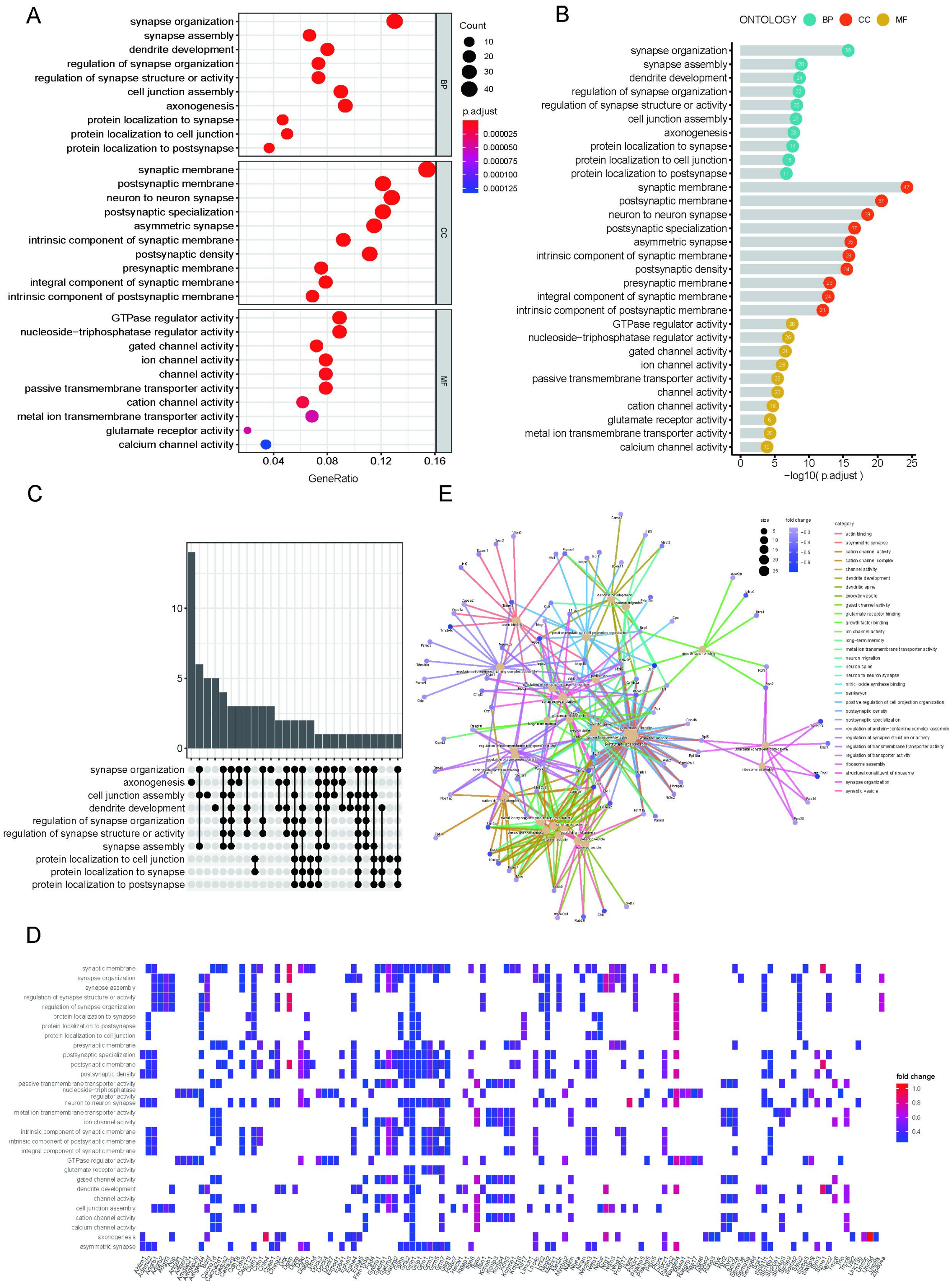

Figure S6. GO analysis of ExN1 potential functional modules (related to Figure 4)

(A) The bubble map shows the GO enrichment results of ExN1. The horizontal coordinate is GeneRatio and the vertical coordinate is GO Term. The color of the circle represents the value of p.adjust, and the size of the circle represents the number of differential genes.

(B) The enrichment results of each pathway showed that longer the lollipop column stood for more significant enrichment. The value in the circle represents the number of genes enriched to the pathway.

(C) Display of unique or shared genes of the pathway, the line represents the shared genes of the connected pathway, and the top column represents the number of unique or shared genes.

(D) The heatmap showed that there were strongly enriched genes in each pathway.

(E) The gene pathway association network map highlights the shared genes among the pathways and the highly expressed shared gene types.

Figure S7

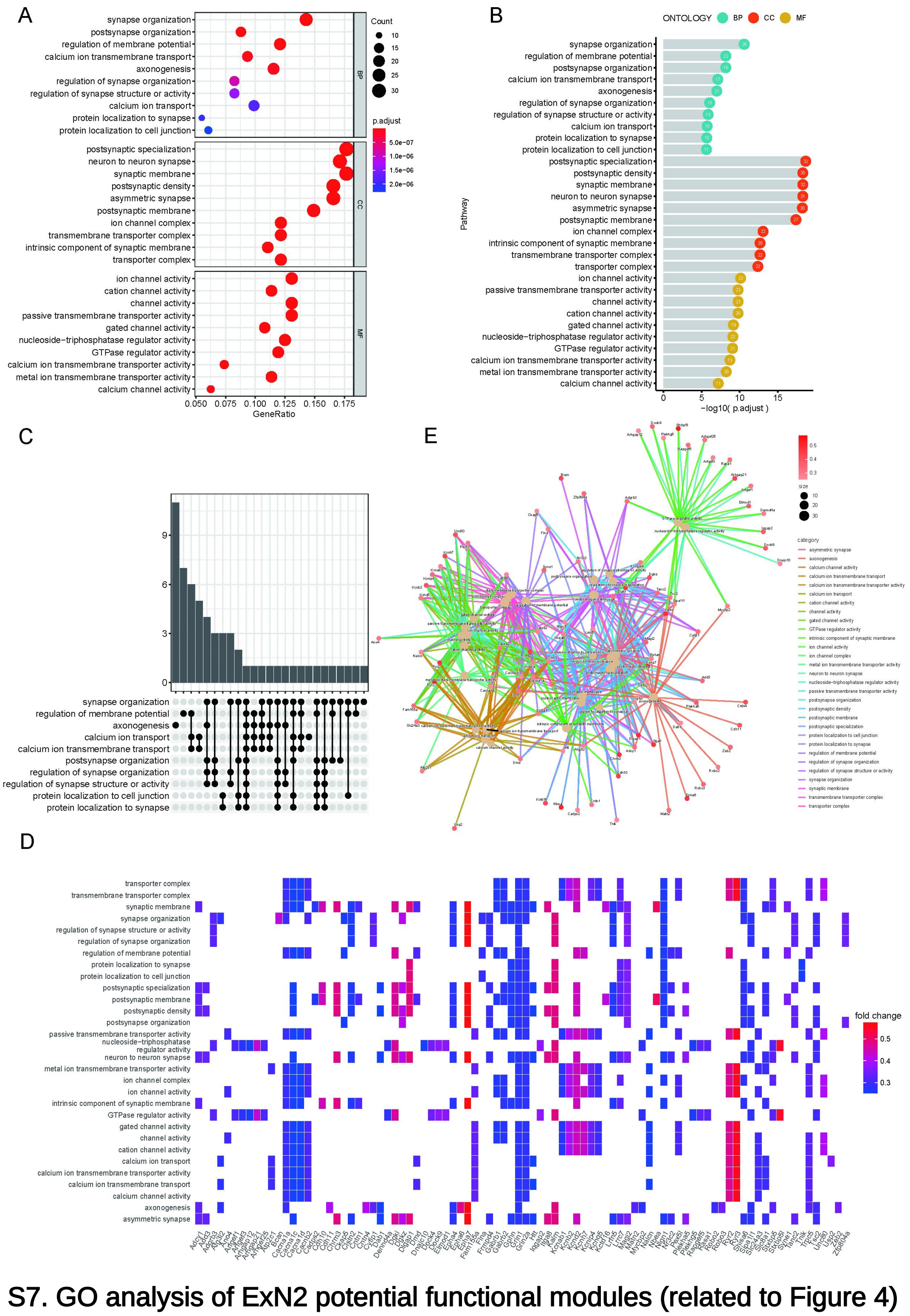

Figure S7. GO analysis of ExN2 potential functional modules (related to Figure 4)

(A) The bubble map shows the GO enrichment results of ExN1. The horizontal coordinate is GeneRatio and the vertical coordinate is GO Term. The color of the circle represents the value of p.adjust, and the size of the circle represents the number of differential genes.

(B) The enrichment results of each pathway showed that longer the lollipop column stood for more significant enrichment. The value in the circle represents the number of genes enriched to the pathway.

(C) Display of unique or shared genes of the pathway, the line represents the shared genes of the connected pathway, and the top column represents the number of unique or shared genes.

(D) The heatmap showed that there were strongly enriched genes in each pathway.

(E) The gene pathway association network map highlights the shared genes among the pathways and the highly expressed shared gene types.

Figure S8

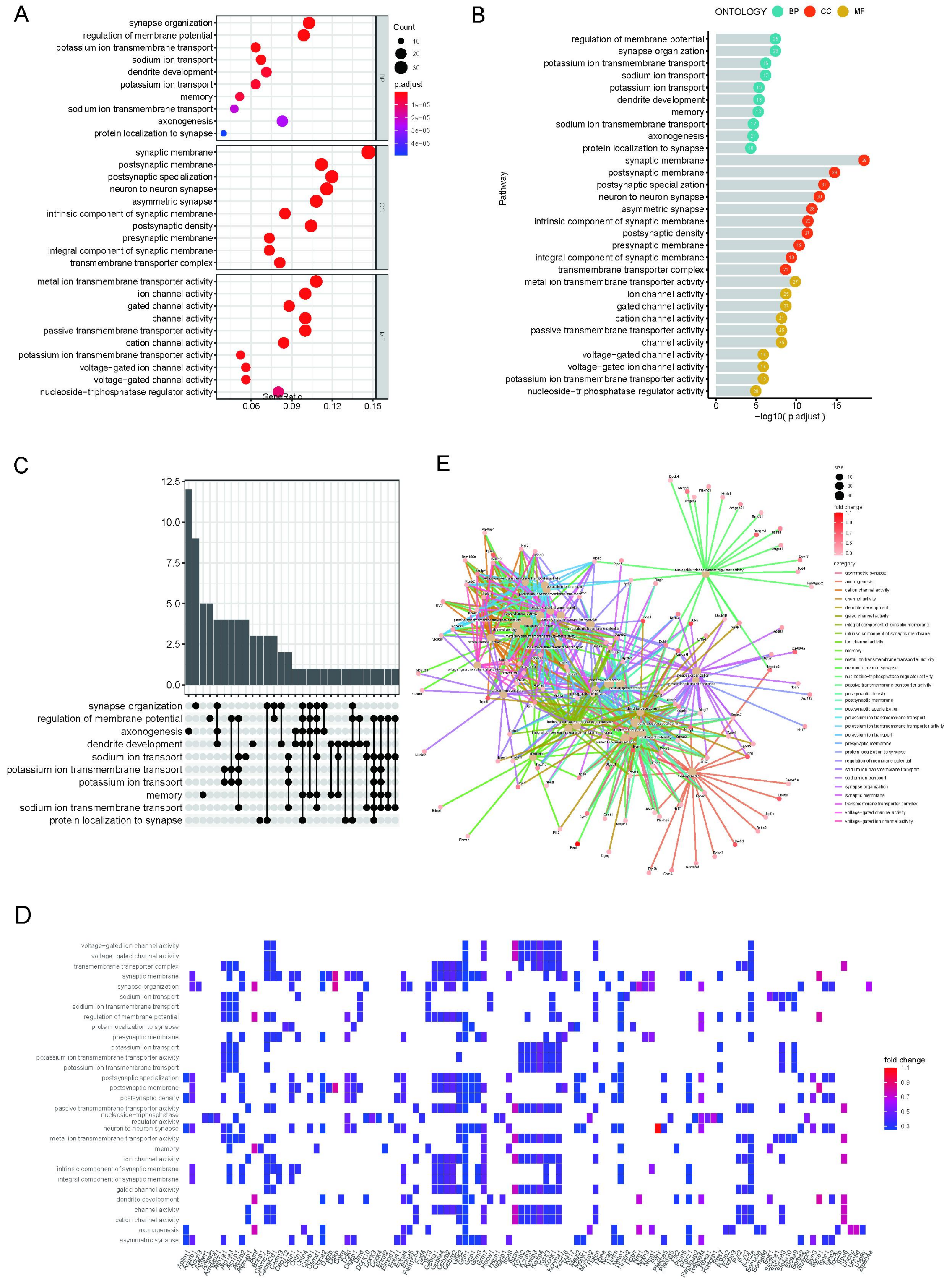

Figure S8. GO analysis of ExN3 potential functional modules (related to Figure 4)

(A) The bubble map shows the GO enrichment results of ExN1. The horizontal coordinate is GeneRatio and the vertical coordinate is GO Term. The color of the circle represents the value of p.adjust, and the size of the circle represents the number of differential genes.

(B) The enrichment results of each pathway showed that longer the lollipop column stood for more significant enrichment. The value in the circle represents the number of genes enriched to the pathway.

(C) Display of unique or shared genes of the pathway, the line represents the shared genes of the connected pathway, and the top column represents the number of unique or shared genes.

(D) The heatmap showed that there were strongly enriched genes in each pathway.

(E) The gene pathway association network map highlights the shared genes among the pathways and the highly expressed shared gene types.

Figure S9

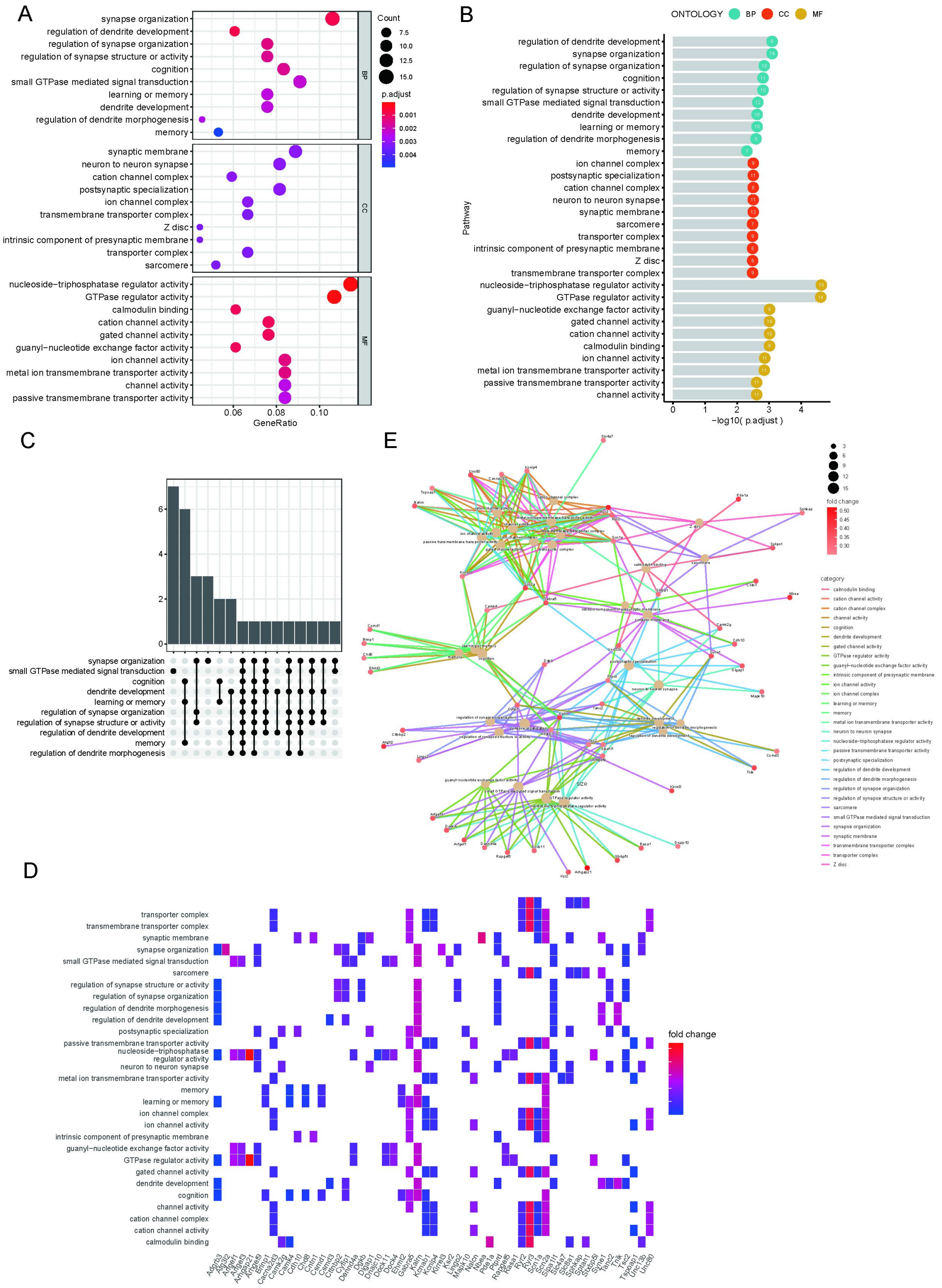

Figure S9. GO analysis of ExN4 potential functional modules (related to Figure 4)

(A) The bubble map shows the GO enrichment results of ExN1. The horizontal coordinate is GeneRatio and the vertical coordinate is GO Term. The color of the circle represents the value of p.adjust, and the size of the circle represents the number of differential genes.

(B) The enrichment results of each pathway showed that longer the lollipop column stood for more significant enrichment. The value in the circle represents the number of genes enriched to the pathway.

(C) Display of unique or shared genes of the pathway, the line represents the shared genes of the connected pathway, and the top column represents the number of unique or shared genes.

(D) The heatmap showed that there were strongly enriched genes in each pathway.

(E) The gene pathway association network map highlights the shared genes among the pathways and the highly expressed shared gene types.

Figure S10

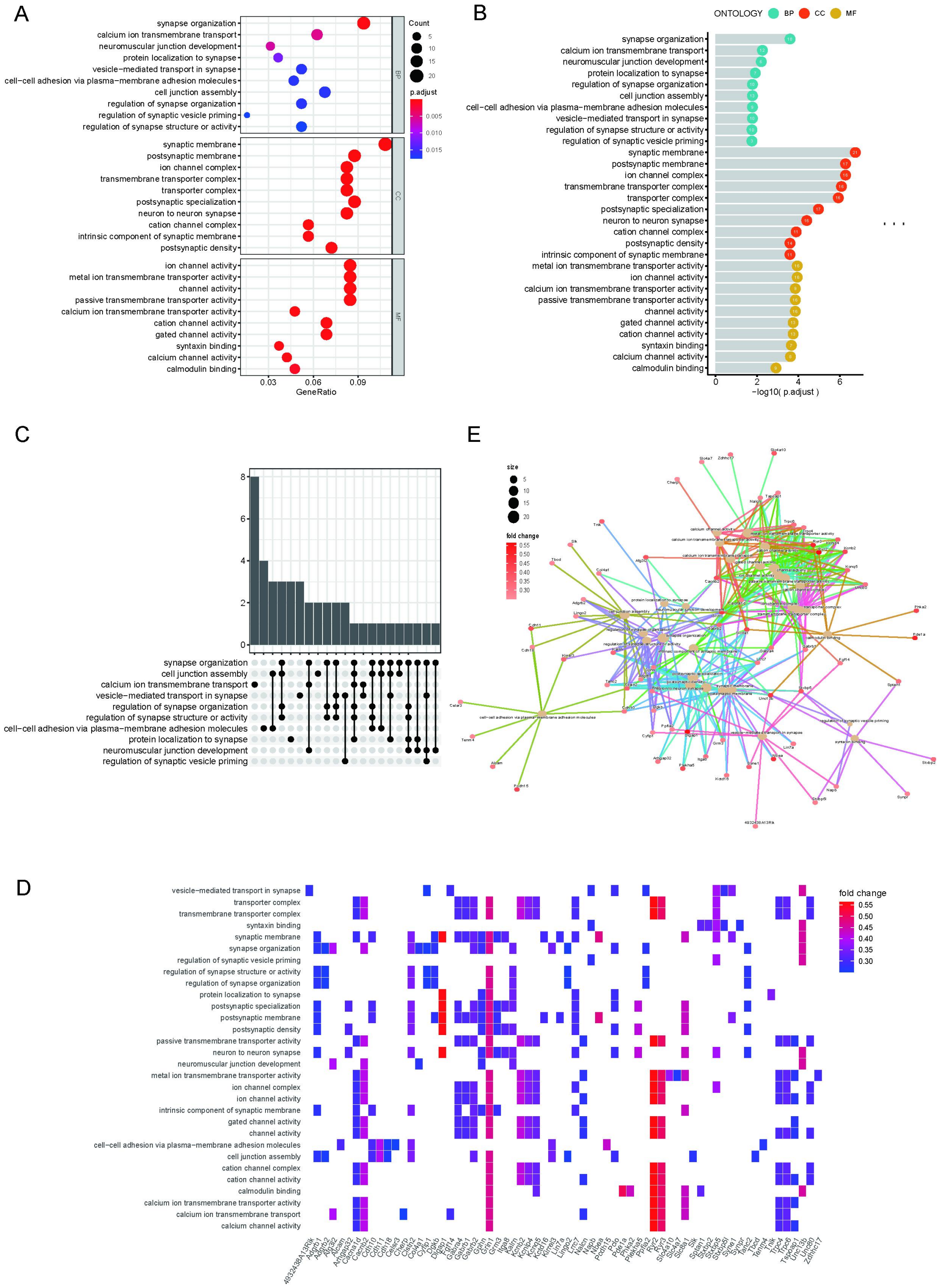

Figure S10. GO analysis of ExN5 potential functional modules (related to Figure 4)

(A) The bubble map shows the GO enrichment results of ExN1. The horizontal coordinate is GeneRatio and the vertical coordinate is GO Term. The color of the circle represents the value of p.adjust, and the size of the circle represents the number of differential genes.

(B) The enrichment results of each pathway showed that longer the lollipop column stood for more significant enrichment. The value in the circle represents the number of genes enriched to the pathway.

(C) Display of unique or shared genes of the pathway, the line represents the shared genes of the connected pathway, and the top column represents the number of unique or shared genes.

(D) The heatmap showed that there were strongly enriched genes in each pathway.

(E) The gene pathway association network map highlights the shared genes among the pathways and the highly expressed shared gene types.

Figure S11

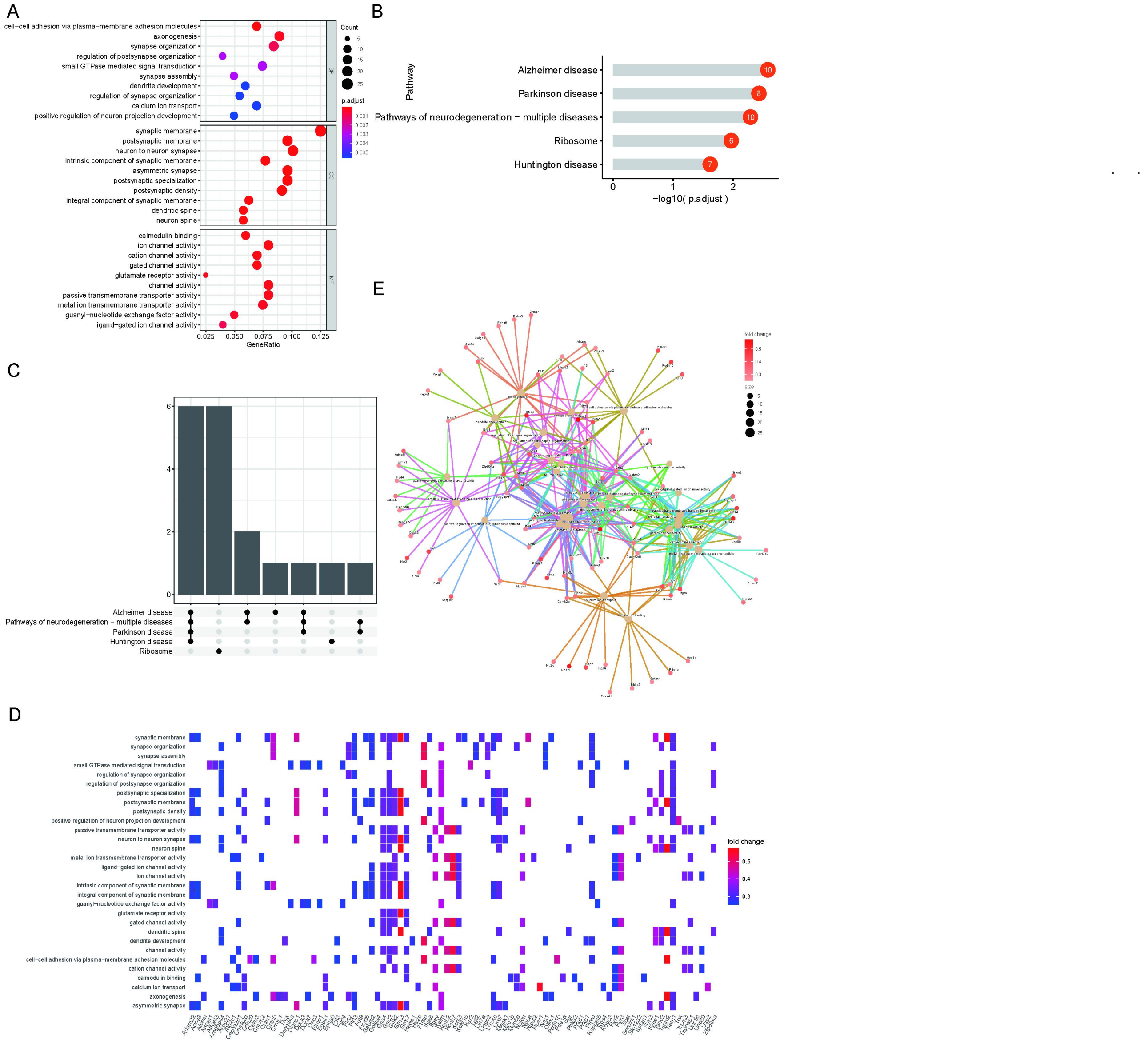

Figure S11. GO analysis of ExN6 potential functional modules (related to Figure 4)
(A) The bubble map shows the GO enrichment results of ExN1. The horizontal coordinate is GeneRatio and the vertical coordinate is GO Term. The color of the circle represents the value of p.adjust, and the size of the circle represents the number of differential genes.
(B) The enrichment results of each pathway showed that longer the lollipop column stood for more significant enrichment. The value in the circle represents the number of genes enriched to the pathway.
(C) Display of unique or shared genes of the pathway, the line represents the shared genes of the connected pathway, and the top column represents the number of unique or shared genes.
(D) The heatmap showed that there were strongly enriched genes in each pathway.
(E) The gene pathway association network map highlights the shared genes among the pathways and the highly expressed shared gene types.

Figure S12

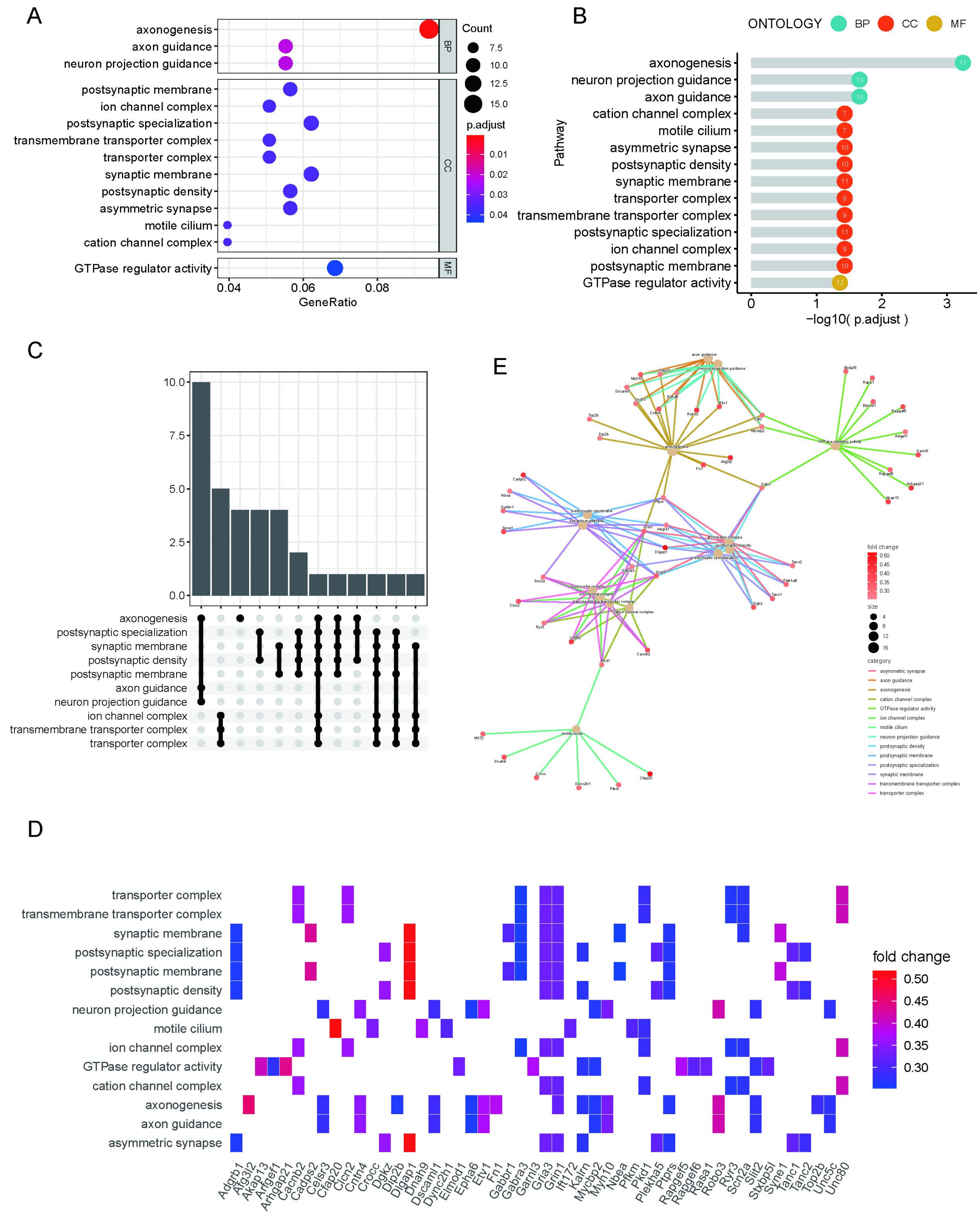

Figure S12. GO analysis of ExN7 potential functional modules (related to Figure 4)
(A) The bubble map shows the GO enrichment results of ExN1. The horizontal coordinate is GeneRatio and the vertical coordinate is GO Term. The color of the circle represents the value of p.adjust, and the size of the circle represents the number of differential genes.
(B) The enrichment results of each pathway showed that longer the lollipop column stood for more significant enrichment. The value in the circle represents the number of genes enriched to the pathway.
(C) Display of unique or shared genes of the pathway, the line represents the shared genes of the connected pathway, and the top column represents the number of unique or shared genes.
(D) The heatmap showed that there were strongly enriched genes in each pathway.
(E) The gene pathway association network map highlights the shared genes among the pathways and the highly expressed shared gene types.

Figure S13

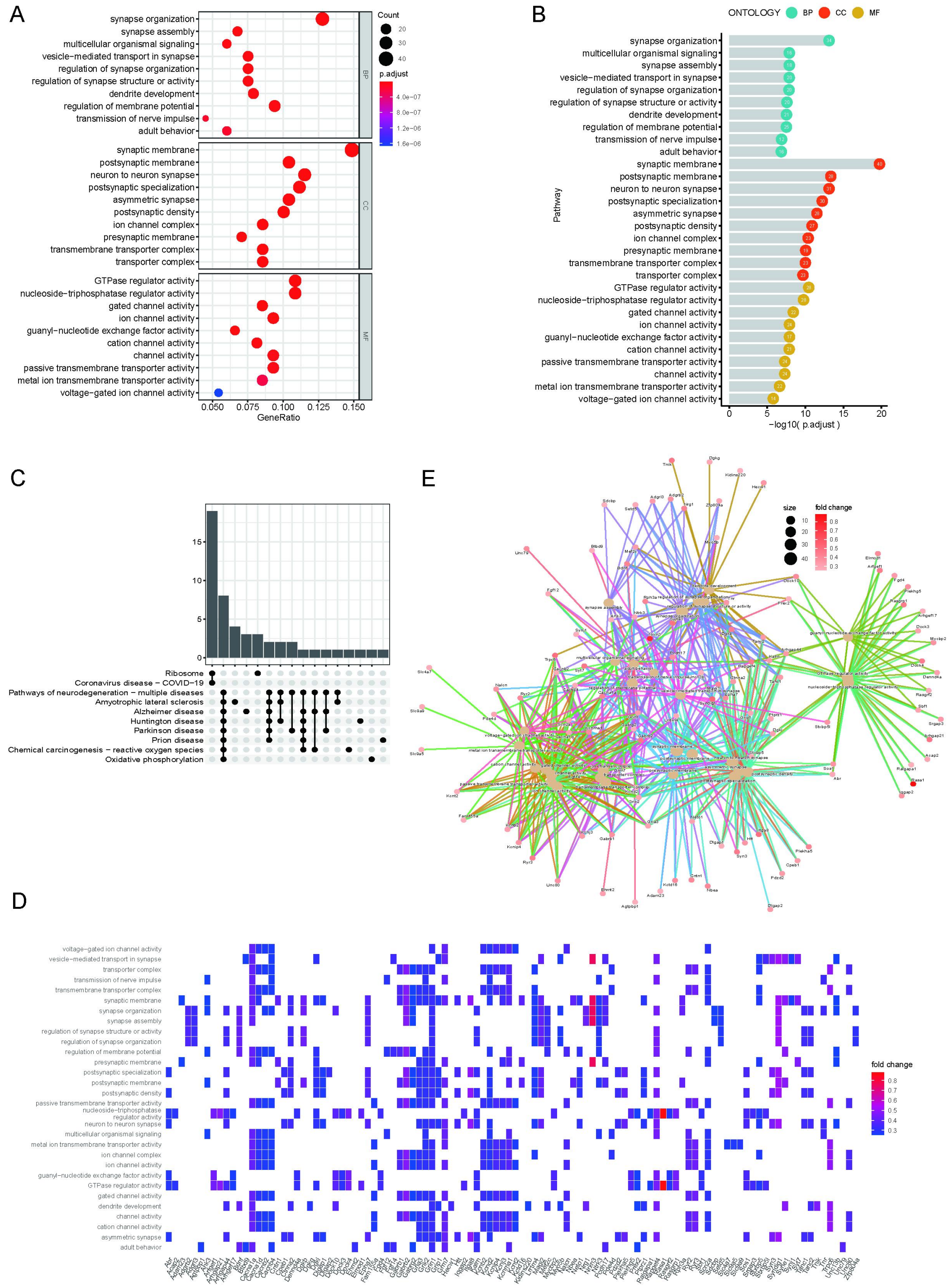

Figure S13. GO analysis of ExN8 potential functional modules (related to Figure 4)
(A) The bubble map shows the GO enrichment results of ExN1. The horizontal coordinate is GeneRatio and the vertical coordinate is GO Term. The color of the circle represents the value of p.adjust, and the size of the circle represents the number of differential genes.
(B) The enrichment results of each pathway showed that longer the lollipop column stood for more significant enrichment. The value in the circle represents the number of genes enriched to the pathway.
(C) Display of unique or shared genes of the pathway, the line represents the shared genes of the connected pathway, and the top column represents the number of unique or shared genes.
(D) The heatmap showed that there were strongly enriched genes in each pathway.
(E) The gene pathway association network map highlights the shared genes among the pathways and the highly expressed shared gene types.

Figure S14

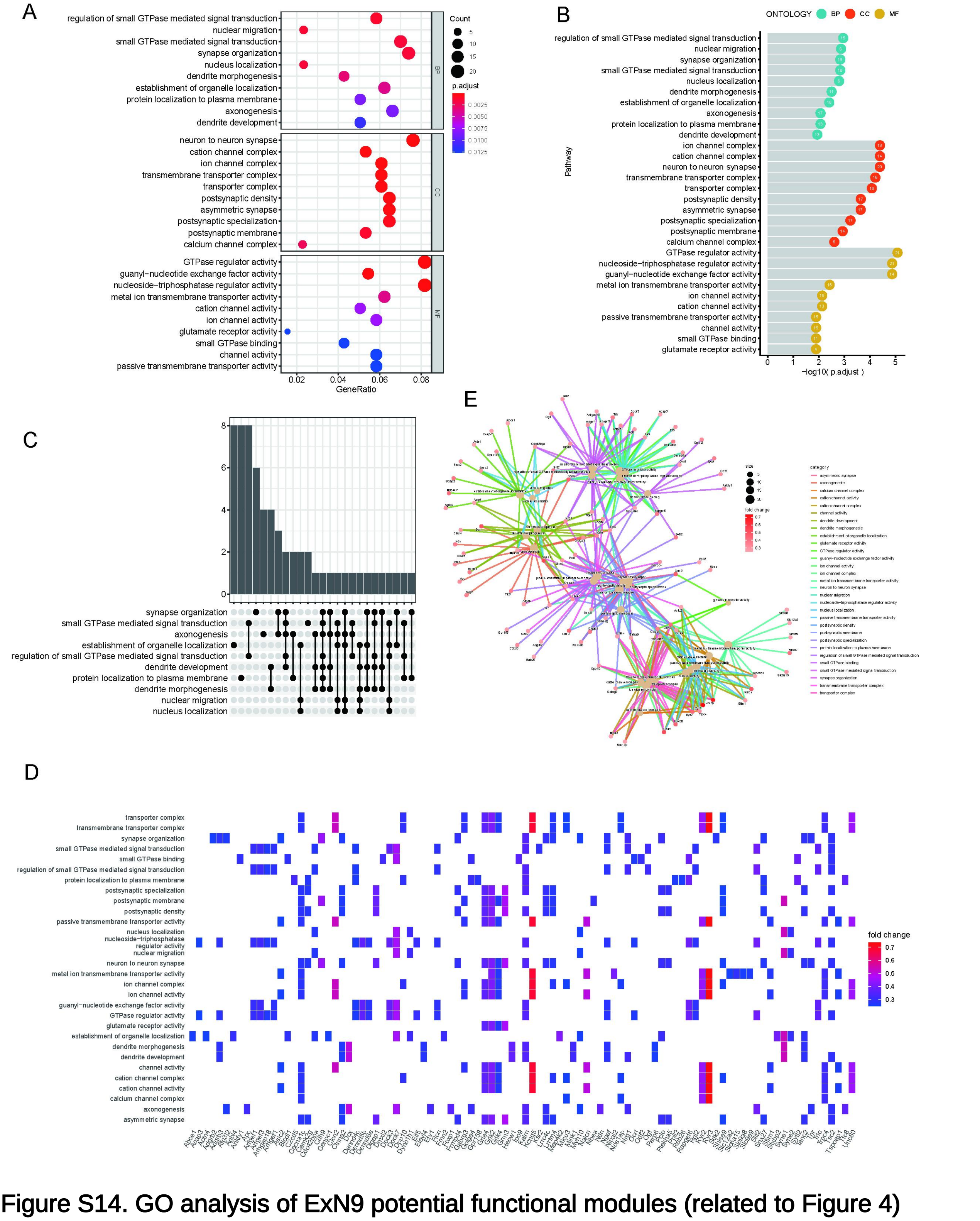

Figure S14. GO analysis of ExN9 potential functional modules (related to Figure 4)

(A) The bubble map shows the GO enrichment results of ExN1. The horizontal coordinate is GeneRatio and the vertical coordinate is GO Term. The color of the circle represents the value of p.adjust, and the size of the circle represents the number of differential genes.

(B) The enrichment results of each pathway showed that longer the lollipop column stood for more significant enrichment. The value in the circle represents the number of genes enriched to the pathway.

(C) Display of unique or shared genes of the pathway, the line represents the shared genes of the connected pathway, and the top column represents the number of unique or shared genes.

(D) The heatmap showed that there were strongly enriched genes in each pathway.

(E) The gene pathway association network map highlights the shared genes among the pathways and the highly expressed shared gene types.

Figure S15

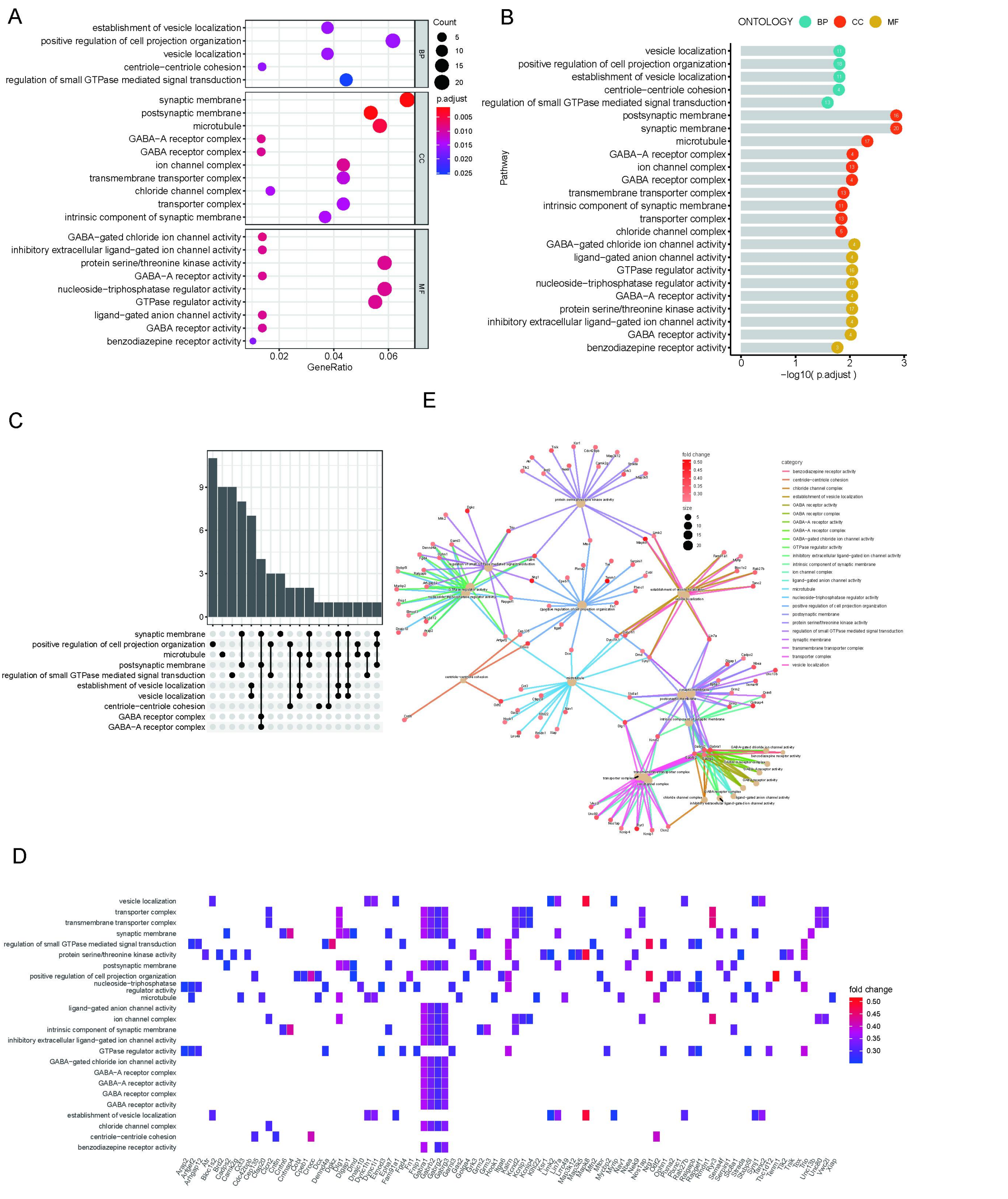

Figure S15. GO analysis of ExN10 potential functional modules (related to Figure 4)
(A) The bubble map shows the GO enrichment results of ExN1. The horizontal coordinate is GeneRatio and the vertical coordinate is GO Term. The color of the circle represents the value of p.adjust, and the size of the circle represents the number of differential genes.
(B) The enrichment results of each pathway showed that longer the lollipop column stood for more significant enrichment. The value in the circle represents the number of genes enriched to the pathway.
(C) Display of unique or shared genes of the pathway, the line represents the shared genes of the connected pathway, and the top column represents the number of unique or shared genes.
(D) The heatmap showed that there were strongly enriched genes in each pathway.
(E) The gene pathway association network map highlights the shared genes among the pathways and the highly expressed shared gene types.

Figure S16

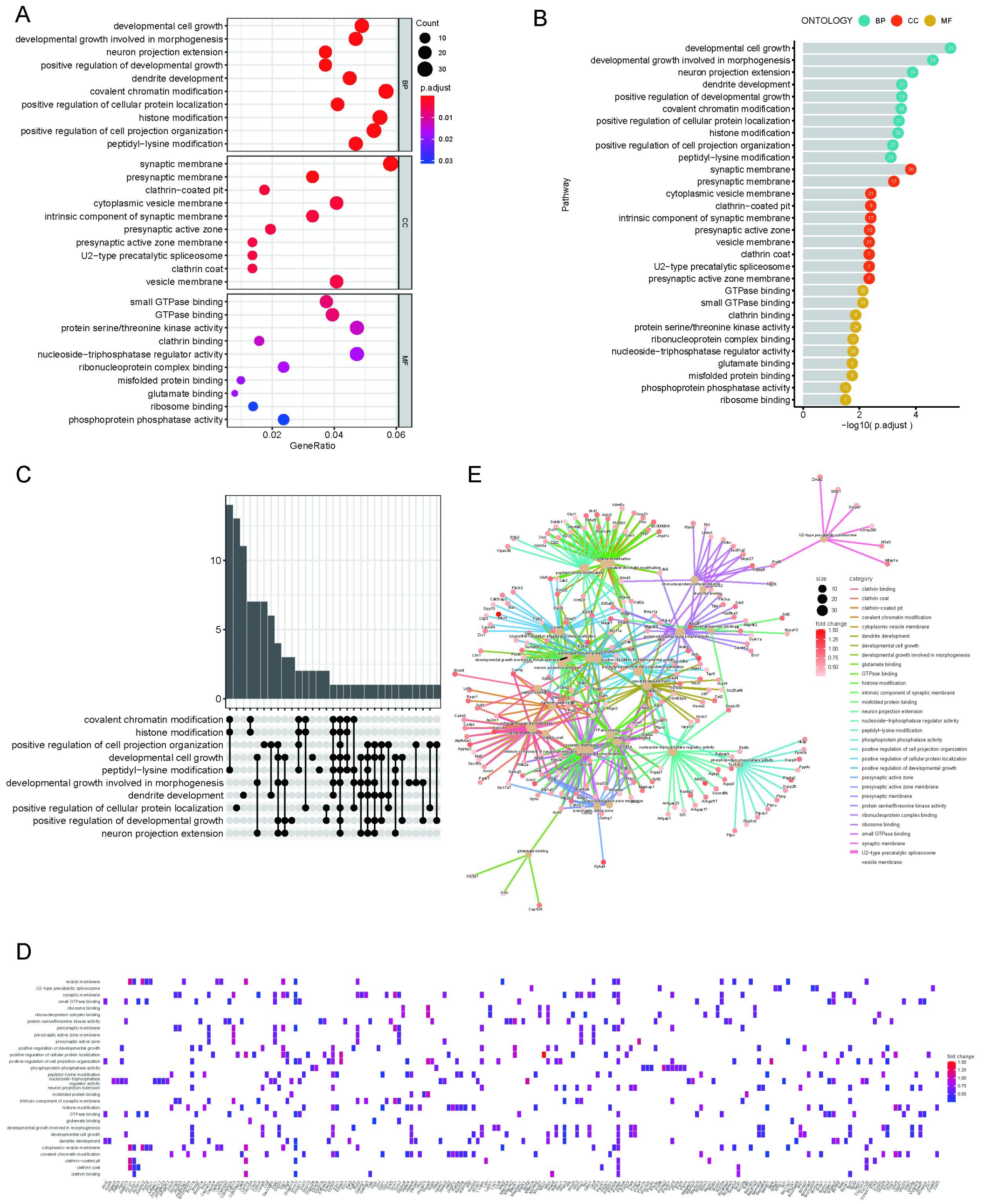

Figure S16. GO analysis of ExN11 potential functional modules (related to Figure 4)

(A) The bubble map shows the GO enrichment results of ExN1. The horizontal coordinate is GeneRatio and the vertical coordinate is GO Term. The color of the circle represents the value of p.adjust, and the size of the circle represents the number of differential genes.

(B) The enrichment results of each pathway showed that longer the lollipop column stood for more significant enrichment. The value in the circle represents the number of genes enriched to the pathway.

(C) Display of unique or shared genes of the pathway, the line represents the shared genes of the connected pathway, and the top column represents the number of unique or shared genes.

(D) The heatmap showed that there were strongly enriched genes in each pathway.

(E) The gene pathway association network map highlights the shared genes among the pathways and the highly expressed shared gene types.

Figure S17

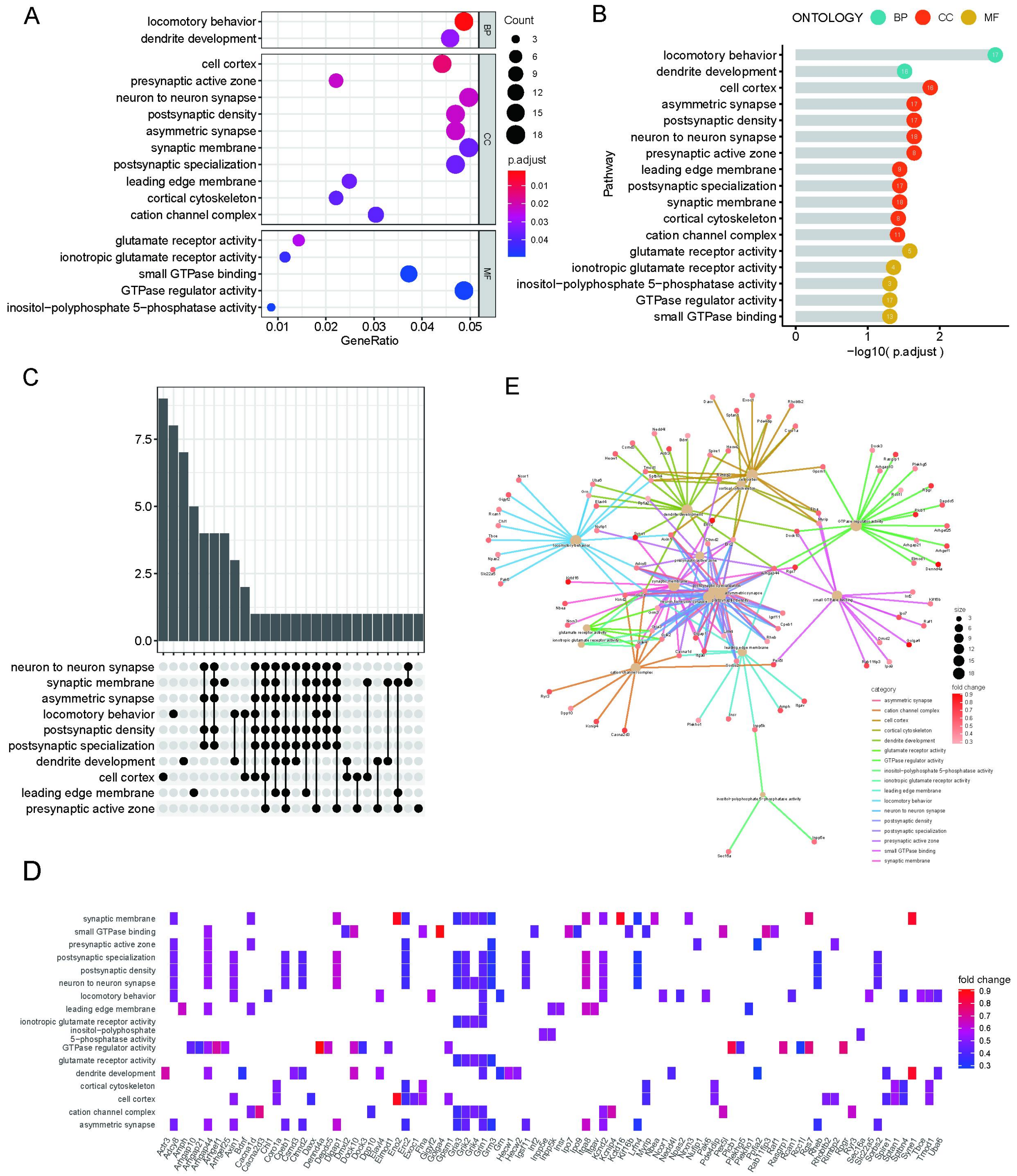

Figure S17. GO analysis of ExN12 potential functional modules (related to Figure 4)
(A) The bubble map shows the GO enrichment results of ExN1. The horizontal coordinate is GeneRatio and the vertical coordinate is GO Term. The color of the circle represents the value of p.adjust, and the size of the circle represents the number of differential genes.
(B) The enrichment results of each pathway showed that longer the lollipop column stood for more significant enrichment. The value in the circle represents the number of genes enriched to the pathway.
(C) Display of unique or shared genes of the pathway, the line represents the shared genes of the connected pathway, and the top column represents the number of unique or shared genes.
(D) The heatmap showed that there were strongly enriched genes in each pathway.
(E) The gene pathway association network map highlights the shared genes among the pathways and the highly expressed shared gene types.

Figure S18

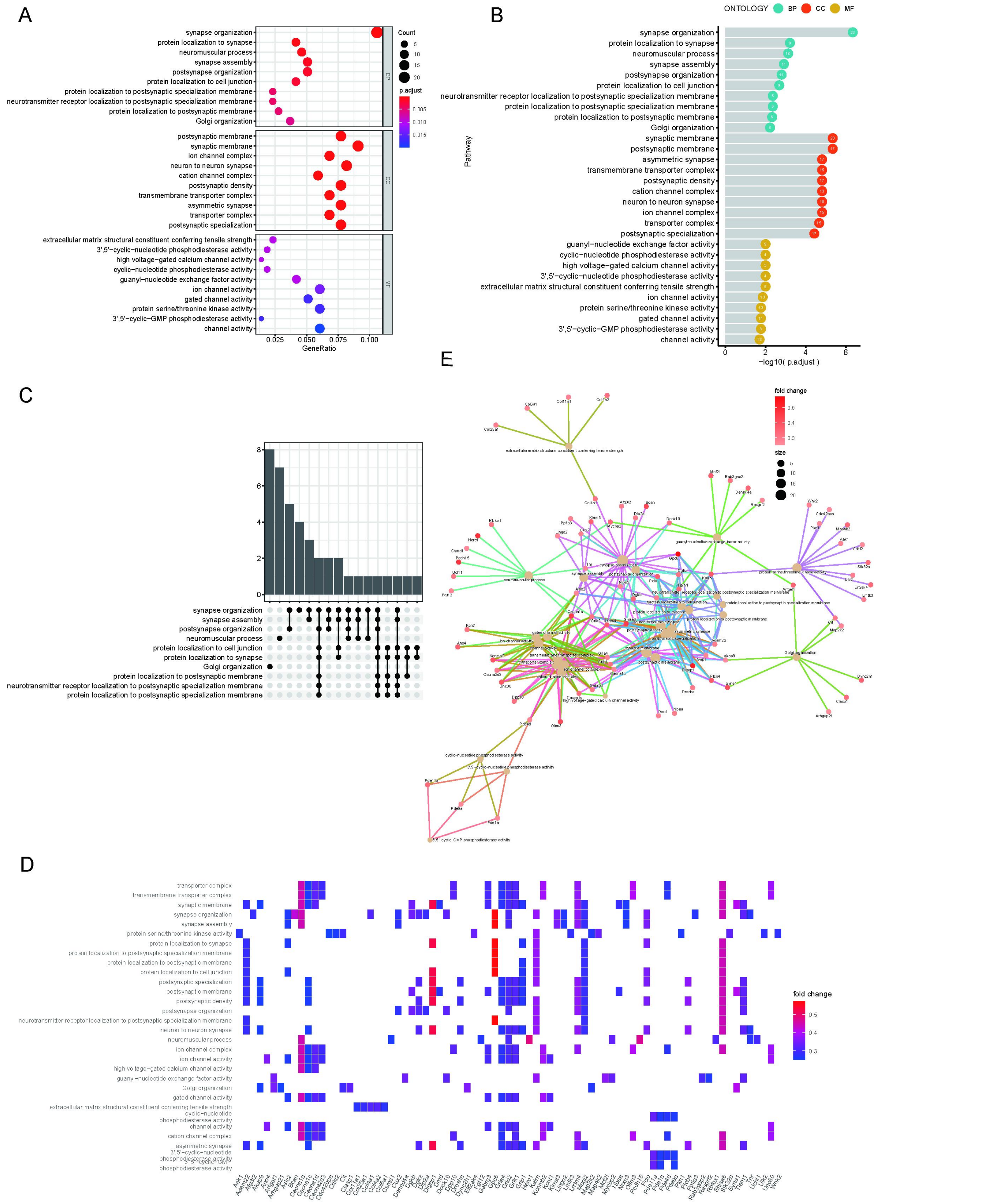

Figure S18. GO analysis of InN1 potential functional modules (related to Figure 4)

(A) The bubble map shows the GO enrichment results of ExN1. The horizontal coordinate is GeneRatio and the vertical coordinate is GO Term. The color of the circle represents the value of p.adjust, and the size of the circle represents the number of differential genes.

(B) The enrichment results of each pathway showed that longer the lollipop column stood for more significant enrichment. The value in the circle represents the number of genes enriched to the pathway.

(C) Display of unique or shared genes of the pathway, the line represents the shared genes of the connected pathway, and the top column represents the number of unique or shared genes.

(D) The heatmap showed that there were strongly enriched genes in each pathway.

(E) The gene pathway association network map highlights the shared genes among the pathways and the highly expressed shared gene types.

Figure S19

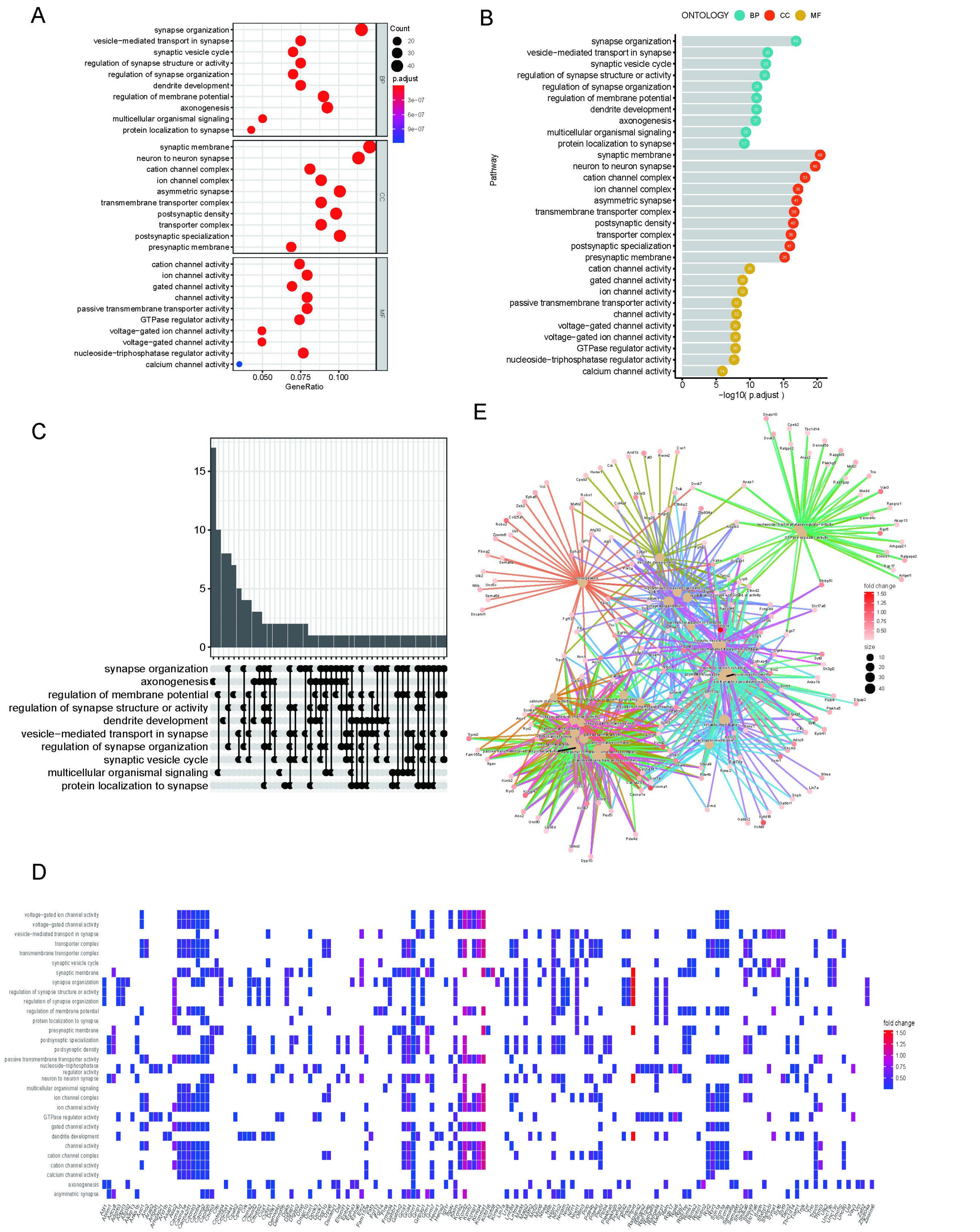

Figure S19. GO analysis of InN2 potential functional modules (related to Figure 4)

(A) The bubble map shows the GO enrichment results of ExN1. The horizontal coordinate is GeneRatio and the vertical coordinate is GO Term. The color of the circle represents the value of p.adjust, and the size of the circle represents the number of differential genes.

(B) The enrichment results of each pathway showed that longer the lollipop column stood for more significant enrichment. The value in the circle represents the number of genes enriched to the pathway.

(C) Display of unique or shared genes of the pathway, the line represents the shared genes of the connected pathway, and the top column represents the number of unique or shared genes.

(D) The heatmap showed that there were strongly enriched genes in each pathway.

(E) The gene pathway association network map highlights the shared genes among the pathways and the highly expressed shared gene types.

Figure S20

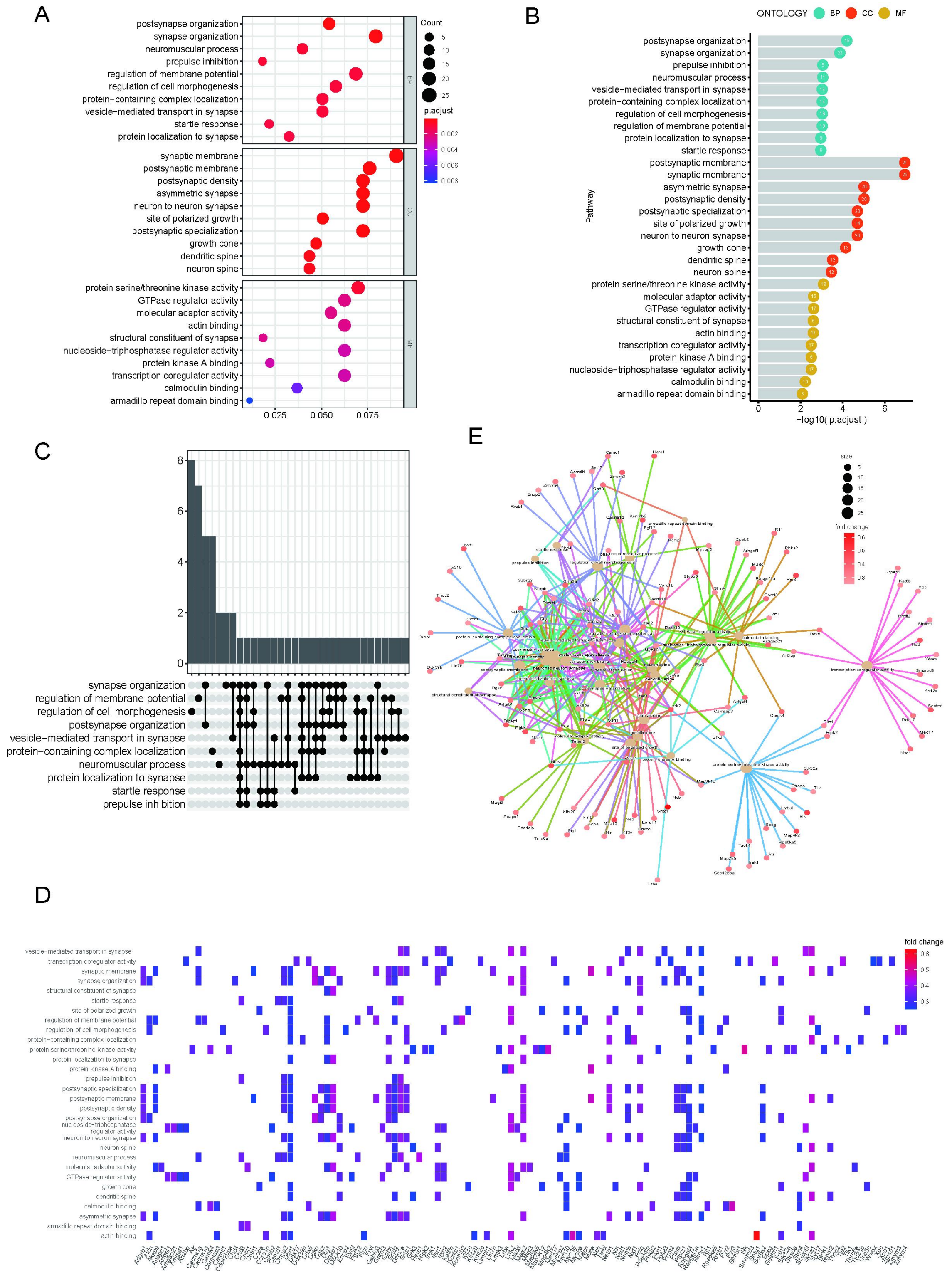

Figure S20. GO analysis of InN3 potential functional modules (related to Figure 4 and Figure 5)

(A) The bubble map shows the GO enrichment results of ExN1. The horizontal coordinate is GeneRatio and the vertical coordinate is GO Term. The color of the circle represents the value of p.adjust, and the size of the circle represents the number of differential genes.

(B) The enrichment results of each pathway showed that longer the lollipop column stood for more significant enrichment. The value in the circle represents the number of genes enriched to the pathway.

(C) Display of unique or shared genes of the pathway, the line represents the shared genes of the connected pathway, and the top column represents the number of unique or shared genes.

(D) The heatmap showed that there were strongly enriched genes in each pathway.

(E) The gene pathway association network map highlights the shared genes among the pathways and the highly expressed shared gene types.

Figure S21

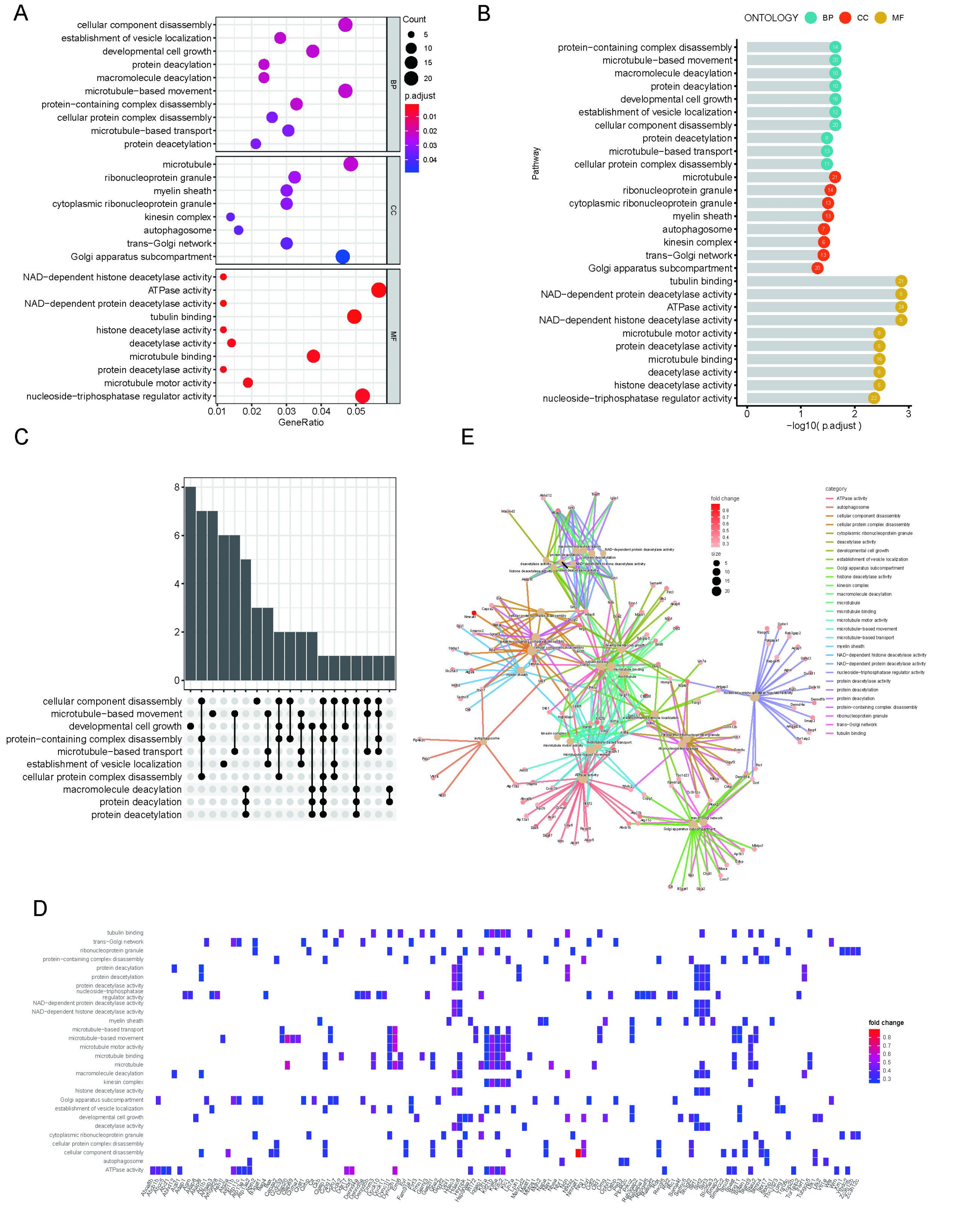

Figure S21. GO analysis of InN4 potential functional modules (related to Figure 4)

(A) The bubble map shows the GO enrichment results of ExN1. The horizontal coordinate is GeneRatio and the vertical coordinate is GO Term. The color of the circle represents the value of p.adjust, and the size of the circle represents the number of differential genes.

(B) The enrichment results of each pathway showed that longer the lollipop column stood for more significant enrichment. The value in the circle represents the number of genes enriched to the pathway.

(C) Display of unique or shared genes of the pathway, the line represents the shared genes of the connected pathway, and the top column represents the number of unique or shared genes.

(D) The heatmap showed that there were strongly enriched genes in each pathway.

(E) The gene pathway association network map highlights the shared genes among the pathways and the highly expressed shared gene types.

Figure S22

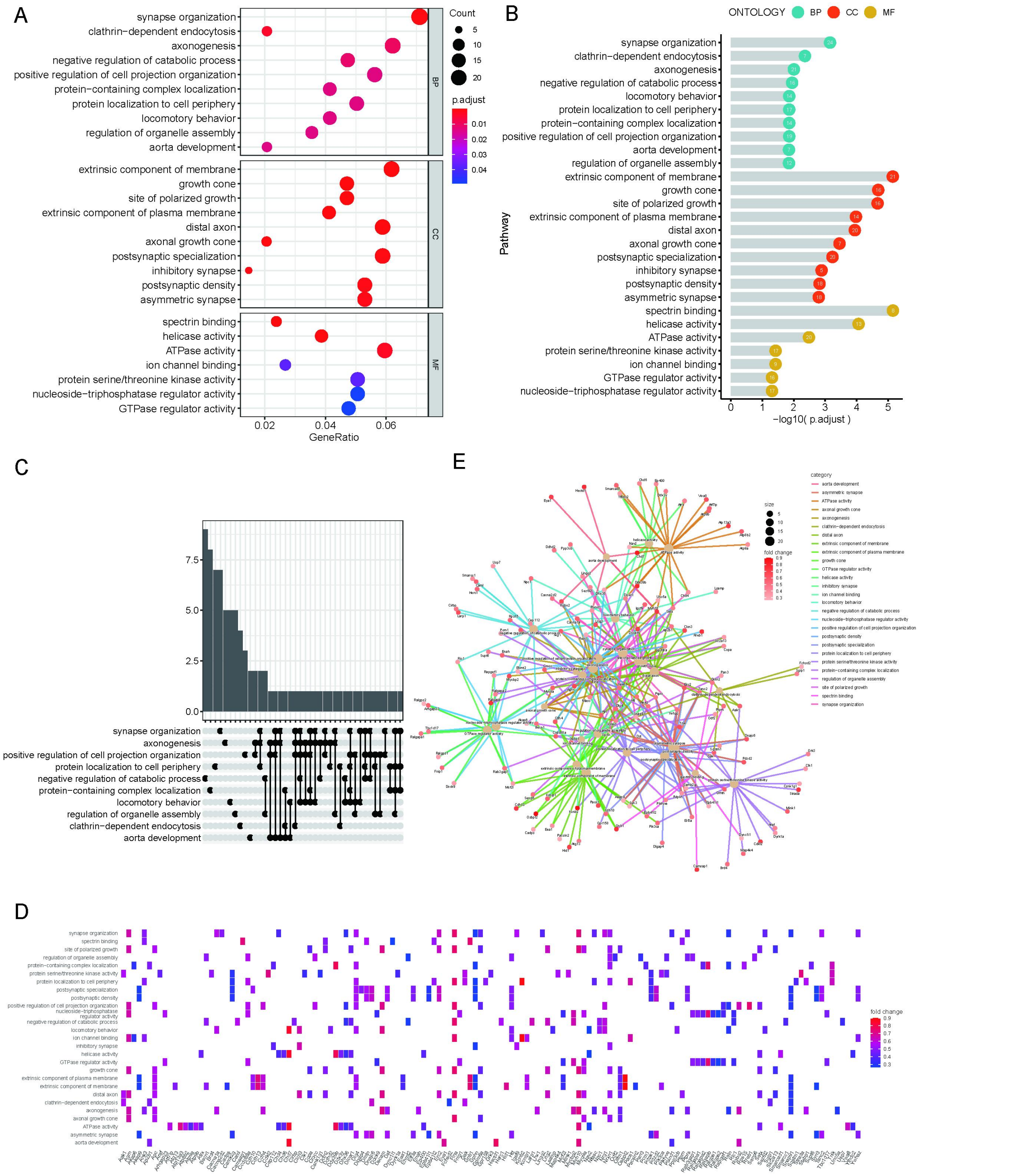

Figure S22. GO analysis of InN5 potential functional modules (related to Figure 4)

(A) The bubble map shows the GO enrichment results of ExN1. The horizontal coordinate is GeneRatio and the vertical coordinate is GO Term. The color of the circle represents the value of p.adjust, and the size of the circle represents the number of differential genes.

(B) The enrichment results of each pathway showed that longer the lollipop column stood for more significant enrichment. The value in the circle represents the number of genes enriched to the pathway.

(C) Display of unique or shared genes of the pathway, the line represents the shared genes of the connected pathway, and the top column represents the number of unique or shared genes.

(D) The heatmap showed that there were strongly enriched genes in each pathway.

(E) The gene pathway association network map highlights the shared genes among the pathways and the highly expressed shared gene types.

Figure S23

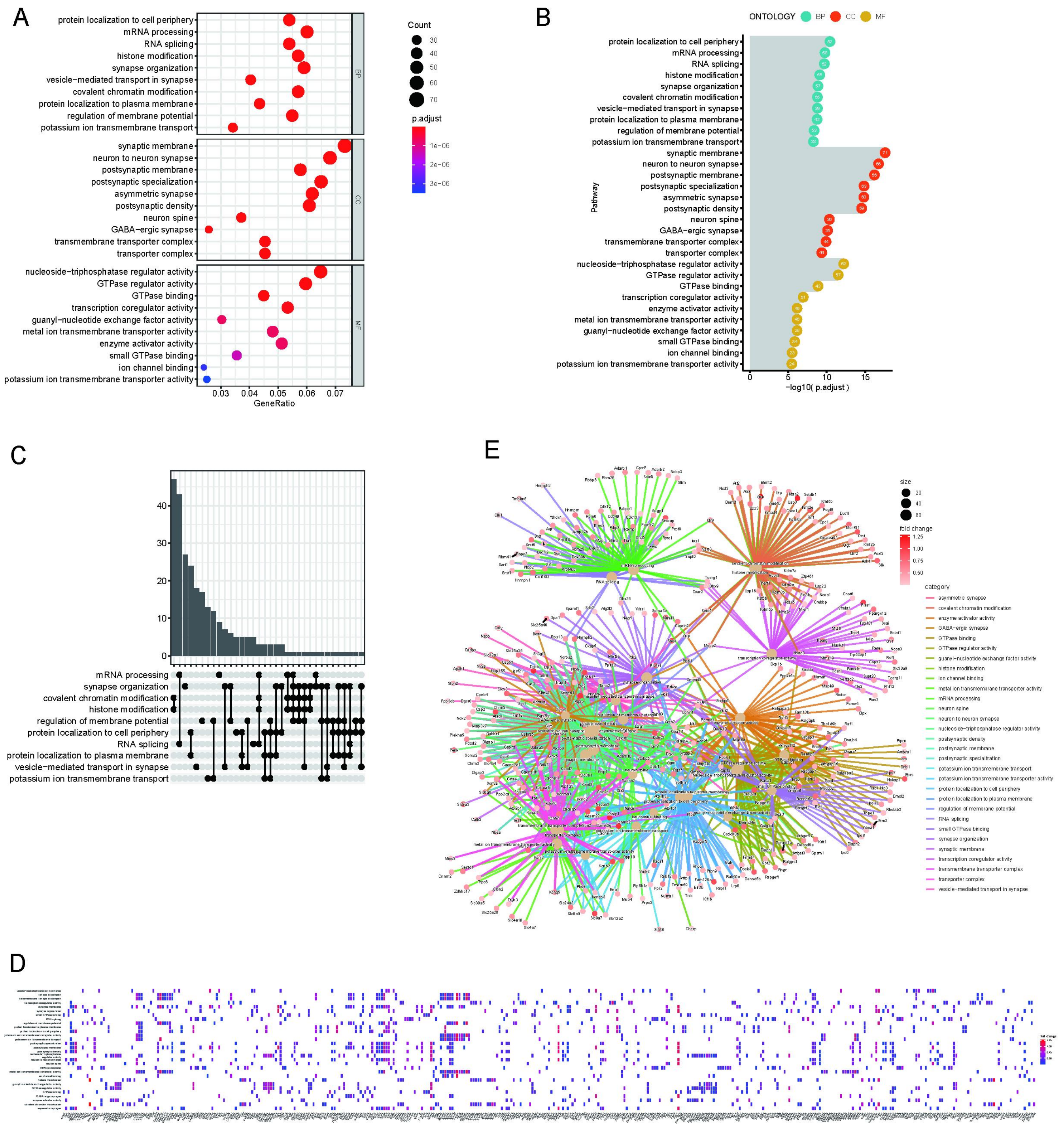

Figure S23. GO analysis of InN6 potential functional modules (related to Figure 4)

(A) The bubble map shows the GO enrichment results of ExN1. The horizontal coordinate is GeneRatio and the vertical coordinate is GO Term. The color of the circle represents the value of p.adjust, and the size of the circle represents the number of differential genes.

(B) The enrichment results of each pathway showed that longer the lollipop column stood for more significant enrichment. The value in the circle represents the number of genes enriched to the pathway.

(C) Display of unique or shared genes of the pathway, the line represents the shared genes of the connected pathway, and the top column represents the number of unique or shared genes.

(D) The heatmap showed that there were strongly enriched genes in each pathway.

(E) The gene pathway association network map highlights the shared genes among the pathways and the highly expressed shared gene types.

Figure S24

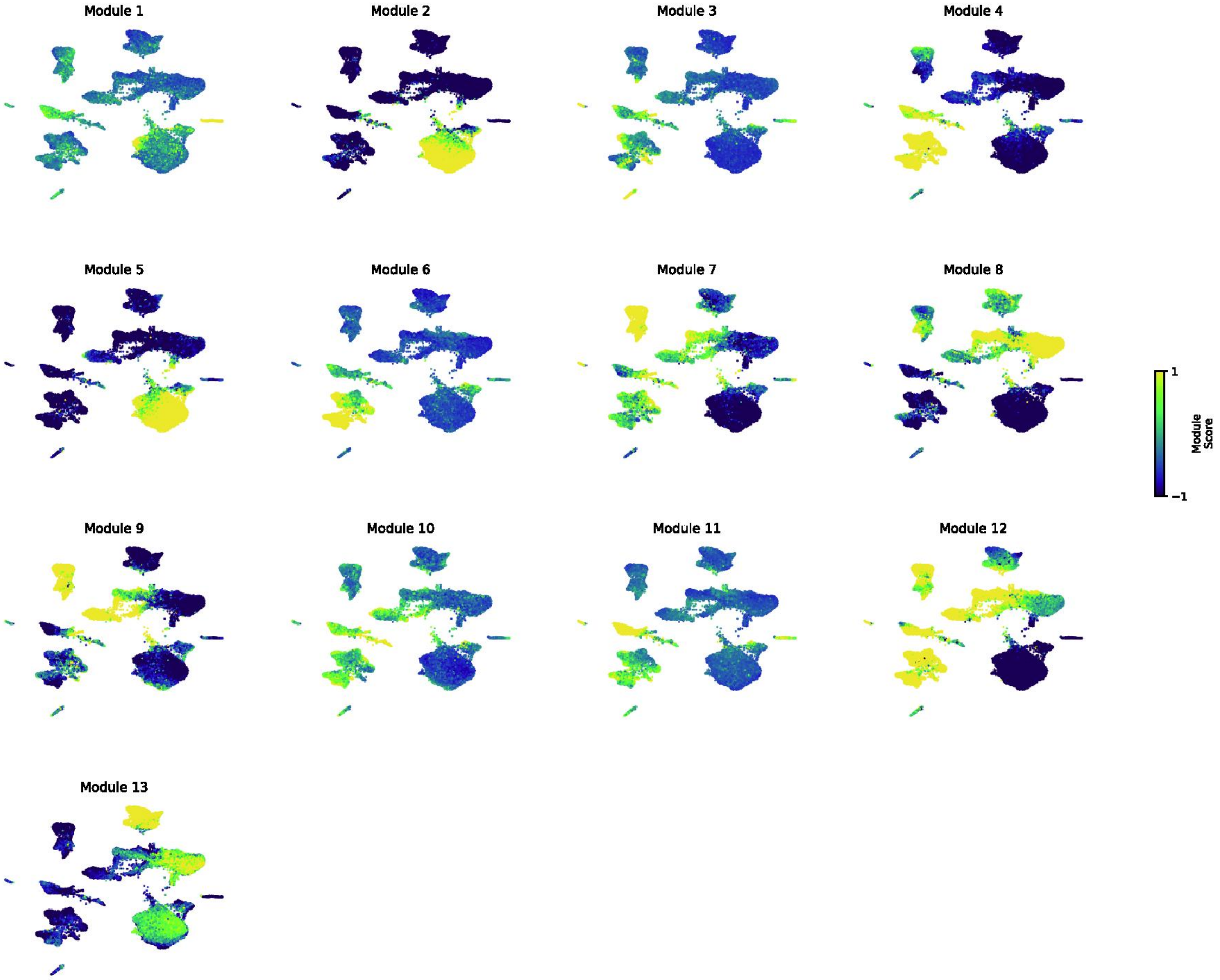

Figure S24. Similarity analysis of differential genes between Module and neuron subgroups (related to Figure 5)

(A) Gene set score reduction diagram. Each Module is a gene set, and the lighter color indicates the more significant type expressions of the gene cluster of the cell population, suggesting the function of the indicated Module was more similar.

Figure S25

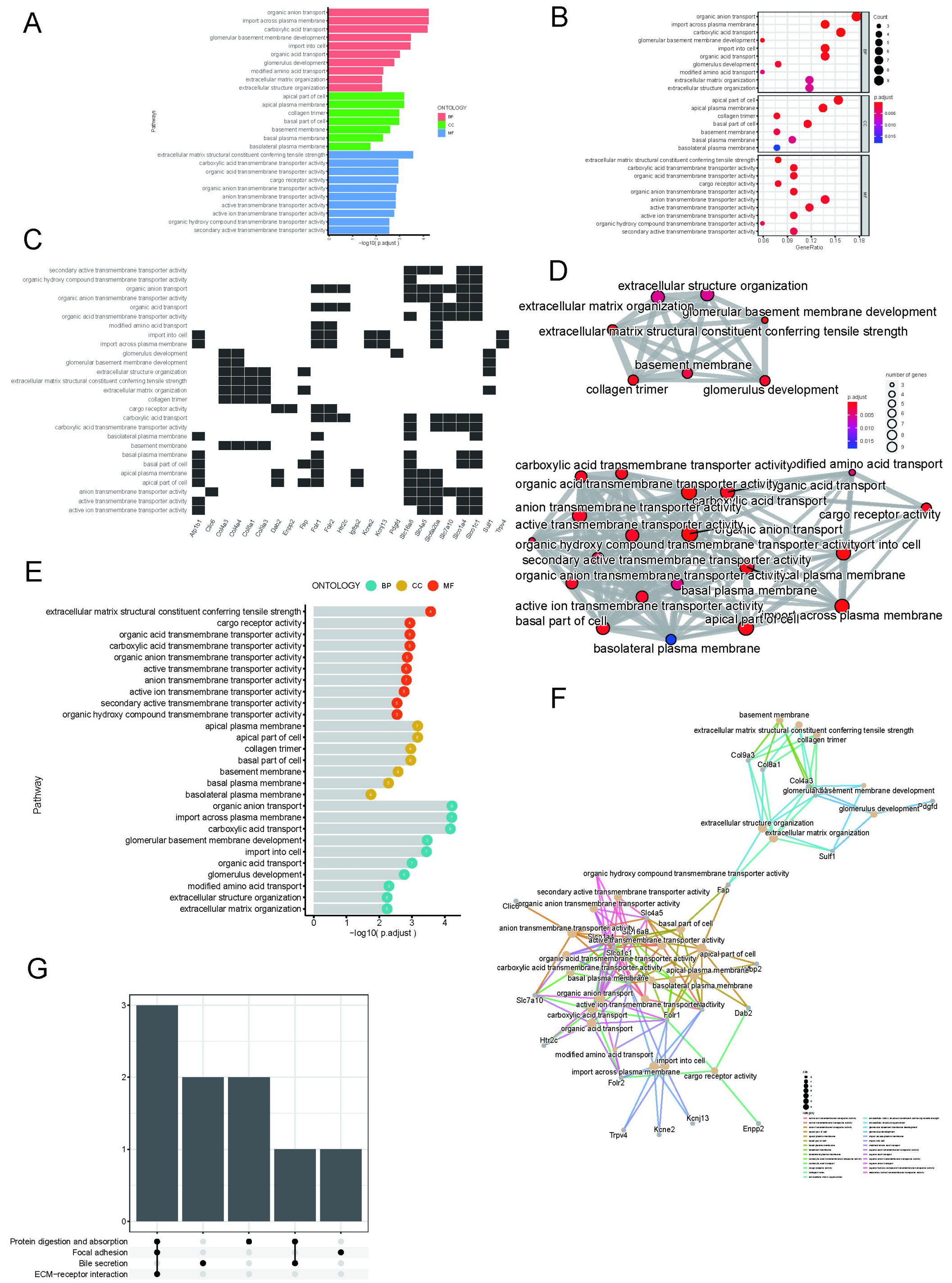

Figure S25. GO analysis of Module 1 functional categories (related to Figure 5)

(A) Enrichment of BP, CC, and MF pathways in Module 1. The horizontal coordinate was -log10(p.adjust) and the vertical coordinate was GO Term. The longer column stands for the more significant enrichment.

(B) Bubble diagram of BP, CC, and MF enrichment in Module 1, indicating the relationship between GeneRatio and GO Term.

(C) Gene enrichment of each pathway, where squares indicated the gene belonging to the pathway.

(D) Based on the network diagram of pathway gene sharing and similarity, the pathways with high coincidence degrees were clustered together, which facilitated the identification of functional modules.

(E) The number value in the circle showed the number of genes enriched in each pathway of Module 1. The longer the lollipop column stood for the more significant the enrichment.

(F) The map of the association network between genes and pathways showed the molecules shared between each pathway.

(G) The unique or shared genes of each pathway. The black dots on the lines between pathways represent genes shared by the pathways, and the top bar represents the number of genes unique or shared.

Figure S26

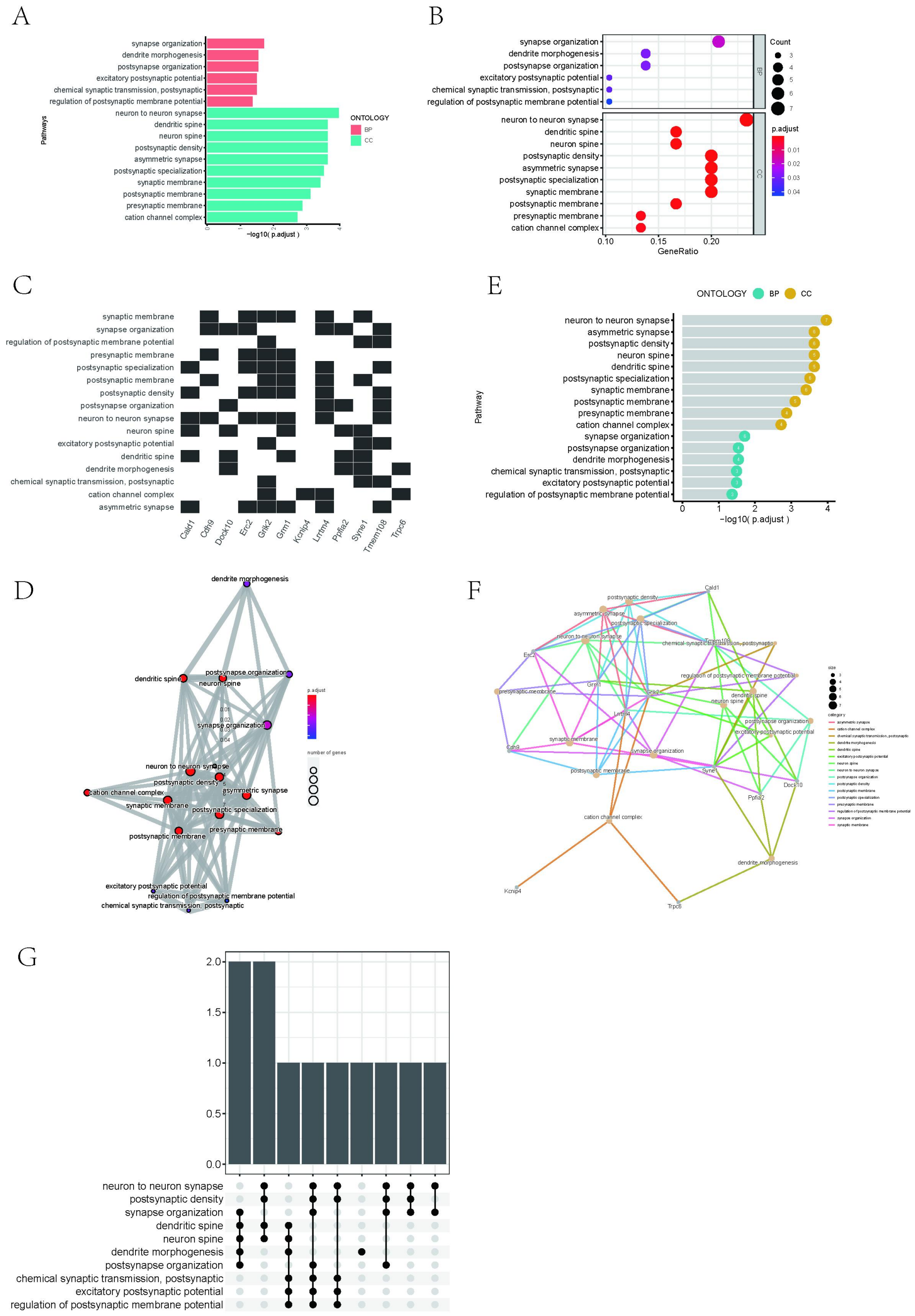

Figure S26. GO analysis of Module 2 functional categories (related to Figure 5)
(A) Enrichment of BP, CC, and MF pathways in Module 2. The horizontal coordinate was -log10(p.adjust) and the vertical coordinate was GO Term. The longer column stands for the more significant enrichment.
(B) Bubble diagram of BP, CC, and MF enrichment in Module 2, indicating the relationship between GeneRatio and GO Term.
(C) Gene enrichment of each pathway, where squares indicated the gene belonging to the pathway.
(D) Based on the network diagram of pathway gene sharing and similarity, the pathways with high coincidence degrees were clustered together, which facilitated the identification of functional modules.
(E) The number value in the circle showed the number of genes enriched in each pathway of Module 2. The longer the lollipop column stood for the more significant the enrichment. (F) The map of the association network between genes and pathways showed the mole
cules shared between each pathway.
(G) The unique or shared genes of each pathway. The black dots on the lines between pathways represent genes shared by the pathways, and the top bar represents the number of genes unique or shared.

Figure S27

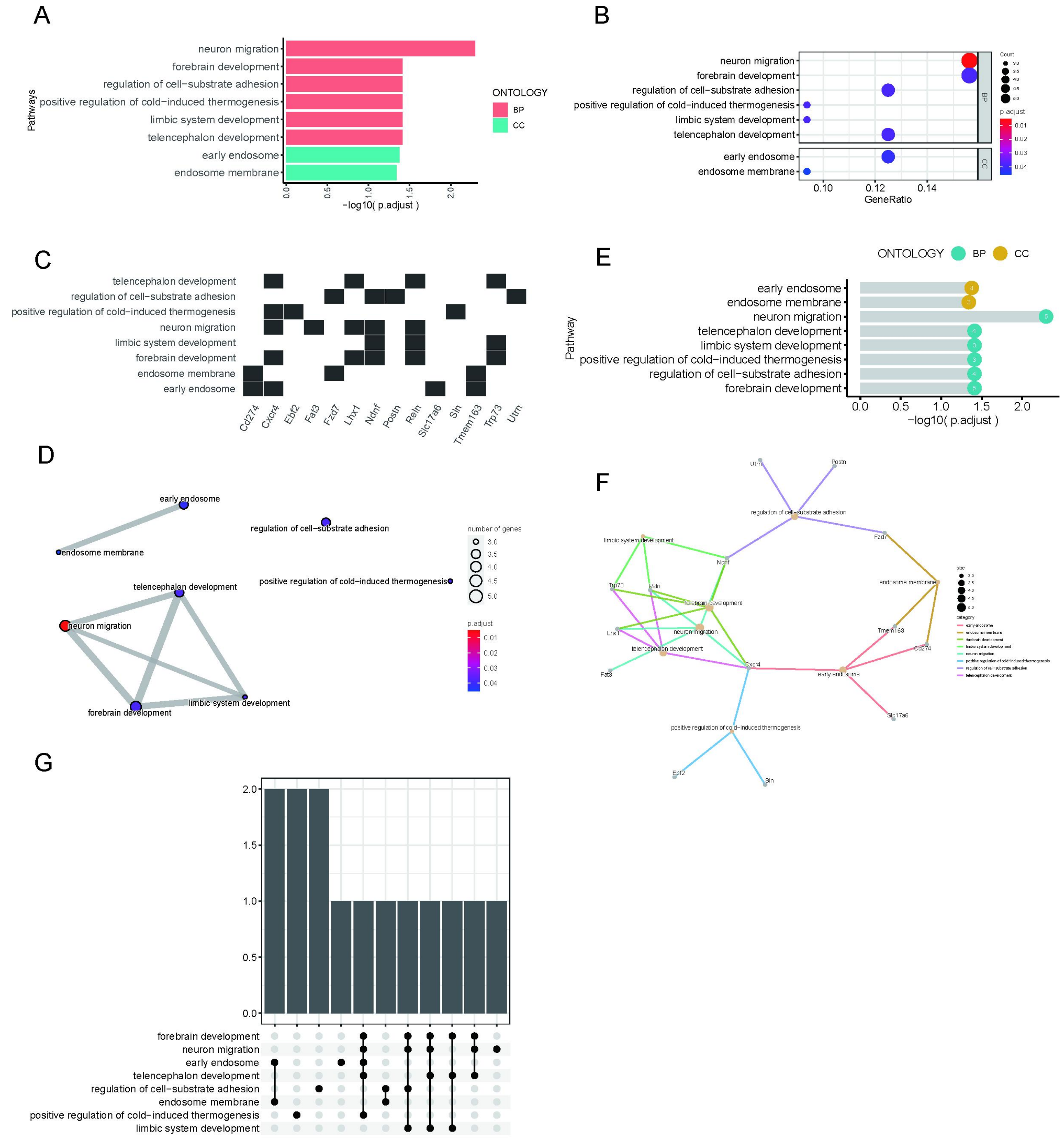

Figure S27. GO analysis of Module 3 functional categories (related to Figure 5)

(A) Enrichment of BP, CC, and MF pathways in Module 3. The horizontal coordinate was -log10(p.adjust) and the vertical coordinate was GO Term. The longer column stands for the more significant enrichment.

(B) Bubble diagram of BP, CC, and MF enrichment in Module 3, indicating the relationship between GeneRatio and GO Term.

(C) Gene enrichment of each pathway, where squares indicated the gene belonging to the pathway.

(D) Based on the network diagram of pathway gene sharing and similarity, the pathways with high coincidence degrees were clustered together, which facilitated the identification of functional modules.

(E) The number value in the circle showed the number of genes enriched in each pathway of Module 3. The longer the lollipop column stood for the more significant the enrichment.

(F) The map of the association network between genes and pathways showed the molecules shared between each pathway.

(G) The unique or shared genes of each pathway. The black dots on the lines between pathways represent genes shared by the pathways, and the top bar represents the number of genes unique or shared.

Figure S28

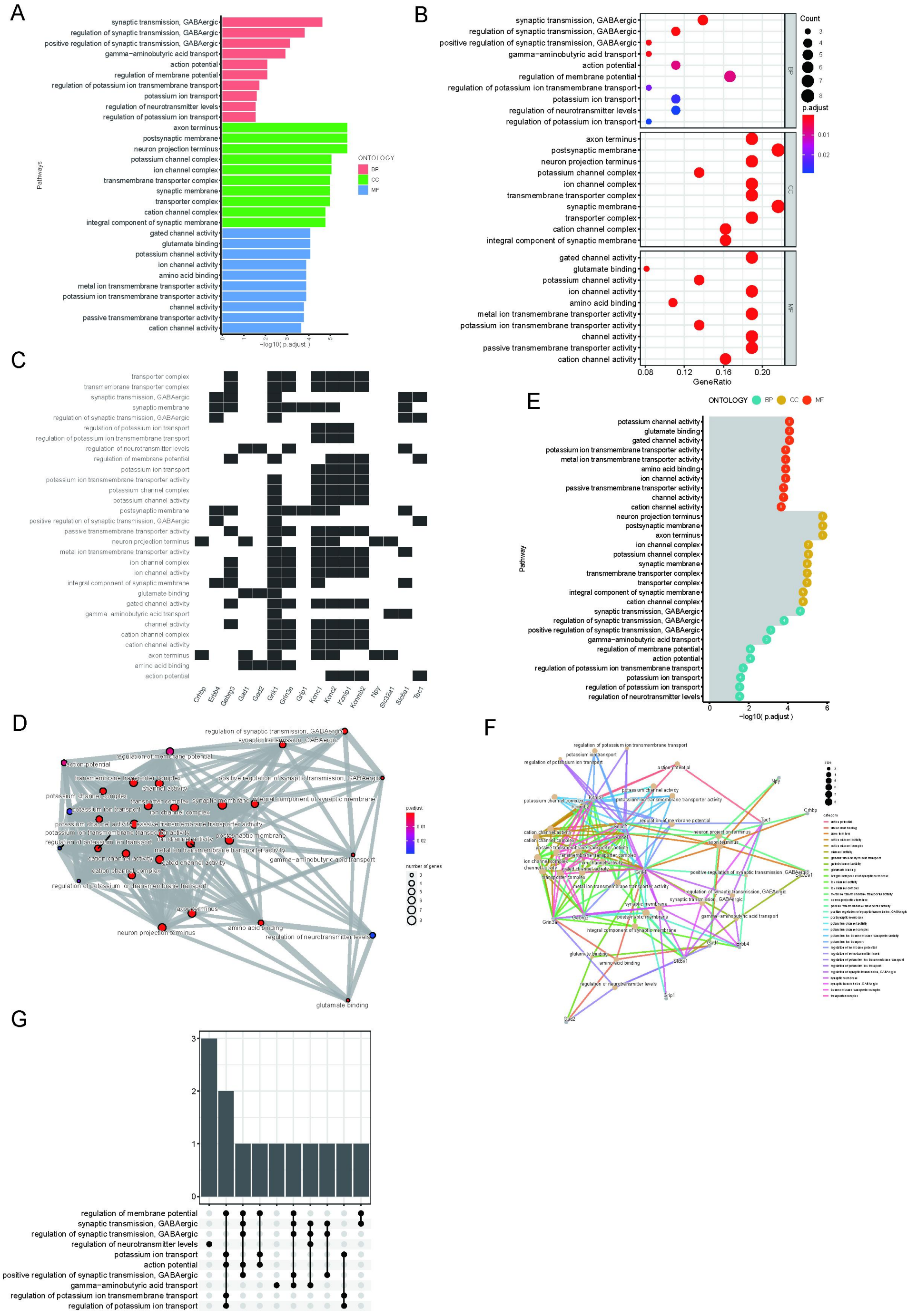

Figure S28. GO analysis of Module 4 functional categories (related to Figure 5)

(A) Enrichment of BP, CC, and MF pathways in Module 4. The horizontal coordinate was -log10(p.adjust) and the vertical coordinate was GO Term. The longer column stands for the more significant enrichment.

(B) Bubble diagram of BP, CC, and MF enrichment in Module 4, indicating the relationship between GeneRatio and GO Term.

(C) Gene enrichment of each pathway, where squares indicated the gene belonging to the pathway.

(D) Based on the network diagram of pathway gene sharing and similarity, the pathways with high coincidence degrees were clustered together, which facilitated the identification of functional modules.

(E) The number value in the circle showed the number of genes enriched in each pathway of Module 4. The longer the lollipop column stood for the more significant the enrichment.

(F) The map of the association network between genes and pathways showed the molecules shared between each pathway.

(G) The unique or shared genes of each pathway. The black dots on the lines between pathways represent genes shared by the pathways, and the top bar represents the number of genes unique or shared.

Figure S29

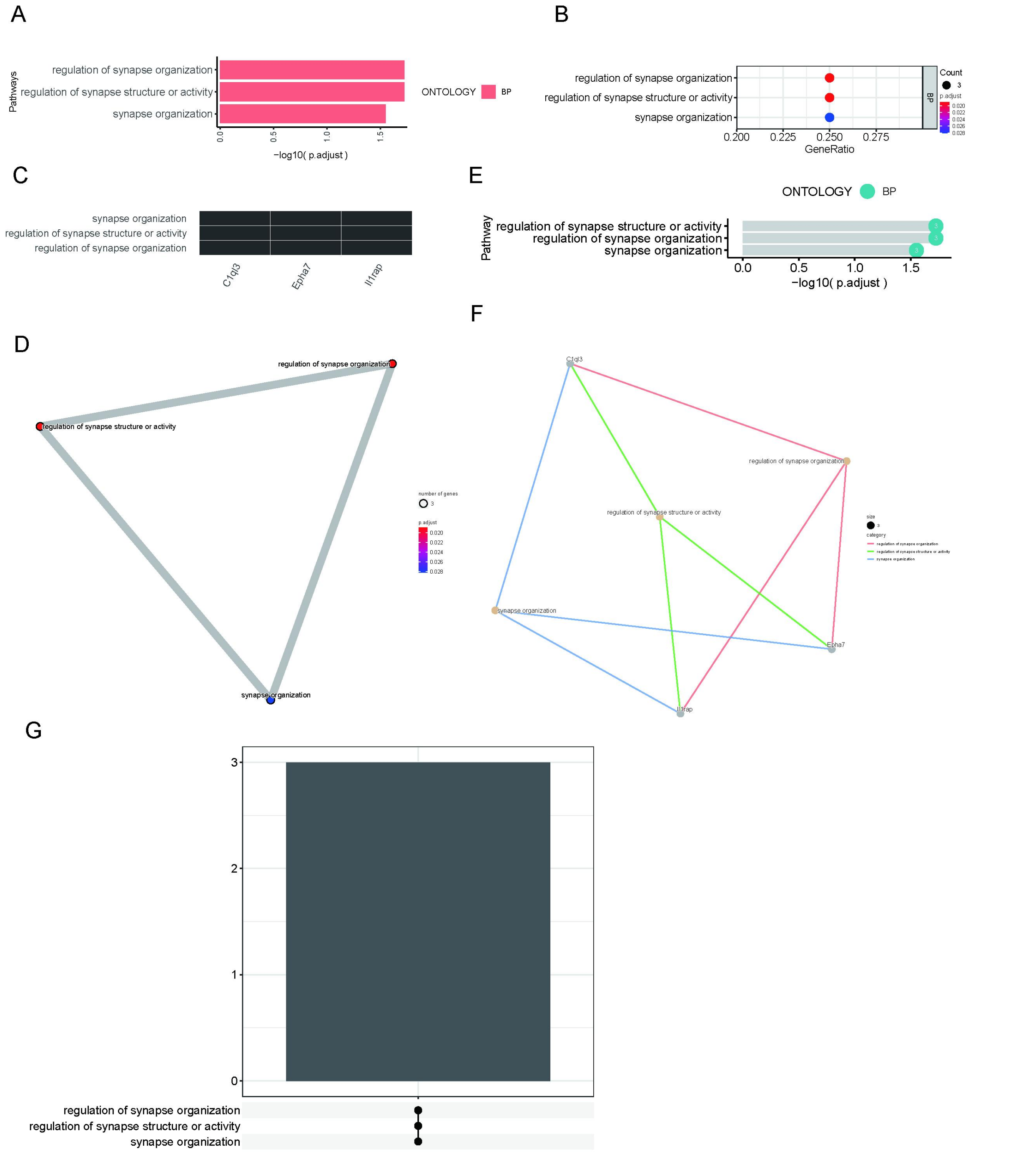

Figure S29. GO analysis of Module 5 functional categories (related to Figure 5)
(A) Enrichment of BP, CC, and MF pathways in Module 5. The horizontal coordinate was -log10(p.adjust) and the vertical coordinate was GO Term. The longer column stands for the more significant enrichment.
(B) Bubble diagram of BP, CC, and MF enrichment in Module 5, indicating the relationship between GeneRatio and GO Term.
(C) Gene enrichment of each pathway, where squares indicated the gene belonging to the pathway.
(D) Based on the network diagram of pathway gene sharing and similarity, the pathways with high coincidence degrees were clustered together, which facilitated the identification of functional modules.
(E) The number value in the circle showed the number of genes enriched in each pathway of Module 5. The longer the lollipop column stood for the more significant the enrichment.
(F) The map of the association network between genes and pathways showed the molecules shared between each pathway.
(G) The unique or shared genes of each pathway. The black dots on the lines between pathways represent genes shared by the pathways, and the top bar represents the number of genes unique or shared.

Figure S30

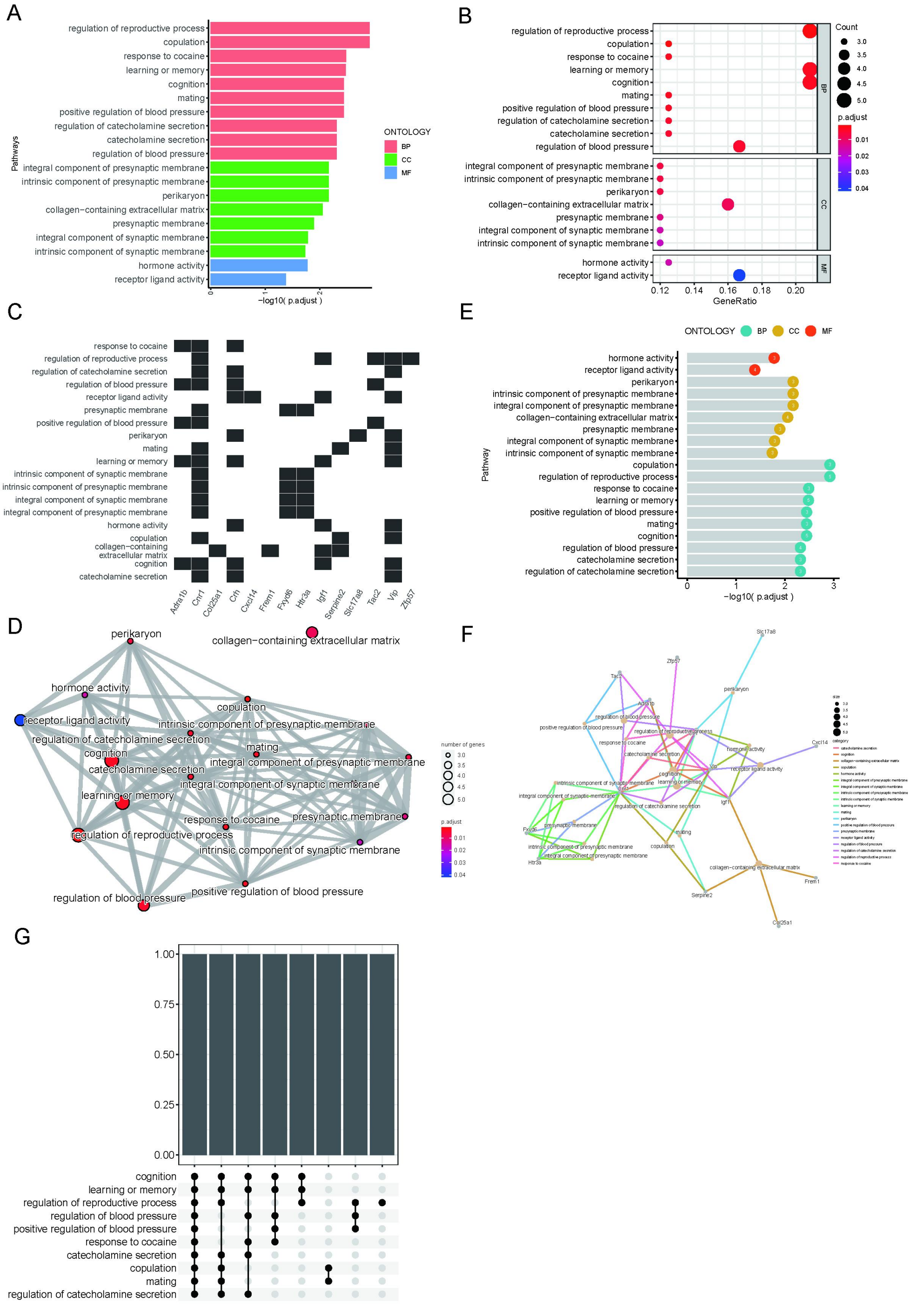

Figure S30. GO analysis of Module 3 functional categories (related to Figure 5)

(A) Enrichment of BP, CC, and MF pathways in Module 6. The horizontal coordinate was -log10(p.adjust) and the vertical coordinate was GO Term. The longer column stands for the more significant enrichment.

(B) Bubble diagram of BP, CC, and MF enrichment in Module 6, indicating the relationship between GeneRatio and GO Term.

(C) Gene enrichment of each pathway, where squares indicated the gene belonging to the pathway.

(D) Based on the network diagram of pathway gene sharing and similarity, the pathways with high coincidence degrees were clustered together, which facilitated the identification of functional modules.

(E) The number value in the circle showed the number of genes enriched in each pathway of Module 6. The longer the lollipop column stood for the more significant the enrichment.

(F) The map of the association network between genes and pathways showed the molecules shared between each pathway.

(G) The unique or shared genes of each pathway. The black dots on the lines between pathways represent genes shared by the pathways, and the top bar represents the number of genes unique or shared.

Figure S31

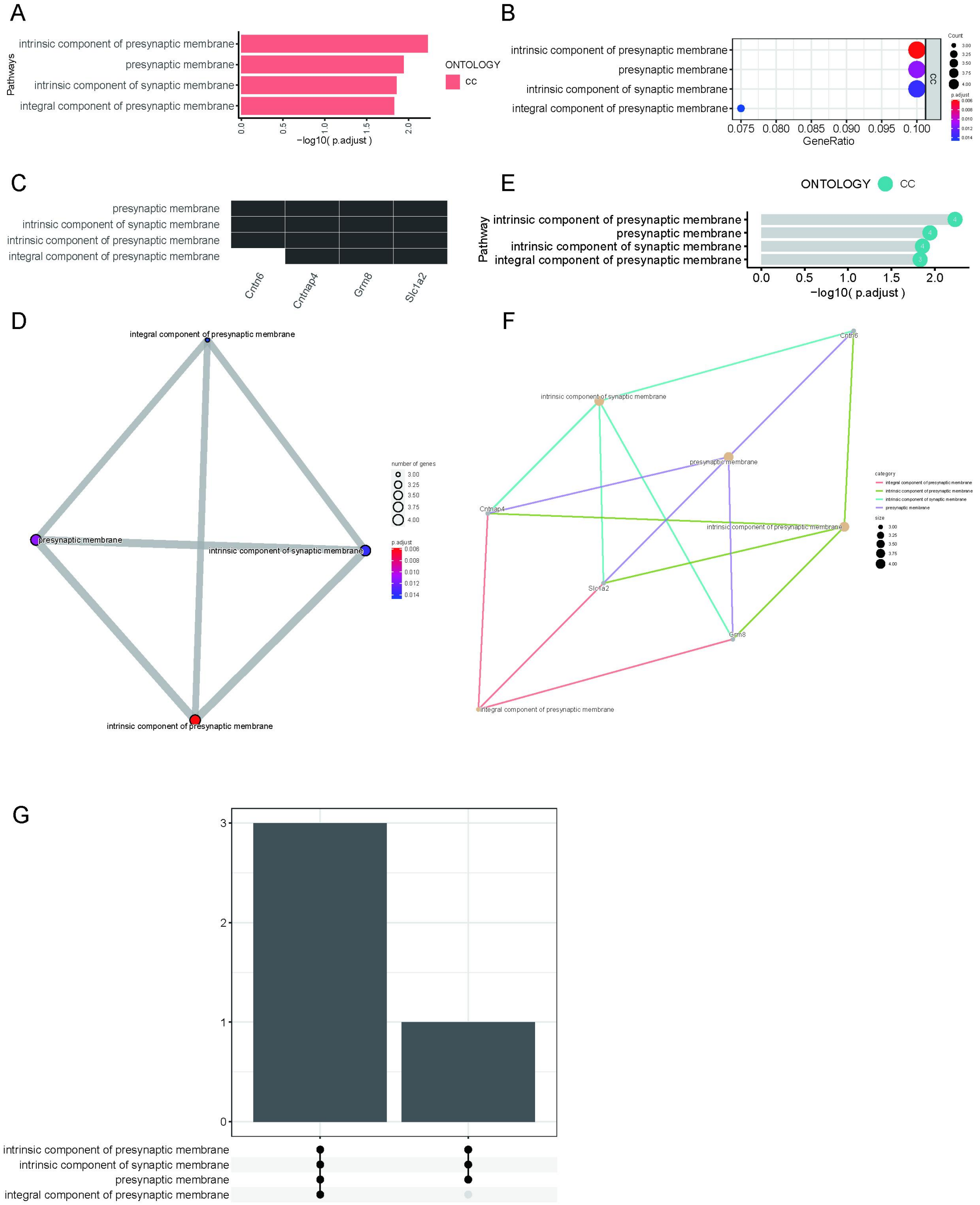

Figure S31. GO analysis of Module 7 functional categories (related to Figure 5)

(A) Enrichment of BP, CC, and MF pathways in Module 7. The horizontal coordinate was -log10(p.adjust) and the vertical coordinate was GO Term. The longer column stands for the more significant enrichment.

(B) Bubble diagram of BP, CC, and MF enrichment in Module 7, indicating the relationship between GeneRatio and GO Term.

(C) Gene enrichment of each pathway, where squares indicated the gene belonging to the pathway.

(D) Based on the network diagram of pathway gene sharing and similarity, the pathways with high coincidence degrees were clustered together, which facilitated the identification of functional modules.

(E) The number value in the circle showed the number of genes enriched in each pathway of Module 7. The longer the lollipop column stood for the more significant the enrichment.

(F) The map of the association network between genes and pathways showed the molecules shared between each pathway.

(G) The unique or shared genes of each pathway. The black dots on the lines between pathways represent genes shared by the pathways, and the top bar represents the number of genes unique or shared.

Figure S32

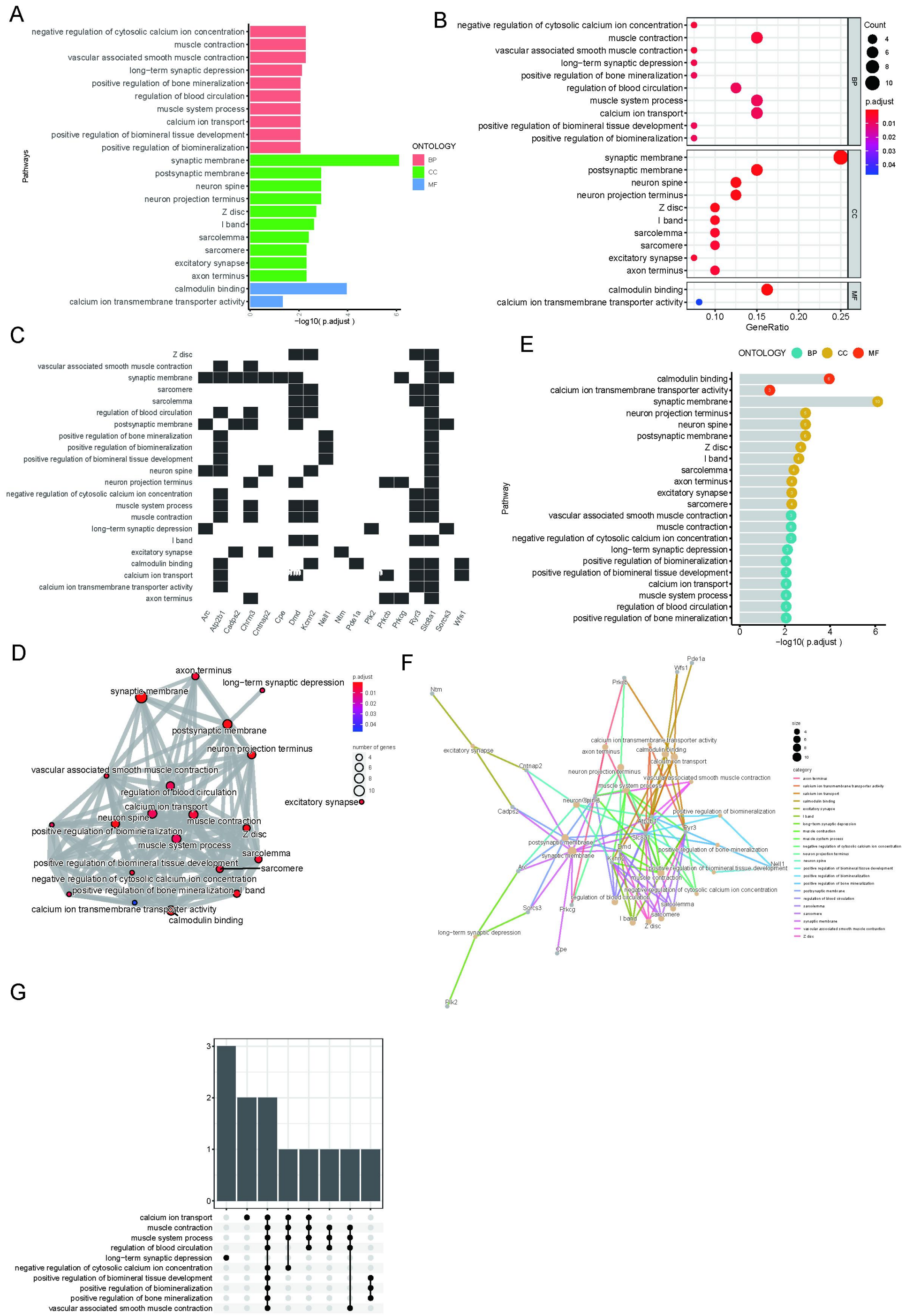

Figure S32. GO analysis of Module 8 functional categories (related to Figure 5)

(A) Enrichment of BP, CC, and MF pathways in Module 8. The horizontal coordinate was -log10(p.adjust) and the vertical coordinate was GO Term. The longer column stands for the more significant enrichment.

(B) Bubble diagram of BP, CC, and MF enrichment in Module 8, indicating the relationship between GeneRatio and GO Term.

(C) Gene enrichment of each pathway, where squares indicated the gene belonging to the pathway.

(D) Based on the network diagram of pathway gene sharing and similarity, the pathways with high coincidence degrees were clustered together, which facilitated the identification of functional modules.

(E) The number value in the circle showed the number of genes enriched in each pathway of Module 8. The longer the lollipop column stood for the more significant the enrichment.

(F) The map of the association network between genes and pathways showed the molecules shared between each pathway.

(G) The unique or shared genes of each pathway. The black dots on the lines between pathways represent genes shared by the pathways, and the top bar represents the number of genes unique or shared.

Figure S33

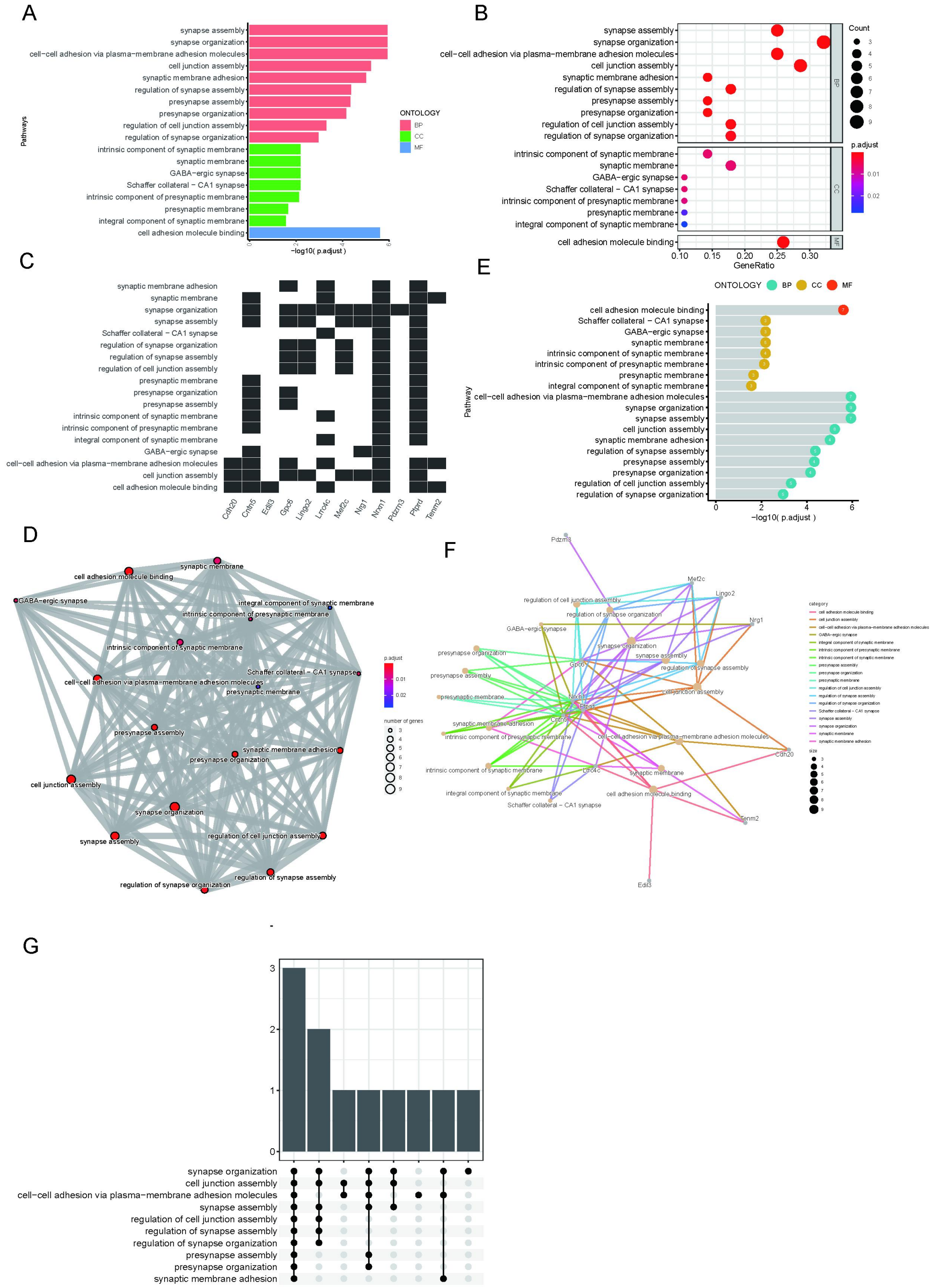

Figure S33. GO analysis of Module 3 functional categories (related to Figure 5)

(A) Enrichment of BP, CC, and MF pathways in Module 9. The horizontal coordinate was -log10(p.adjust) and the vertical coordinate was GO Term. The longer column stands for the more significant enrichment.

(B) Bubble diagram of BP, CC, and MF enrichment in Module 9, indicating the relationship between GeneRatio and GO Term.

(C) Gene enrichment of each pathway, where squares indicated the gene belonging to the pathway.

(D) Based on the network diagram of pathway gene sharing and similarity, the pathways with high coincidence degrees were clustered together, which facilitated the identification of functional modules.

(E) The number value in the circle showed the number of genes enriched in each pathway of Module 9. The longer the lollipop column stood for the more significant the enrichment.

(F) The map of the association network between genes and pathways showed the molecules shared between each pathway.

(G) The unique or shared genes of each pathway. The black dots on the lines between pathways represent genes shared by the pathways, and the top bar represents the number of genes unique or shared.

Figure S34

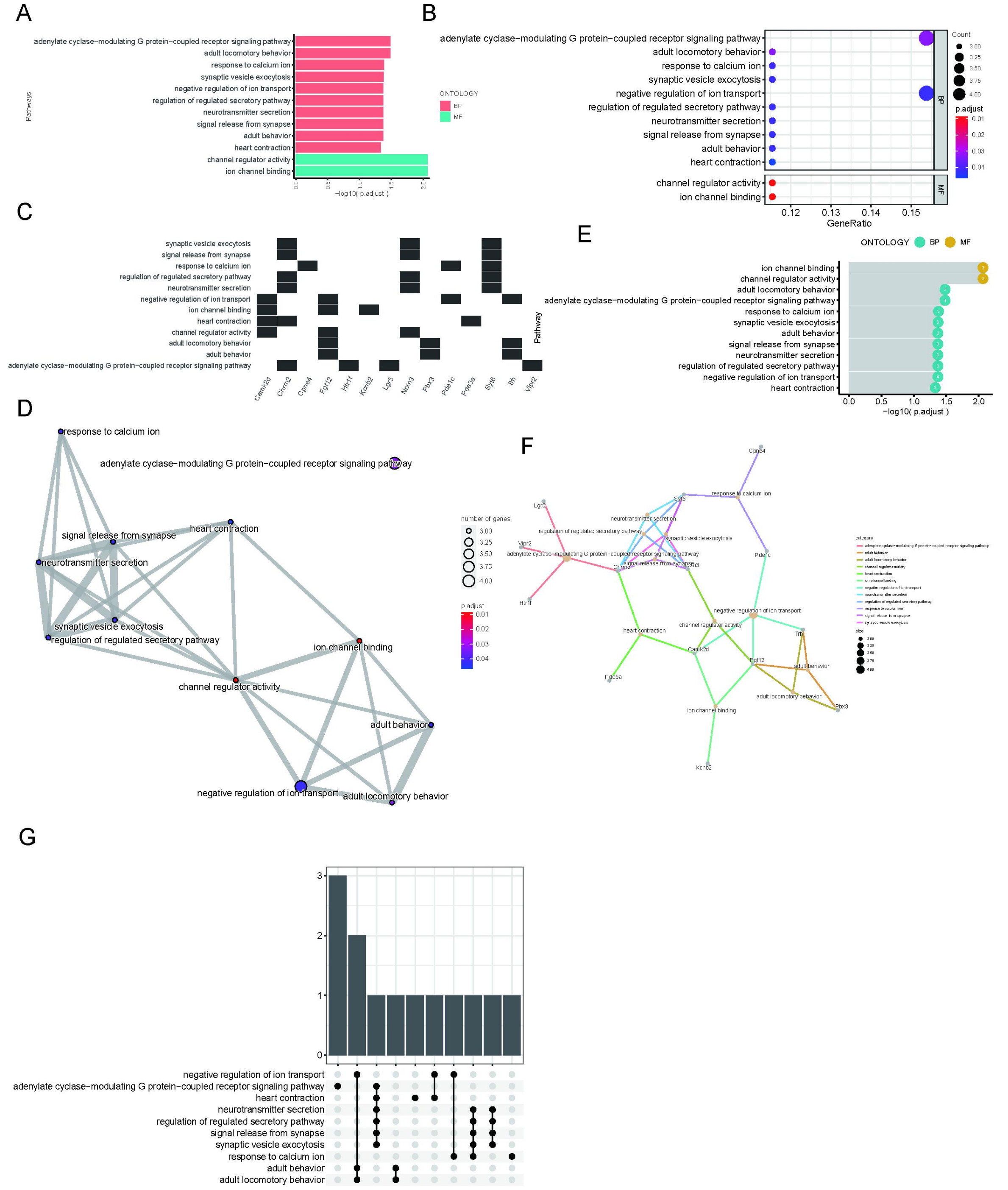

Figure S34. GO analysis of Module10 functional categories (related to Figure 5)

(A) Enrichment of BP, CC, and MF pathways in Module10. The horizontal coordinate was -log10(p.adjust) and the vertical coordinate was GO Term. The longer column stands for the more significant enrichment.

(B) Bubble diagram of BP, CC, and MF enrichment in Module 10, indicating the relationship between GeneRatio and GO Term.

(C) Gene enrichment of each pathway, where squares indicated the gene belonging to the pathway.

(D) Based on the network diagram of pathway gene sharing and similarity, the pathways with high coincidence degrees were clustered together, which facilitated the identification of functional modules.

(E) The number value in the circle showed the number of genes enriched in each pathway of Module 10. The longer the lollipop column stood for the more significant the enrichment.

(F) The map of the association network between genes and pathways showed the molecules shared between each pathway.

(G) The unique or shared genes of each pathway. The black dots on the lines between pathways represent genes shared by the pathways, and the top bar represents the number of genes unique or shared.

Figure S35

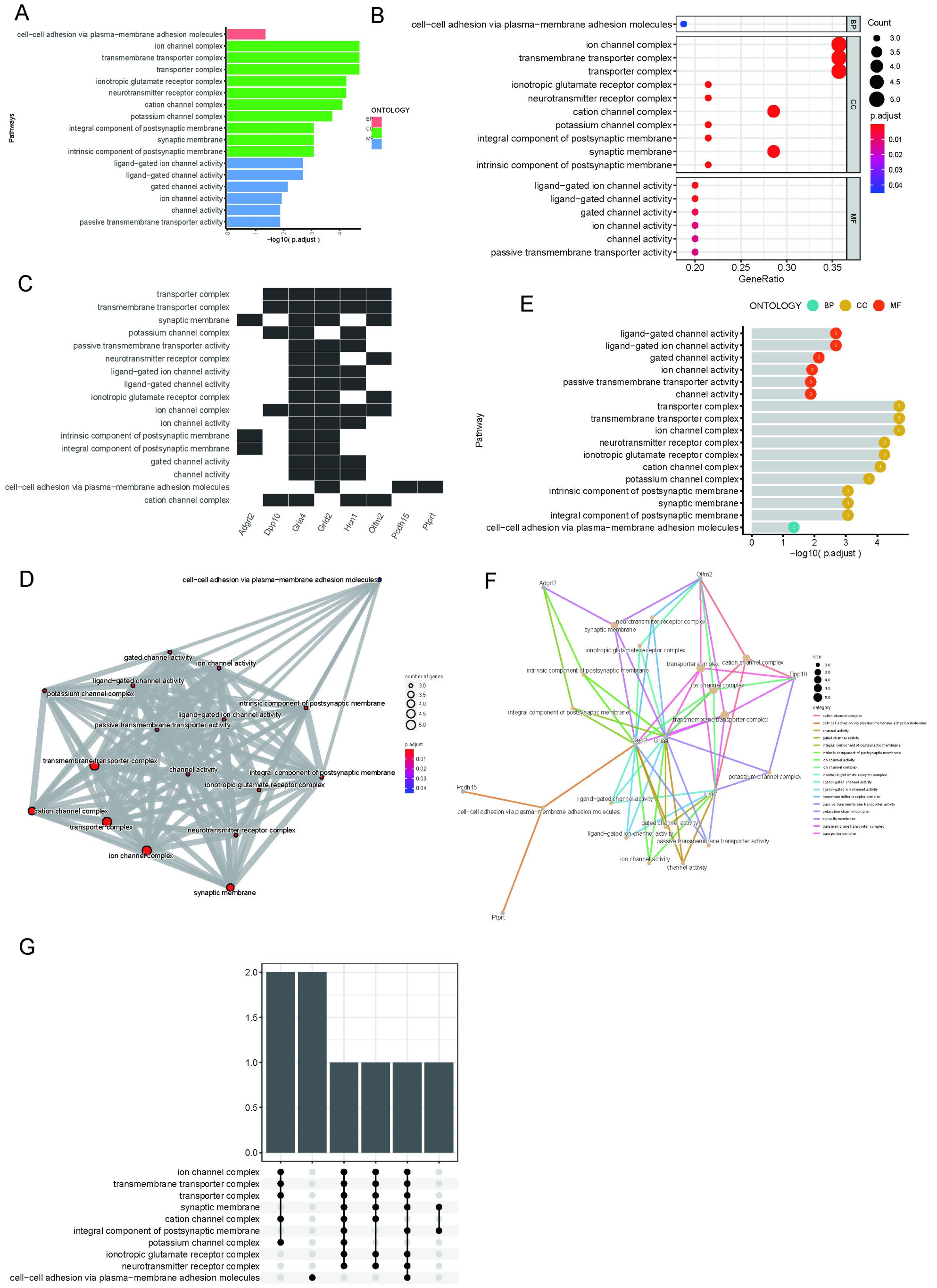

Figure S35. GO analysis of Module 12 functional categories (related to Figure 5)

(A) Enrichment of BP, CC, and MF pathways in Module12. The horizontal coordinate was -log10(p.adjust) and the vertical coordinate was GO Term. The longer column stands for the more significant enrichment.

(B) Bubble diagram of BP, CC, and MF enrichment in Module 12, indicating the relationship between GeneRatio and GO Term.

(C) Gene enrichment of each pathway, where squares indicated the gene belonging to the pathway.

(D) Based on the network diagram of pathway gene sharing and similarity, the pathways with high coincidence degrees were clustered together, which facilitated the identification of functional modules.

(E) The number value in the circle showed the number of genes enriched in each pathway of Module 12. The longer the lollipop column stood for the more significant the enrichment.

(F) The map of the association network between genes and pathways showed the molecules shared between each pathway.

(G) The unique or shared genes of each pathway. The black dots on the lines between pathways represent genes shared by the pathways, and the top bar represents the number of genes unique or shared.

Figure S36

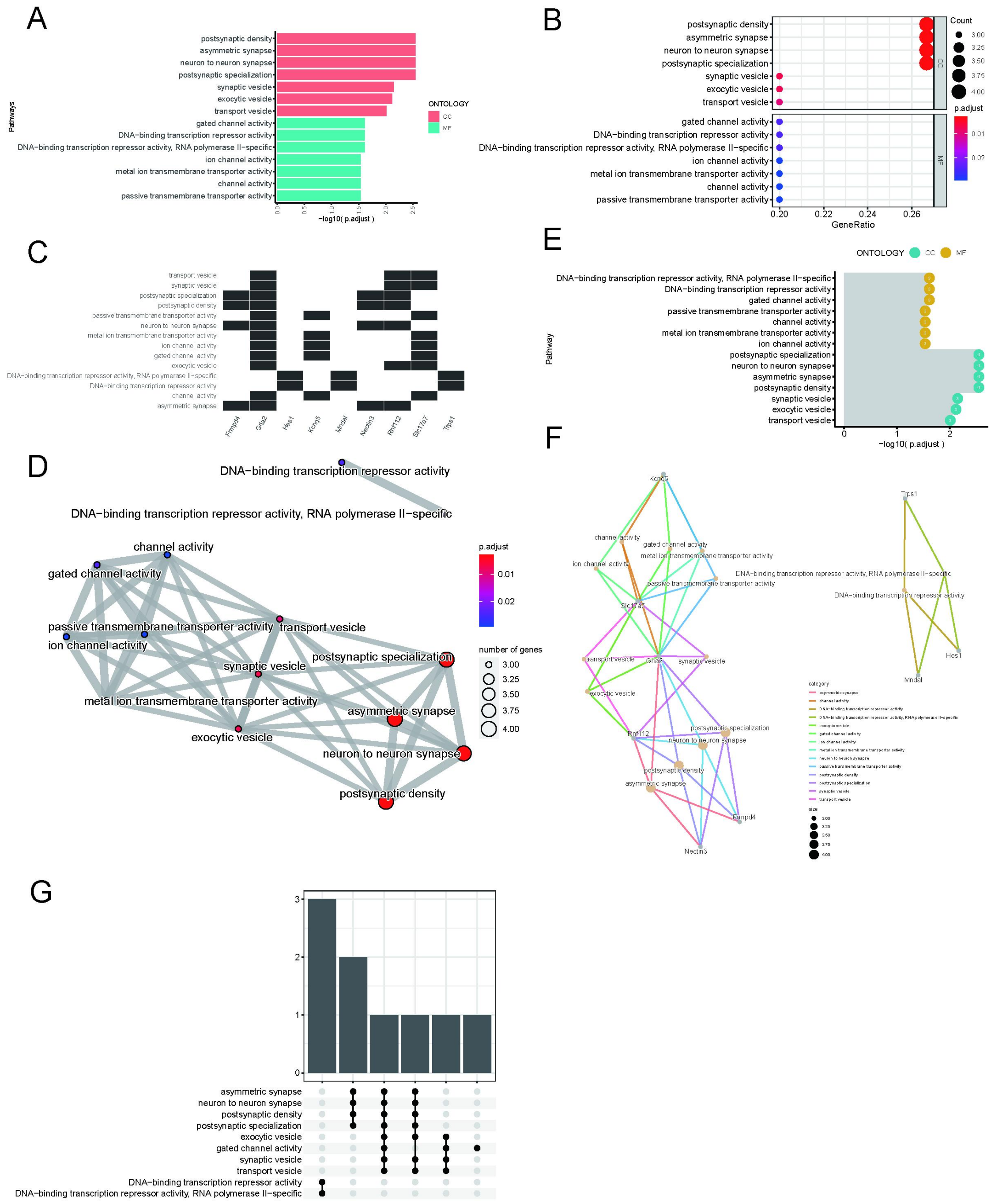

Figure S36. GO analysis of Module 13 functional categories (related to Figure 5)
(A) Enrichment of BP, CC, and MF pathways in Module 13. The horizontal coordinate was -log10(p.adjust) and the vertical coordinate was GO Term. The longer column stands for the more significant enrichment.
(B) Bubble diagram of BP, CC, and MF enrichment in Module 13, indicating the relationship between GeneRatio and GO Term.
(C) Gene enrichment of each pathway, where squares indicated the gene belonging to the pathway.
(D) Based on the network diagram of pathway gene sharing and similarity, the pathways with high coincidence degrees were clustered together, which facilitated the identification of functional modules.
(E) The number value in the circle showed the number of genes enriched in each pathway of Module 13. The longer the lollipop column stood for the more significant the enrichment.
(F) The map of the association network between genes and pathways showed the molecules shared between each pathway.
(G) The unique or shared genes of each pathway. The black dots on the lines between pathways represent genes shared by the pathways, and the top bar represents the number of genes unique or shared.

Figure S37

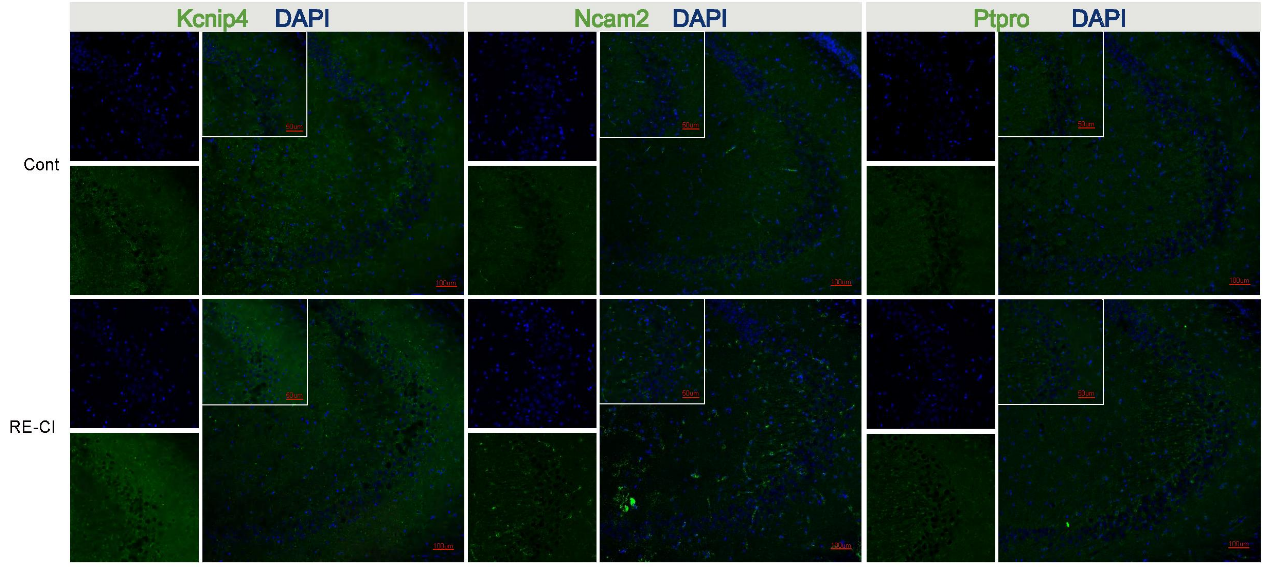

Figure S37. Representative immunofluorescence images of Kcnip4, Ncam2, and Ptpro and co-staining of DAPI in the CA3 region of the mouse hippocampus (related to Figure 5)

Figure S38

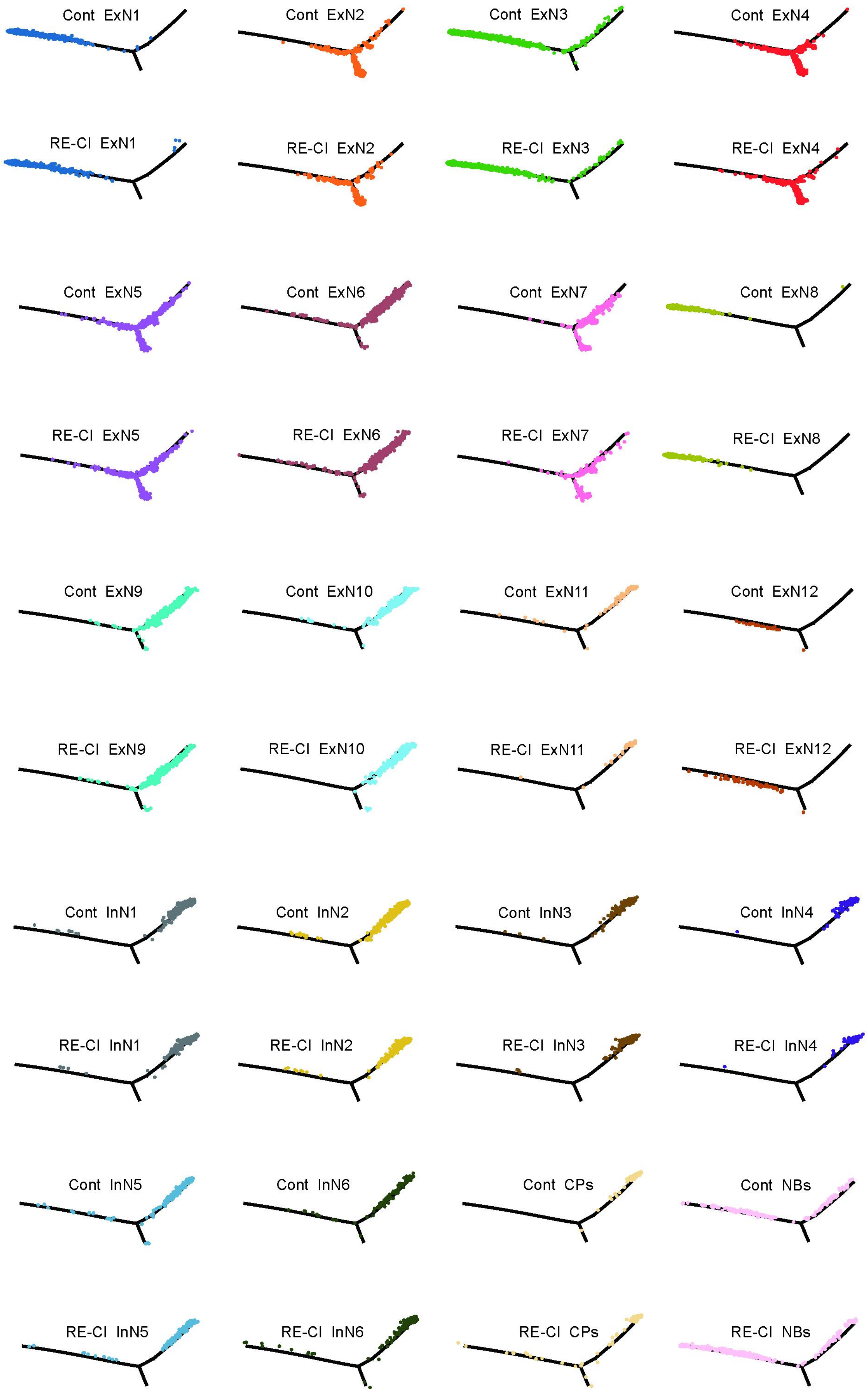

Figure S38. The quasi-temporal analysis of each neuron subpopulation distribution of control and RE-CI groups (related to Figure 6)

Figure S39

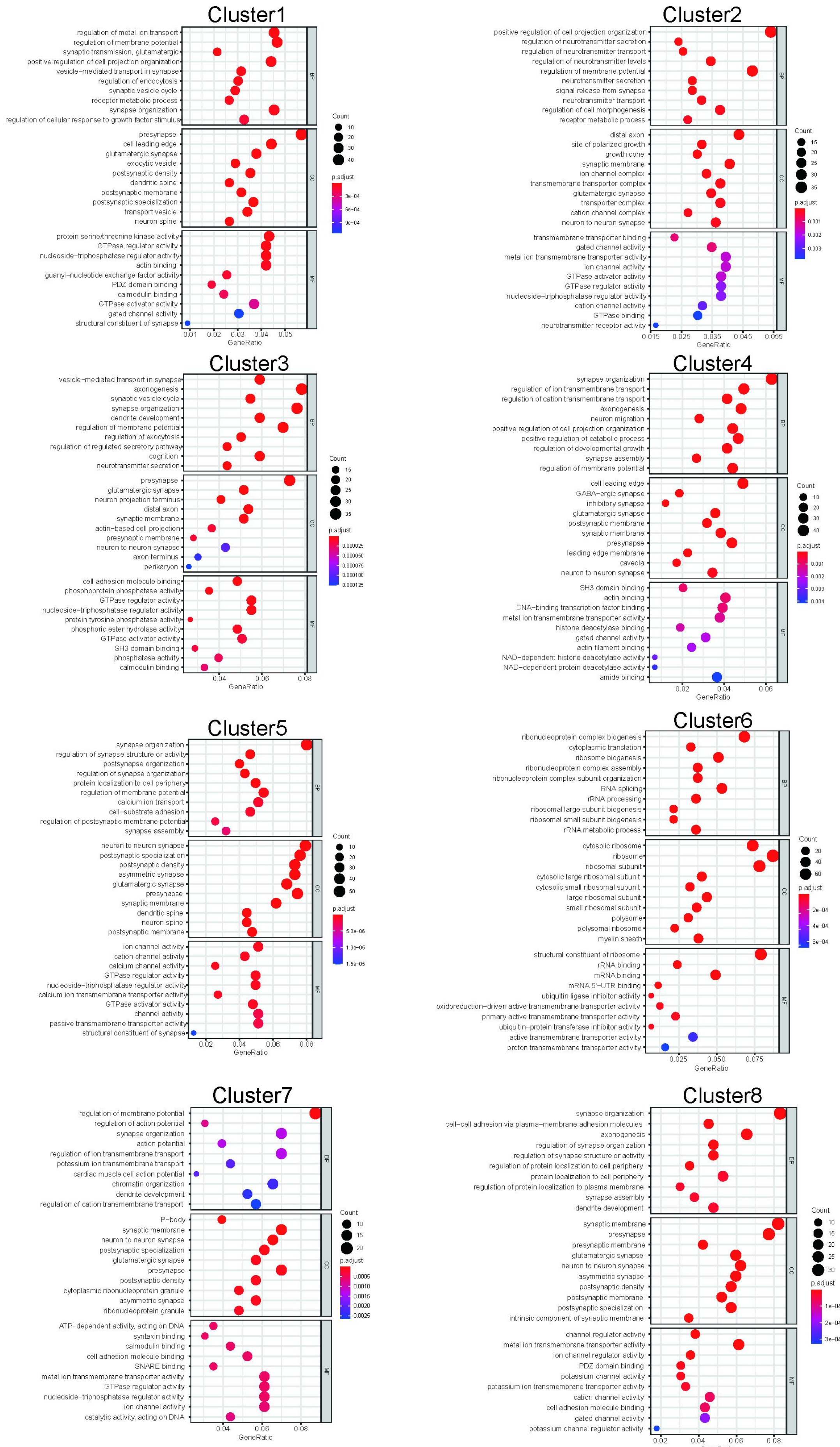

Figure S39. Dotplot showed the GO enrichment paths of Cluster 1-8 (related to Figure 6) The horizontal coordinate was GeneRatio, and the vertical coordinate was GO Term. The color of the circle represented the value of p.adjust, and the size of the circle represented the number of differential genes.

Figure S40

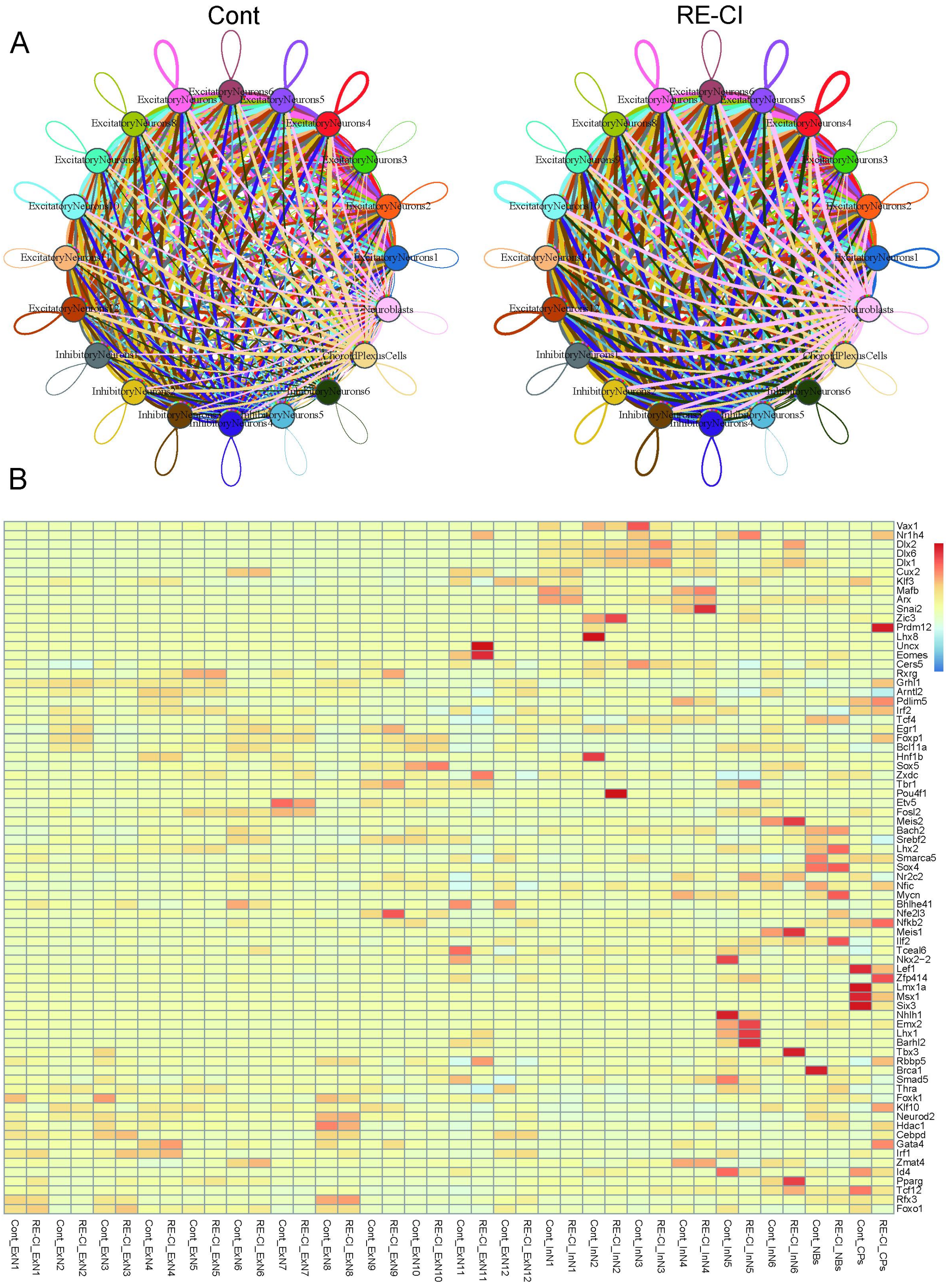

Figure S40. (related to Figure 7)
(A) Interaction network diagram between neuron subsets, where the network nodes were different neuron subsets. The thickness of the network edges was the total number of ligand and receptor pairs, and the color of the lines was consistent with the type of ligand cells.
(B) Average expression heat maps of Top75 transcription factors in Control and RE-CI groups, respectively.

Figure S41

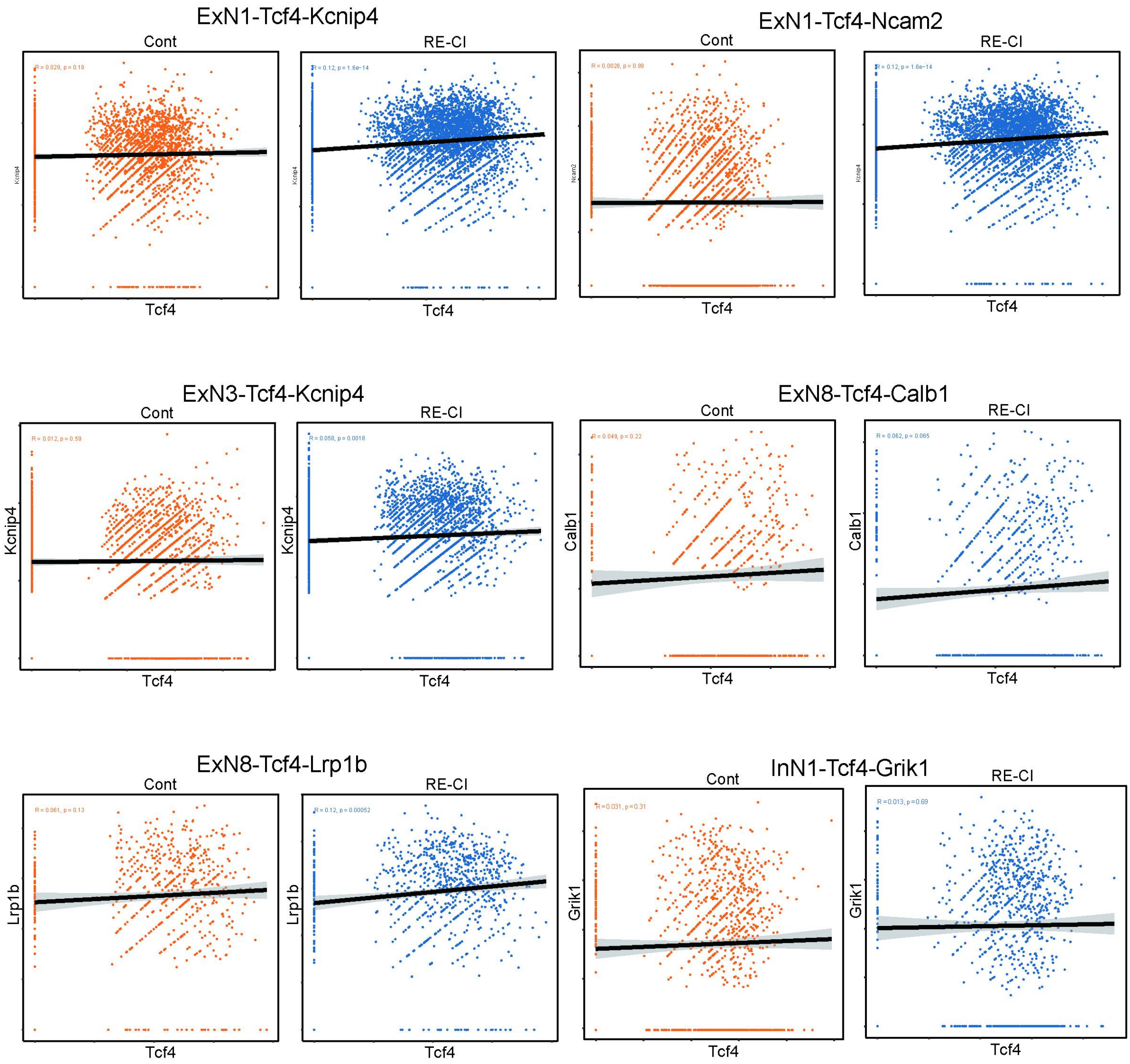

Figure S41. Correlation analysis of transcription factor Tcf4 with cognitive impairment genes in ExN1, 3, 8, and InN1 subgroups (related to Figure 7)

Figure S42

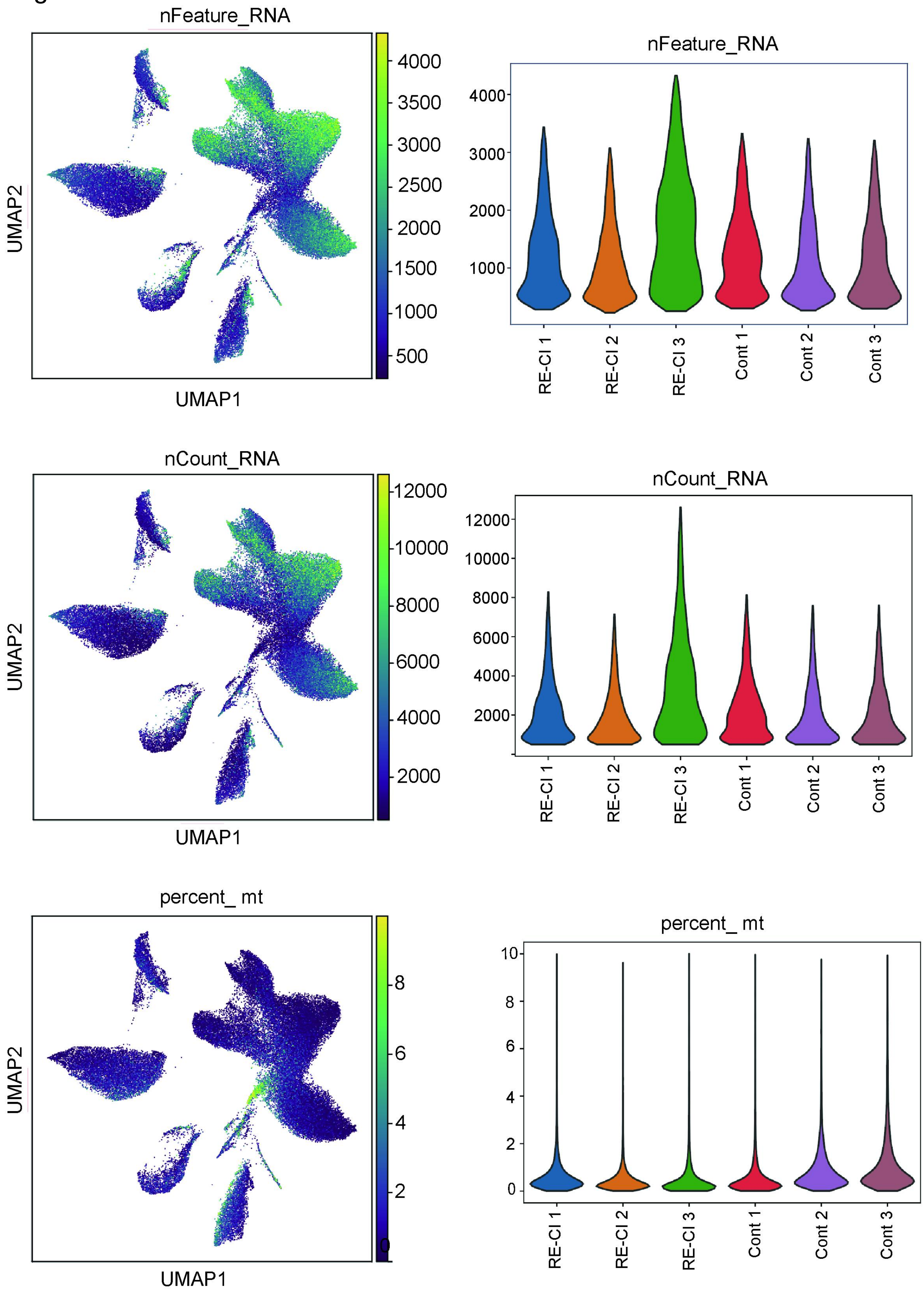

Figure S42. QC of the snRNAseq data (related to method details)

Table S1. Number of nuclei in 9 types of hippocampal cells and Number of cell nuclei in 20 neuronal subpopulations (related to Figure 3)

Number of nuclei in 9 types of hippocampal cells

Cluster	RE-CI	Cont
ACs	1218	2338
Ecs	134	124
MPs	107	52
Meningeal	425	411
Microglial	1345	1069
Neurons	21120	19187
OPCs	1347	1465
OLGs	5779	3563
T	34	8

Number of cell nuclei in 20 neuronal subpopulations

Cluster	RE-CI	Cont
ExN1	4040	2191
ExN2	2815	2426
ExN3	2868	2094
ExN4	1616	1692
ExN5	1051	1039
ExN6	914	1174
ExN7	741	871
ExN8	877	610
ExN9	566	802
ExN10	449	447
ExN11	51	80
ExN12	67	31
InN1	904	1067
InN2	440	1100
InN3	562	559
InN4	321	368
InN5	184	184
InN6	248	105
CPs	60	86
NBs	420	197

Table S2. Nonstandard Abbreviations and Acronyms (related to Figure 3)

Hippocampal cell type	abbreviation
Astrocytes	ACs
Endothelial cells	ECs
Mononuclear phagocyte system	MPs
MeningealCells	Meningeal
MicroglialCells	Microglial
Neurons	Neurons
Oligodendrocyte precursor cells	OPCs
Oligodendrocytes	OLGs
Tcells	T
**Neuronal subpopulation**	
Neuroblasts	NBs
ChoroidPlexusCells	CPs
InhibitoryNeurons	InN
ExcitatoryNeurons	ExN

Table S3. Differential genes of 9 types of hippocampal cells and Differential genes in 20 subtypes of hippocampal neurons (related to Figure 3)

Marker gene	Cell type	Marker gene	Cell type
Aldoc	ACs	Trpc6	ExN3
Aqp4	ACs	Gm32647	ExN4
Slc1a3	ACs	Mndal	ExN4
Cldn5	Ecs	Dcn	ExN5
Pecam1	ECs	Nnat	ExN5
Vwf	ECs	Gpc6	ExN6
Col1a2	Meningeal	Bglap3	ExN6
Slc6a13	Meningeal	Fn1	ExN7
Slc47a1	Meningeal	Ndst3	ExN7
Cx3cr1	Microglial	Ahcyl2	ExN8
P2ry12	Microglial	Slc26a10	ExN8
Tmem119	Microglial	Ctgf	ExN9
Ccr2	MPs	Garnl3	ExN9
Cd163	MPs	Cntn6	ExN10
Cd209a	MPs	Pamr1	ExN10
Ly6c2	MPs	Postn	ExN11
Lyz2	MPs	Fzd7	ExN11
Mrc1	MPs	Atp2b1	ExN12
Meg3	Neurons	Iqgap2	ExN12
Snap25	Neurons	Sst	InN1
Syt1	Neurons	Grik1	InN1
Mbp	OLGs	Prkcd	InN2
Mobp	OLGs	Arhgap6	InN2
Mog	OLGs	Cnr1	InN3
Bcan	OPCs	Adarb2	InN3
Pdgfra	OPCs	Erbb4	InN4
Vcan	OPCs	Hapln1	InN4
Cd2	T	Fgf13	InN4
Cd3d	T	Lamp5	InN4
Trac	T	Dach1	InN5
Trbc2	T	Trp73	InN5
Stxbp6	ExN1	Pbx3	InN6
Cdh9	ExN1	Pcbp3	InN6
Galntl6	ExN2	Ttr	CPs
Epha6	ExN2	Sox11	NBs
C1ql2	ExN3	Igfbpl1	NBs

Table S5. Markers of different regions of hippocampal tissue in different species (related to Figure 8)

	Different zones of the hippocampus	Marker	
Aged humans	CA1	SATB1	MPPED1
	CA2	STMN2	RGS4
	CA3	CPNE4	
	Granule cell	BCL11b	
	GABAergic	GAD1	GAD2
	IMMN	NNAT	
Macaques	CA1	MPPED1	
	CA2	SATB2	PCP4
	CA3	CPNE4	NETO1
	Granule cell	BCL11b	
	GABAergic	GAD1	GAD2
	NB/IMMN	PROX1	SEMA3C
Mouse	CA	SV2B	
	CA1	SATB2	
	CA2、CA3	PFKP	
	CA1、CA3	TYRO3	
	Granule cell	MAML2	
	GABAergic	GAD1	GAD2
	Subiculum	FN1	

Table S6. Genes associated with memory impairment in specific cell subpopulations (related to Figure 4)

Names	Scores	logFC	p_vals	p_vals_adj	pct_nz_group
Zfpm2	67.55131	1.3457348	0	0	0.681110576
Ncam2	64.69853	1.2704473	0	0	0.783501846
Kcnip4	71.412766	1.145527	0	0	0.982025357
Pex5l	68.36792	1.4011596	0	0	0.782102652
Zfpm2	67.55131	1.3457348	0	0	0.681110576
Ncam2	7.703911	0.43544838	1.32E-14	3.59E-14	0.462313583
Kcnip4	71.412766	1.145527	0	0	0.982025357
Nnat	64.46378	2.2736225	0	0	0.772727273
Pex5l	40.110443	1.3334875	0	0	0.818858561
Calb1	41.562603	1.2810899	0	0	0.550773369
Lrp1b	32.959755	1.028053	3.07E-238	2.28E-235	0.939475454
Grm3	28.980465	1.1943758	1.16E-184	6.82E-182	0.407163743
Grm3	22.149115	0.96406966	1.06E-108	1.62E-106	0.404017857
Sema5a	7.271516	1.2089239	3.55E-13	5.19E-11	0.458015267
Grik1	75.228195	2.5348983	0	0	0.725012684
Grik1	45.696804	2.211641	0	0	0.615521855
Ptprz1	22.331497	1.2208824	1.83E-110	3.58E-108	0.521855486
Synpr	21.509987	1.1717576	1.26E-102	2.17E-100	0.592328278
Grik1	51.60389	2.8006966	0	0	0.809869376
Synpr	13.654084	1.4947845	1.91E-42	5.40E-40	0.597733711
Htr1f	14.4883995	2.0235941	1.43E-47	4.65E-45	0.396600567
Dcx	17.55026	1.7036071	5.92E-69	2.94E-66	0.481586402
Pde4b	7.4948606	1.4750395	6.64E-14	3.56E-12	0.359773371
Ablim3	14.010258	1.2661704	1.35E-44	3.92E-42	0.351274788

Table S7. Genes associated with memory impairment in specific cell subpopulations (related to Figure 5)

names	scores	logFC	p_vals	p_vals_adj	pct_nz_group	pct_nz_reference	Cluster
kcnip4	71.412766	1.145527	0	0	0.982025357	0.791630659	ExN1
Calb1	25.474173	0.4456326	3.8111E-143	2.5653E-141	0.275557695	0.142757429	ExN1
Ncam2	64.69853	1.2704473	0	0	0.783501846	0.410057834	ExN1
Calb1	4.320365	0.48023266	1.55771E-05	2.96333E-05	0.173921806	0.164216233	ExN3
Ncam2	7.703911	0.43544838	1.32E-14	3.59E-14	0.462313583	0.476000638	ExN3
Calb1	41.562603	1.2810899	0	0	0.550773369	0.149095607	ExN8
Ncam2	13.521291	0.25918862	1.17103E-41	6.70998E-40	0.671822461	0.465690497	ExN8
ptprz1	22.331497	1.2208824	1.83E-110	3.58E-108	0.521855486	0.258466871	InN3
Ptpro	21.09754	2.0316367	8.38E-99	6.24E-96	0.436260623	0.108497386	InN6
